# CysDuF database: annotation and characterization of cysteine residues in domain of unknown function proteins based on cysteine post-translational modifications, their protein microenvironments, biochemical pathways, taxonomy, and diseases

**DOI:** 10.1093/database/baag002

**Published:** 2026-01-23

**Authors:** Devarakonda Himaja, Debashree Bandyopadhyay

**Affiliations:** Department of Biological Sciences, Birla Institute of Technology and Science, Pilani, Hyderabad Campus, Jawahar Nagar Hyderabad, Kapra Mandal, Medchal Malkajgiri Dist., Telangana 500078, India; Department of Biological Sciences, Birla Institute of Technology and Science, Pilani, Hyderabad Campus, Jawahar Nagar Hyderabad, Kapra Mandal, Medchal Malkajgiri Dist., Telangana 500078, India

## Abstract

Experimental characterization and annotation of amino acids belonging to domains of unknown function (DUF) proteins are expensive and time-consuming, which could be complemented by computational methods. Cysteine, being the second most reactive amino acid at the catalytic sites of enzymes, was selected for functional annotation and characterization on DUF proteins. Earlier, we reported functional annotation of cysteine on DUF proteins belonging to the COX-II family. However, holistic characterization of cysteine functions on DUF proteins was not known, to the best of our knowledge. Here, we annotated and characterized cysteine residues based on post-translational modifications (PTMs), biochemical pathways, diseases, taxonomy, and protein microenvironment. The information on uncharacterized DUF proteins was initially obtained from the literature, and the sequence, structure, pathways, taxonomy, and disease information were retrieved from the SCOPe database using DUF IDs. Protein microenvironments (MENV) around cysteine residues from DUF proteins were computed using protein structures (*n* = 70 342). The cysteine PTMs were predicted using the in-house cysteine-function prediction server, DeepCys https://deepcys.bits-hyderabad.ac.in). The accuracy of the prediction, validated against known experimental cysteine PTMs (*n* = 18 626), was 0.79. The information was consolidated in the database (https://cysduf.bits-hyderabad.ac.in/), retrievable in downloadable formats (CSV, JSON, or TXT) using the following inputs, DUF ID, PFAM ID, or PDB ID. For the first time, we annotated cysteine PTMs in DUF proteins belonging to seven different biochemical pathways and various species across the taxonomy, notably for the SARS-CoV-2 virus. The nature of MENV around cysteine from DUF proteins was mainly buried and hydrophobic. However, in the SARS-CoV-2 virus, a significant number of functional cysteine residues were exposed on the surface with hydrophilic microenvironment.

## Introduction

**Figure 1:**
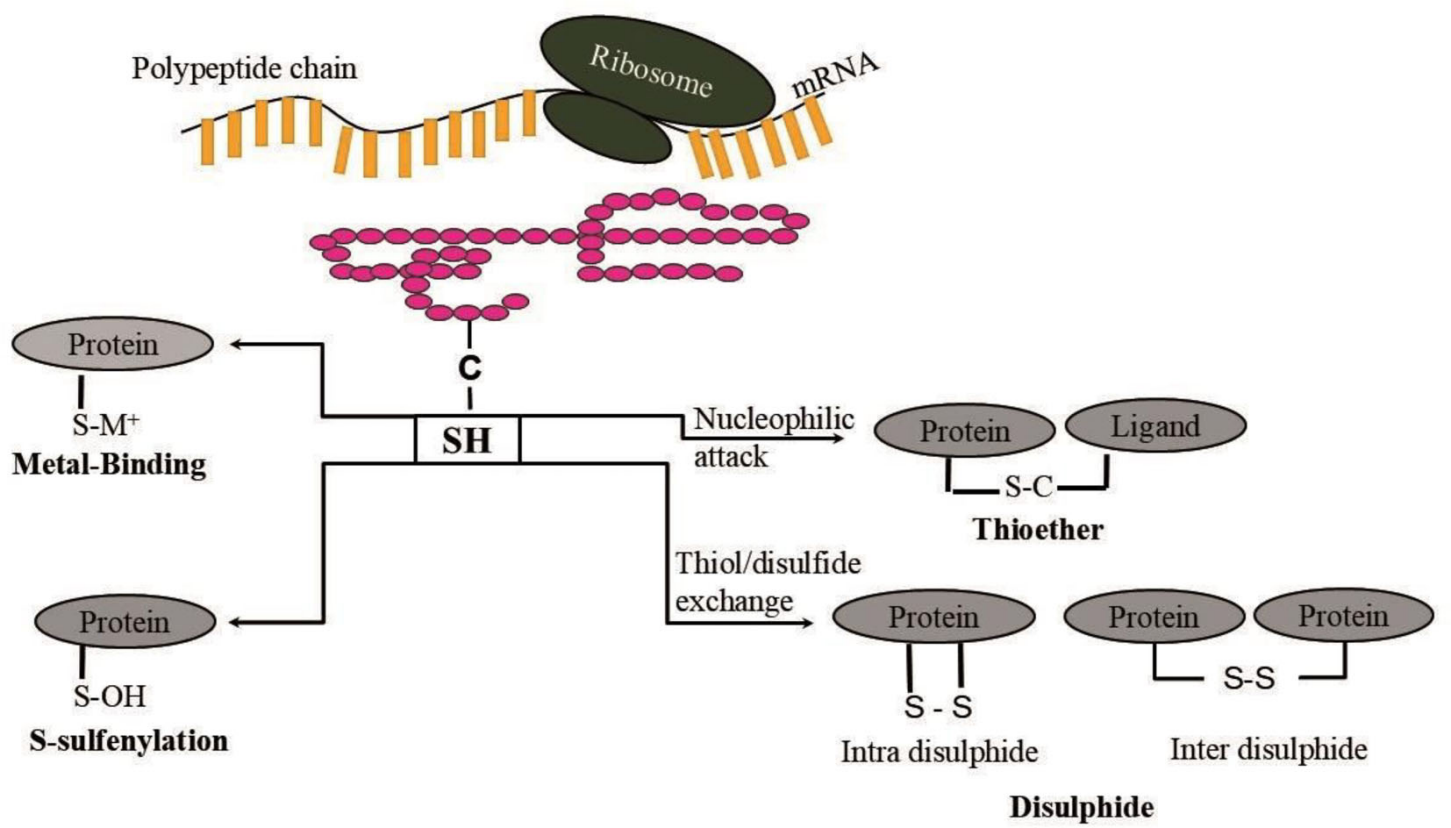
Schematic representation of four cysteine PTMs described in the CysDuF database.

Cysteine has unique chemical properties due to its reactive thiol group that undergoes a wide range of redox reactions and contributes towards various biological pathways. It can act as a nucleophile (S^−^) under physiological pH (pKa of cysteine thiol group is 8.1) and may serve as one of the key catalytic residues in many enzymes. Cysteine functions are broadly categorized into four groups, (i) Structural cysteines, (ii) metal-binding cysteines, (iii) catalytic cysteines, and (iv) regulatory cysteines [1]. The biological functions of cysteines include redox properties, binding to co-factors, scavenging reactive oxygen species (ROS), and reactive nitrogen species, scavenging toxic heavy metal ions, etc. Various cysteine oxidized products, such as sulfenic acid (SOH), sulfinic acid (SO_2_H), sulfonic acid (SO_3_H), disulphiide (-S-S-), glutathionylation (SSG), S-nitrosylation (SNO), etc., play a crucial role in redox hemostasis in the gut that has been profiled through pipelines [2] and chemical methods [3]. Imbalance in these cysteine post-translational modifications (Cys-PTMs) leads to oxidative damage within the gut and contributes towards gut ageing and disease conditions [4]. This variety of cysteine functions and their possible consequences make cysteine a suitable candidate for its function prediction in a given protein. With the advent of high-throughput screening, a large number of protein domains, domains of unknown function (DUFs), were sequenced, whose functions were uncharacterized. Experimental characterizations of amino acid functions for these DUF proteins were laborious and time-consuming. The computational approach could complement functional annotations of cysteine amino acids on DUF proteins. A total of 4775 DUF protein families were available in the PFAM database (v 35.0) [5], including both DUFs and uncharacterized protein families (UPFs) [5, 6]. ‘SUPFAM’ database curated all DUF proteins and provided the external link to the SCOPe database [7]. Similarly, the ‘PathFams’ database detected pathogen-assisted protein domains in DUF proteins [8]. The DUF proteins may belong to different biological functions, species, groups of organisms, or environmental conditions. Hence, the characterization of DUF protein function is crucial. DUF family proteins were reported to be involved in plant physiology, such as plant cell wall development, trichome development, plant stress responses, etc. [9, 10]. The disease-related DUF proteins were reported, such as pneumonia, neuronal diseases, viral infections, food-borne illnesses, fungal diseases, and many more [11]. DUF characterization was accelerated using computational techniques, such as phylogenetic tree, gene expression analysis, GO analysis, DALI Search Algorithm [12, 13], etc. Recently, bacterial signalling proteins, from DUF families, were characterized as GGDEF and EAL domains [14]. In *Oryza Sativa* (Rice), the function of the DUF568 was characterized using the phylogenetic tree, gene expression, GO analysis, co-expression, and protein–protein interaction (PPI) networks [15]. In *Plasmodium falciparum*, DUF proteins were characterized using DALI search on AlphaFold predictions. In *Agrobacterium tumefaciens*, DUF1127 was predicted to be involved in phosphate and carbon metabolism, using sequence similarity [16]. Similarly, DUF692 was annotated as multicellular non-heme iron-dependent oxidative enzymes, using sequence similarity [17]. Our recent study predicted PTMs of cysteine in the DUF proteins belonging to cytochrome C oxidase, subunit II-like transmembrane domains (COX II protein) [18]. ‘Unknome’ database reported experimentally annotated genes of the DUF proteins using RNA interference (RNAi) and knockdown techniques [19]. Apart from DUF sequences, only two PDB crystal structures are available for DUF proteins. However, there are many DUF-related protein crystal structures available in the PDB database [20]. Due to the unavailability of DUF PDB structures, the structural information was extracted from the DUF-related protein crystal structures, reported in the SCOPe database. To note, despite the availability of protein structures from other sources, like nuclear magnetic resonance (NMR) spectroscopy, electron microscope (EM) data, and artificial intelligence (AI) based structure predictions, like AlphaFold [21], DMFold [22], RosettaFold [23], etc., protein crystal structures provide the best resolution. Hence, only protein crystal structures are considered here. The structural information was required for the computation of local protein microenvironments and subsequent characterization of biochemical pathways, taxonomic distributions, diseases, etc. The protein microenvironment around cysteines from DUF-related proteins could be calculated based on the structures of the globular proteins only. The protein microenvironment is known to modulate various biological activities, including molecular recognition, PPIs, alteration of amino acid pKa values, hydration and dehydration properties, etc. [24–27]. Cysteine post-translational modifications were predicted on DUF proteins using a Deep Learning algorithm, DeepCys, based on protein structures and microenvironments [18]. DeepCys algorithm can predict only four Cys-PTMs, namely, disulphiide, metal-binding, thioether, and sulfenylation, as the training dataset used to develop DeepCys contains only these four modifications. The hypothesis in the current study is that protein microenvironment will modulate the cysteine PTMs in DUF-related proteins, their biochemical pathways, and related diseases. This hypothesis was tested on four cysteine PTMs that can be predicted by DeepCys (Fig. 1); seven biochemical pathways, electron transport chain (ETC), glutathione biosynthesis, Fe–S-cluster biogenesis, fatty acid synthesis, photosynthesis, Kreb’s cycle, and pentose phosphate pathway; and one hundred and fifty-six diseases within four taxonomic groups, according to NCBI Taxonomy [28]. The database would serve as a useful resource for cysteine functions in DUF proteins and their related information and analysis. It has a wide applicability to predict cysteine functions through web servers or bulk prediction using standalone code, available on GitHub (https://github.com/devhimd19/CysDUF).

## Methods

### DUF protein dataset curation

**Figure 2:**
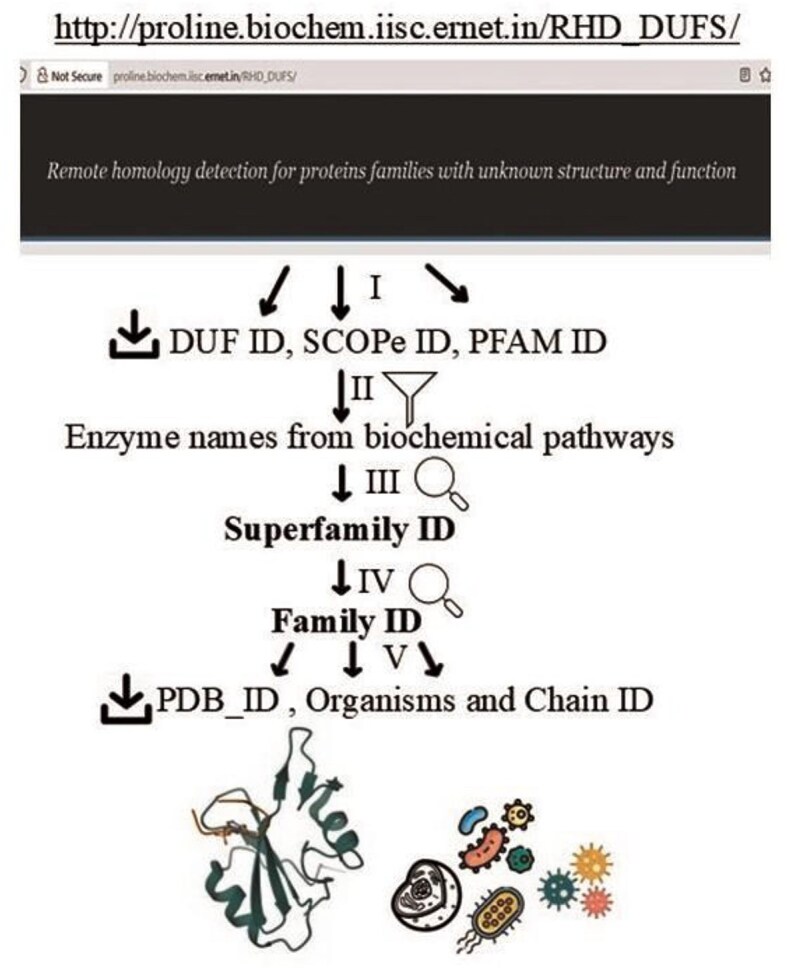
Steps of DUF data curation. (i) Extract and download a list of PFAM ID/DUF ID/SCOPe ID using search criteria, (a) pathway names and (b) catalytic cysteines [6, 7] (ii) filter the downloaded list using SCOPe superfamily resulting enzyme names from 7 biochemical pathways studied here; (iii) search SCOPe database with SCOP ID to extract superfamily ID; (iv) search SCOPe database with superfamily ID to extract family ID; (v) extract PDB ID per family ID.

DUF protein dataset was curated (22 May 2024) from the Superfamily (SUPFAM) database containing sequences and structures of protein domain families and superfamilies [7]. SUPFAM data was developed using five state-of-the-art remote similarity detection techniques to detect the relationship between uncharacterized DUFs and domain families of known structures. The study resulted 614 uncharacterized DUFs associated with a known structural domain from 54 families [6]. The known structural domains were linked to SCOPe database [29]. Beautifulsoup4 (version = 4.12.3), a Python library, was used to extract the DUF IDs and SCOPe information from SUPFAM database [6, 7] . The list of curated DUF proteins was filtered using two criteria. The first one was pathway names—ETC, glutathione metabolism, Fe–S-cluster biogenesis, fatty acid synthesis, photosynthesis, Kreb’s cycle, and Pentose phosphate pathway. The second criterium was catalytic cysteine in those pathways. The filtered information was saved in CSV format that contains the following columns, Pfam Accession (ID), DUF_ID, DUF name, and SCOPe ID. The SCOPe database [29] was searched to extract SCOPe superfamily ID, family ID, and PDB ID, sequentially. The flow of the data curation was shown schematically (Fig. 2). The PDB IDs were obtained from different experimental sources, namely, X-ray diffraction (*n* = 5835), NMR studies (*n* = 233), and electron microscopy (*n* = 68) (Fig. S1). The structures without reported experimental methods were discarded.

All information was concatenated and saved in CSV format. This CSV file was utilized to develop the web server.

A total of 74 DUF proteins (Table 1), 6218 PDB IDs (Table S1), and 70 342 cysteine residues were reported. The maximum number of cysteine residues belonged to the ETC (*n* = 29 638), followed by glutathione metabolism (*n* = 26 656), Fe–S cluster biogenesis (*n* = 24 826), fatty acid synthesis (*n* = 9229), photosynthesis (*n* = 1145), Kreb’s cycle (*n* = 27), and pentose phosphate pathway (*n* = 18). The biochemical pathway information was curated from the SUPFAM database.

**Table 1:** List of DUF IDs and biochemical pathway names, curated from the SUPFAM database.

S. N	Duf id	Biochemical pathways
1	DUF459	Electron transport chain
2	DUF460	Electron transport chain
3	DUF461	Electron transport chain
4	DUF462	Electron transport chain
5	DUF463	Electron transport chain
6	DUF464	Electron transport chain
7	DUF465	Electron transport chain
8	DUF466	Electron transport chain
9	DUF467	Electron transport chain
10	DUF468	Electron transport chain
11	DUF455	Electron transport chain, Fe–S-cluster biogenesis
12	DUF1863	Electron transport chain
13	DUF3050	Electron transport chain
14	DUF3291	Electron transport chain, glutathione metabolism, Fe–S-cluster biogenesis
15	DUF1636	Electron transport chain, glutathione metabolism, Fe–S-cluster biogenesis
16	DUF4405	Electron transport chain
17	DUF3182	Fatty acid synthesis and glutathione metabolism
18	DUF2764	Electron transport chain
19	DUF1175	Fatty acid synthesis
20	DUF521	Krebs cycle and Fe–S-cluster biogenesis
21	DUF2298	Electron transport chain
22	DUF1015	Electron transport chain
23	DUF4173	Photosynthesis
24	DUF137	Electron transport chain
25	DUF2652	Electron transport chain, glutathione metabolism, Fe–S-cluster biogenesis
26	DUF1691	Electron transport chain
27	DUF3611	Electron transport chain
28	DUF899	Electron transport chain, glutathione metabolism, Fe–S-cluster biogenesis
29	DUF3088	Electron transport chain, glutathione metabolism, Fe–S-cluster biogenesis
30	DUF1574	Electron transport chain
31	DUF4343	Fatty acid synthesis and glutathione metabolism
32	DUF1287	Fatty acid synthesis
33	DUF2214	Electron transport chain
34	DUF2272	Fatty acid synthesis
35	DUF4300	Fatty acid synthesis
36	DUF1624	Electron transport chain, glutathione metabolism, Fe–S-cluster biogenesis
37	DUF2919	Electron transport chain
38	DUF2231	Electron transport chain
39	DUF4142	Electron transport chain, Fe–S-cluster biogenesis
40	DUF2165	Electron transport chain
41	DUF1352	Electron transport chain
42	DUF3483	Electron transport chain
43	DUF4344	Electron transport chain
44	DUF4188	Electron transport chain, glutathione metabolism, Fe–S-cluster biogenesis
45	DUF1111	Electron transport chain
46	DUF2338	Pentose phosphate pathway
47	DUF2339	Pentose phosphate pathway
48	DUF2340	Pentose phosphate pathway
49	DUF2340	Electron transport chain
50	DUF420	Complex IV of electron transport chain
51	DUF3581	Fatty acid biosynthesis
52	DUF4333	Complex III of electron transport chain
53	DUF2387	Electron transport chain
54	UPF0203	Complex III of electron transport chain
55	DUF1120	Complex III of electron transport chain
56	DUF1298	Fatty acid synthesis
57	UPF0547	Electron transport chain
58	DUF3613	Complex III of electron transport chain
59	DUF2872	Electron transport chain
60	DUF1451	Electron transport chain
61	DUF4523	Electron transport chain, glutathione metabolism, Fe–S-cluster biogenesis
62	DUF2414	Electron transport chain, glutathione metabolism, Fe–S-cluster biogenesis
63	DUF2414	Photosynthesis
64	DUF4174	Electron transport chain, glutathione metabolism, Fe–S-cluster biogenesis
65	DUF4350	Electron transport chain
66	DUF1450	Electron transport chain, glutathione metabolism, Fe–S-cluster biogenesis
67	DUF973	Photosynthesis
68	DUF1610	Electron transport chain
69	DUF1440	Electron transport chain
70	UPF0180	Electron transport chain
71	DUF2194	Electron transport chain
72	DUF2296	Electron transport chain
73	DUF779	Fe–S-cluster biogenesis
74	DUF2827	Uronic acid pathway

There were eight cell organelles (cytoplasm, mitochondria, thylakoid membrane, periplasm, ROD Outer Segment (Eye), chloroplast, cell membrane, and nucleus) reported in the database. The cell organelle location information was curated from the PDB database.

### Computation of cysteine protein microenvironment embedded in the DUF proteins

**Figure 3:**
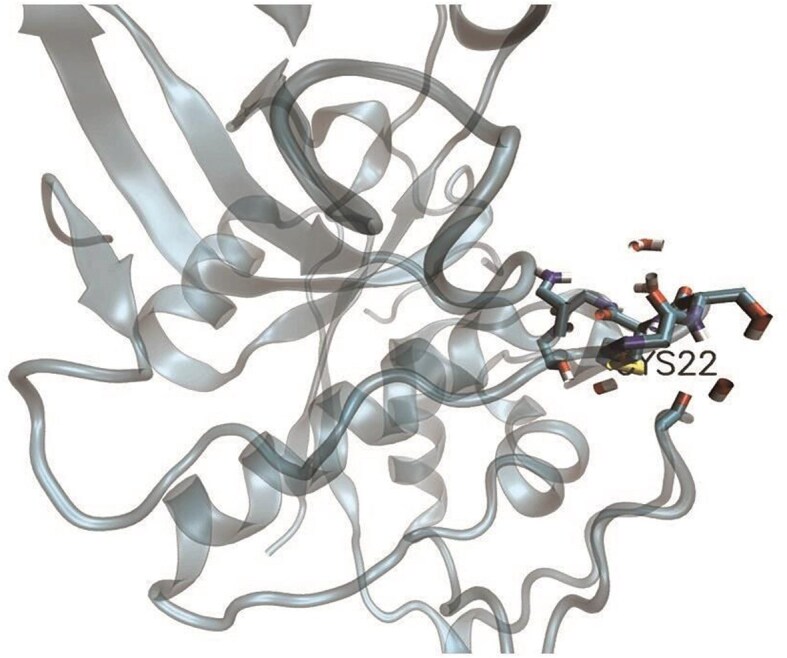
Depiction of cysteine (Cys^22^), part of a disulfide bridge (PDB ID:8PCH). Protein microenvironment (4.5 Å radius) around Cys22 is depicted, in stick representation. The protein background is shown as cartoon representation. The figure was generated using VMD software.

The protein microenvironments (MENV) around 70 342 cysteine thiol groups embedded in DUF proteins were computed using crystal structures. The cysteine protein microenvironment (three-dimensional spatial arrangement around cysteine amino acid) was quantified as the summation of the hydrophobic/hydrophilic contributions (estimated by Rekker’s fragmental constants) [30] from the protein structure encompassed within the first contact shell (approximately 4.5 Å radius) [24] (Fig. 3). The weighted summation of the Rekker’s fragments constants within the first contact shell of the cysteine amino acid was termed Hpy^A^ (Eq. 1) [24]. Similarly, Hpy^s^ was expressed as the weighted summation of the Rekker’s fragmental constants of solvent molecules within the first contact shell. Hpy^s^ was derived from molecular dynamics simulations with TIP3P water models [31]. Summation of Hpy^A^ and Hpy^s^, weighted by the buried fraction (BF) (ζ) was reported as total Hpy (THpy) (Eq. 3) [24]. The final property descriptor, the relative hydrophobicity, rHpy, was obtained by normalizing THpy by Hpy^s^. The rHpy quantity is an intrinsic property and is independent of the size of an amino acid.

Although the MENV calculation needed protein Cartesian coordinates from any source, such as X-ray crystallography, NMR, SAXS, molecular modelling, etc., in this database, we selected only crystallography data. The input to the protein microenvironment, encoded in the FORTRAN language, was a three-dimensional structure, and the outputs were (i) BF and (ii) rHpy [24]. The BF was defined as the fraction of the surface of the functional group embedded within the protein [32]; that ranges from zero to one; zero BF indicates the thiol group is completely exposed to the solvent, and vice versa. The upper limit of rHpy was formulated as one indicating the cysteine thiol group was completely immersed in the aqueous solvent. There was no lower limit of rHpy; slight variations in the lower limits were observed depending on the dataset, for example, −0.3 [24] to −0.4 [25]. The BF and rHpy together constituted protein microenvironment space around a cysteine thiol group.

### Prediction of cysteine PTMs in the DUF proteins

Cysteine PTMs were predicted using the prediction server, DeepCys, based on a deep neural network and trained on protein crystal structures [18]. Inputs to DeepCys were—the PDB ID of the DUF protein, chain ID, and the cysteine residue number. DeepCys, being a multiple cysteine function prediction tool, outputs probabilities of four cysteine PTMs, namely, disulfide, S-sulphenylation, thioether, and metal-binding. However, there are many more Cys-PTMs, such as Glutathionylation, nitrosylation, persulfidation, etc., that have significant contributions to protein structure and stability, redox balance, etc.

### Clustering the protein microenvironment space around the cysteine thiol group

The protein microenvironment space around the cysteine thiol group was clustered using agglomerative hierarchical clustering [33] implemented in a Python script and enabled with Scikit-Learn (1.1.1) and Matplotlib (3.5.3) libraries [34]. Protein microenvironment space was divided into smaller bins of equal spacing [buried fraction = 0.1, rHpy = 0.1]. The clustering was done by using the subsampling method, where only 10% subsample was employed in the Python code. The agglomerative hierarchical clustering initially considers each bin as a single cluster. The final clusters were defined based on the proximity of a data point (BF, rHpy) to its nearest cluster center. The agglomerative hierarchical clustering resulted in three clusters.

## Results

### Prediction of cysteine PTMs in CysDuF database

The DUF proteins curated in the CysDuF database were related to experimentally solved structures; however, the protein functions were not annotated. Four cysteine functions were predicted, here, using the in-house cysteine function prediction server DeepCys, based on protein structures. Several local and global protein properties, like sequence and secondary structure motifs, BFs, protein microenvironments, and enzyme classes, were extracted for each protein from the protein data bank (PDB) file to develop the DeepCys model. By design, DeepCys can predict any one of the four cysteine functions for a given protein, namely, disulfide, thioether, S-sulfenylation, or metal-binding. Out of 70 342 cysteines in the DUF database, the majority were predicted as thioether or metal-binding (Table 2). To note, the maximum number of cysteine residues in this database belonged to the ETC. In Complex III of the ETC, thioether modification was reported [35, 36]. Cysteine thioether modification was also reported In the glutathione metabolism [37], fatty acid biosynthesis [38], Kreb’s cycle [39], and pentose phosphate pathway [40]. In Complex IV of ETC, the cysteine residues from DUF proteins were mainly predicted for two modifications, metal binding and disulfide [18]. Other Cys-PTMs present in Complex IV of ETC, namely, glutathionylation, nitrosylation, or persulfidation [41], cannot be predicted using the current DeepCys tool. We have compared our predicted results with the ground truth (experimental results) reported in the respective PDB header files.

**Table 2:** Validation of the predicted PTMs of DUF cysteines (using DeepCys) with the experimental PTMs (from PDB header files).

Cysteine PTM	Number of experimental cysteine PTM	Number of PTMs predicted using DeepCys	Precision	Recall	*F*1-score
Thioether	1853	9154	0.19	0.94	0.31
Metal-binding	5615	2774	0.77	0.38	0.51
Disulfide	11 116	5605	0.91	0.46	0.61
Glutathionylation	41	0	0	0	0
S-sulphenylation	0	1093	0	0	0
Total	18 626	18 626			
Macroavg			0.37	0.35	0.28
Weighted average			0.79	0.48	0.55

### Validation of the predicted PTMs based on the experimental observations

Predicted cysteine PTMs were validated with the experimental findings reported in the respective PDB header files. There were only 18 626 experimental PTMs reported for 70 302 cysteine in DUF proteins (Table 2).

**Figure 4:**
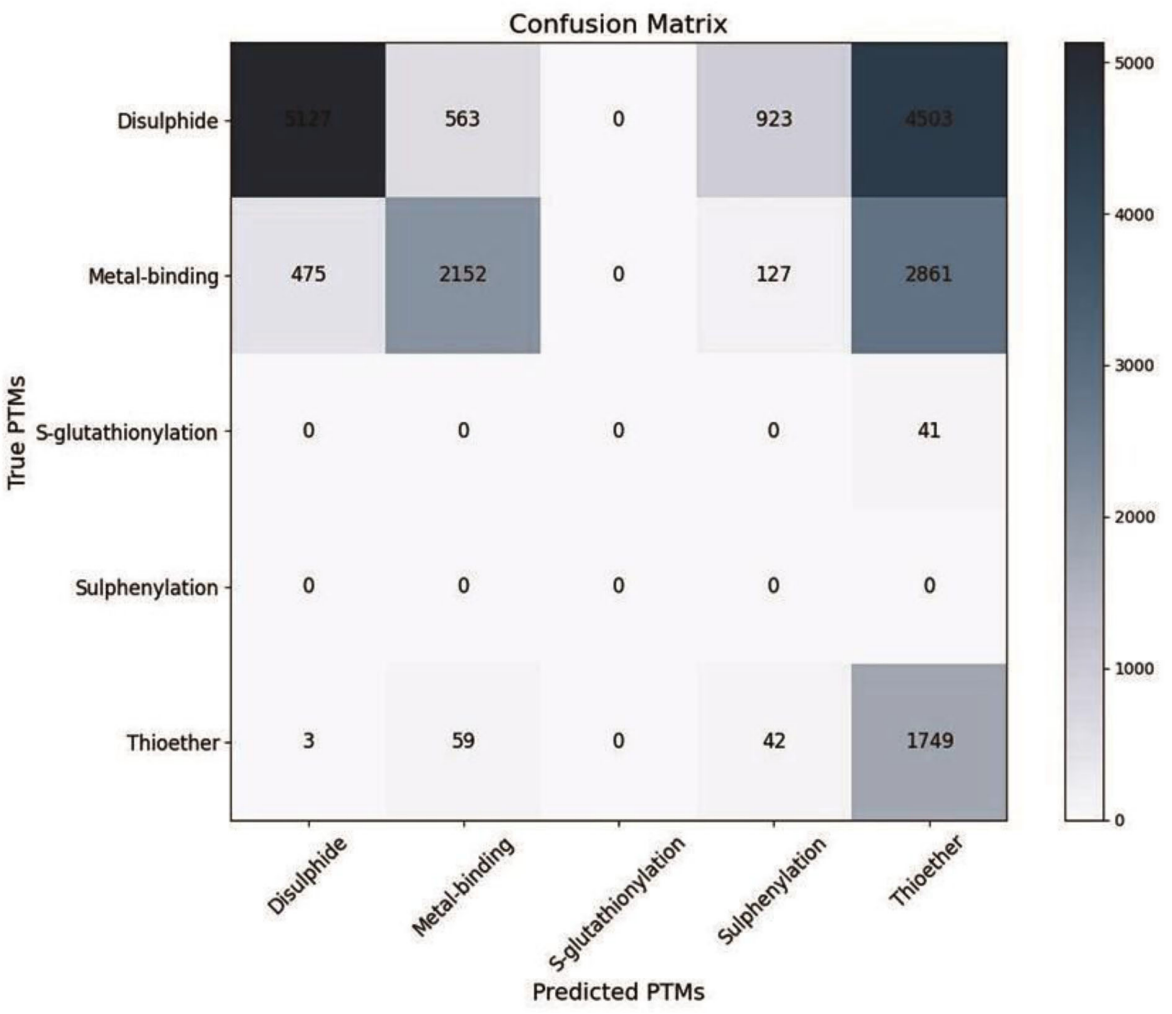
Confusion matrix to validate the predicted cysteine PTMs (using DeepCys software) with the experimental (PDB header file) observations. The heatmap indicates the range of cysteine numbers.

Hence, the validation was restricted to 18 626 cysteines only. Four different experimental cysteine PTMs were reported, namely, disulfide, metal-binding, thioether, and glutathionylation. Whereas the cysteine PTM prediction software, DeepCys, predicted disulfide, metal-binding, thioether, and sulfenylation, only. The prediction was evaluated using the confusion matrix (Fig. 4). This matrix was generated from the experimental and predicted cysteine PTM numbers (Table 2). Several evaluation metrics were used to validate the prediction performances, namely, precision, recall, *F*1-score, accuracy, macro average (macroavg), and weighted average (Supplementary, Eq. 1–5). The prediction performances of different cysteine PTMs varied (Table 2). The overall accuracy of prediction was 0.79. The prediction of true positives over false positives (precision) was the best for disulfide and metal-binding. Whereas, the prediction of true positives over false negatives was the best predicted for thioether. To note, S-glutathionylation has no predictions reported, and S-sulfenylation has no experiments reported. sulfisulfi

### Diversity of protein microenvironments around Cys-PTMs and in different biochemical pathways

From our earlier investigations, we observed that Cys residues were embedded in three different types of protein microenvironments, buried hydrophobic, intermediate, and exposed hydrophilic [26]. Here we explored two questions, (i) whether diversity in the protein microenvironment existed around cysteine in this database, and (ii) if it existed, whether there were preferential cysteine protein microenvironments towards different PTMs, pathways, and diseases.

**Figure 5:**
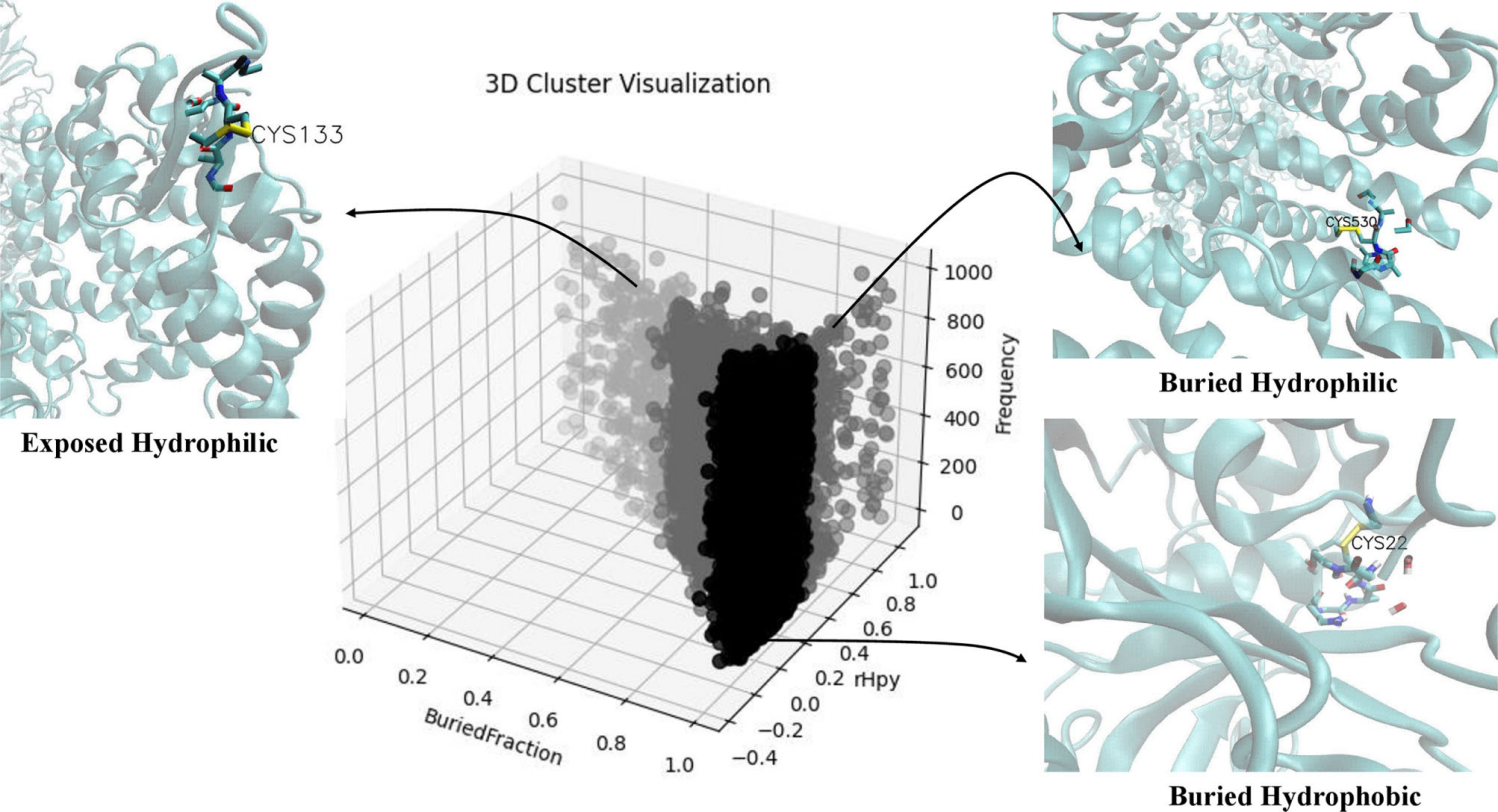
Distribution of cysteine protein microenvironments, from DUF proteins, in three clusters, buried hydrophobic, buried hydrophilic, and exposed hydrophilic. The X-axis represents the BF; the Y-axis, rHpy; and the Z-axis, populations of cysteine. Three insets show the relative position of the cysteine residue in three different protein microenvironments, buried hydrophobic (PDB ID: 8PCH), buried hydrophilic (PDB ID:7XAZ), and exposed hydrophilic (PDB ID:7UON). The figure was generated using Matplotlib and VMD software.

The first question was addressed by clustering the protein microenvironment (MENV) space around all the cysteine residues in the database. Two parameters, BF and microenvironment property descriptor (rHpy), were used to cluster MENV space, using agglomerative clustering (Fig. 5). The three-dimensional representation of the protein structures using visual molecular dynamics (VMD) tool [42] revealed the relative positions of cysteine residues in different protein microenvironments. The largest cluster denoted that the cysteine MENV was deeply buried in the protein core (high average BF value of 0.98) and significantly hydrophobic (low average rHpy value of 0.08) (Table 3); hence, named as ‘buried-hydrophobic’. To note, according to the definition of BF described in the method section, BF value of one indicated that the residue was fully buried inside the protein, and vice versa. Similarly, rHpy value of 1 indicated totally hydrophilic microenvironment. The second largest cluster exhibited a relatively high average BF (0.81) and moderate average rHpy value (0.38), indicating that the cysteine residue, despite being buried inside the protein, has a relatively hydrophilic protein microenvironment around it. This cluster appeared to be buried in nature yet hydrophilic, hence termed as, ‘buried-hydrophilic’. In one of our previous studies, a similar microenvironment cluster was reported that was more exposed (average BF, 0.77) to the solvent than the ‘buried-hydrophilic’ cluster and also more hydrophilic (0.40); hence, it was classified as an ‘intermediate cluster’ [26]. The least populated cluster was ‘exposed-hydrophilic,’ where the average BF of the Cys was 0.39, and the average rHpy was 0.68.

**Table 3:** Statistics (average value) of cysteine microenvironment clusters.

Cluster type	Average BF (σ)	Average rHpy (σ)	Average distance to centroid (Å)	No of cysteines in each cluster	No of PDB IDs in each cluster
Buried hydrophobic	0.97 (0.03)	0.08 (0.12)	0.11	4517	2207
Buried hydrophilic	0.81 (0.12)	0.37 (0.14)	0.15	2160	1333
Exposed hydrophilic	0.39 (0.12)	0.67 (0.09)	0.14	366	294

The standard deviation (σ) is given within parentheses.

The second question was answered by comparing the normalized populations of different Cys-PTMs across the microenvironment clusters (Table 4). The overall trend showed that all four modifications were maximally populated in the ‘buried-hydrophobic’ cluster, followed by ‘buried-hydrophilic’ and ‘exposed-hydrophilic’, similar to the cysteine microenvironment distribution (Table 3) and that reported elsewhere [24].

**Table 4:** Normalized cysteine populations of different post-translation modifications across microenvironment clusters.

Cluster type	Disulphide	Metal-binding	Thioether	S-sulphenylation
Buried hydrophobic	0.62	0.66	0.63	0.62
Buried hydrophilic	0.35	0.29	0.30	0.28
Exposed hydrophilic	0.02	0.04	0.05	0.08

The cysteine population within the cluster was normalized by the number of cysteine residues per PTMs.

**Figure 6:**
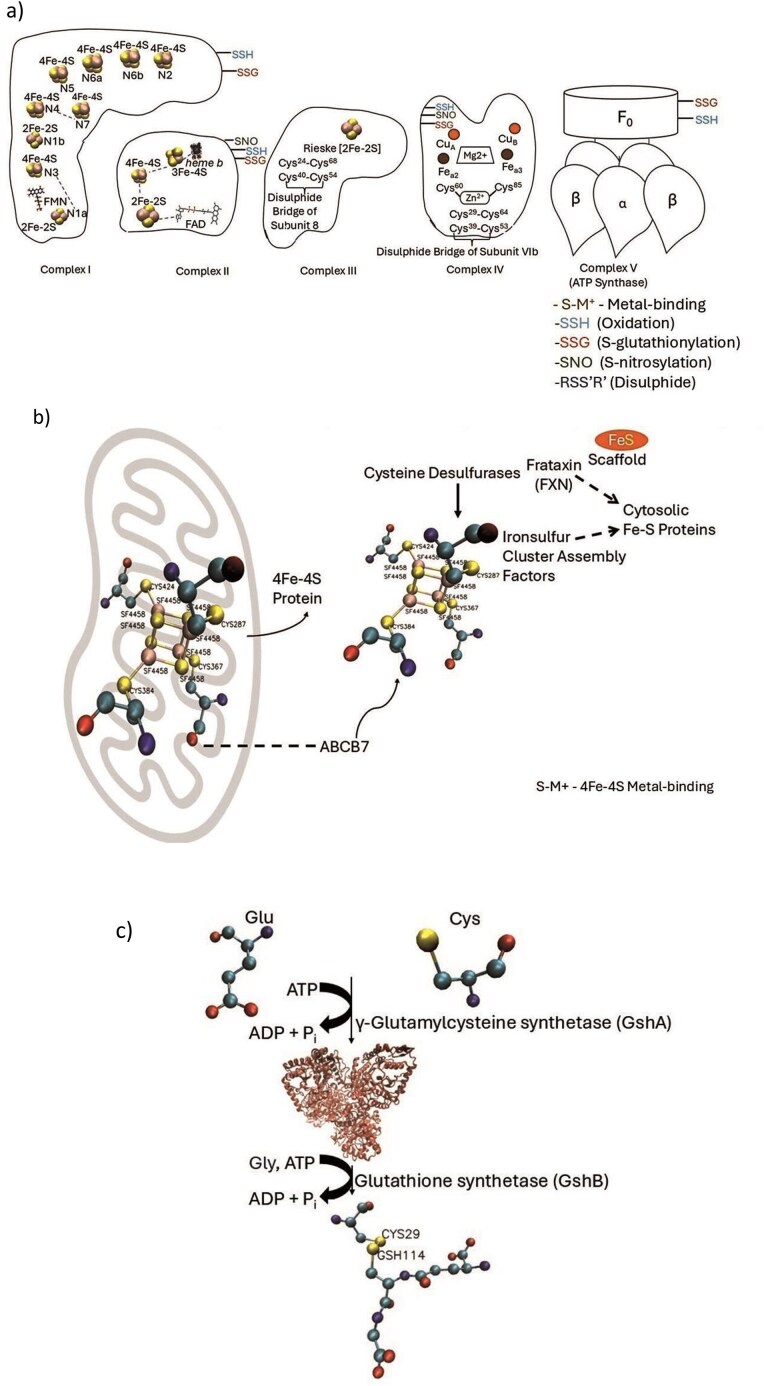

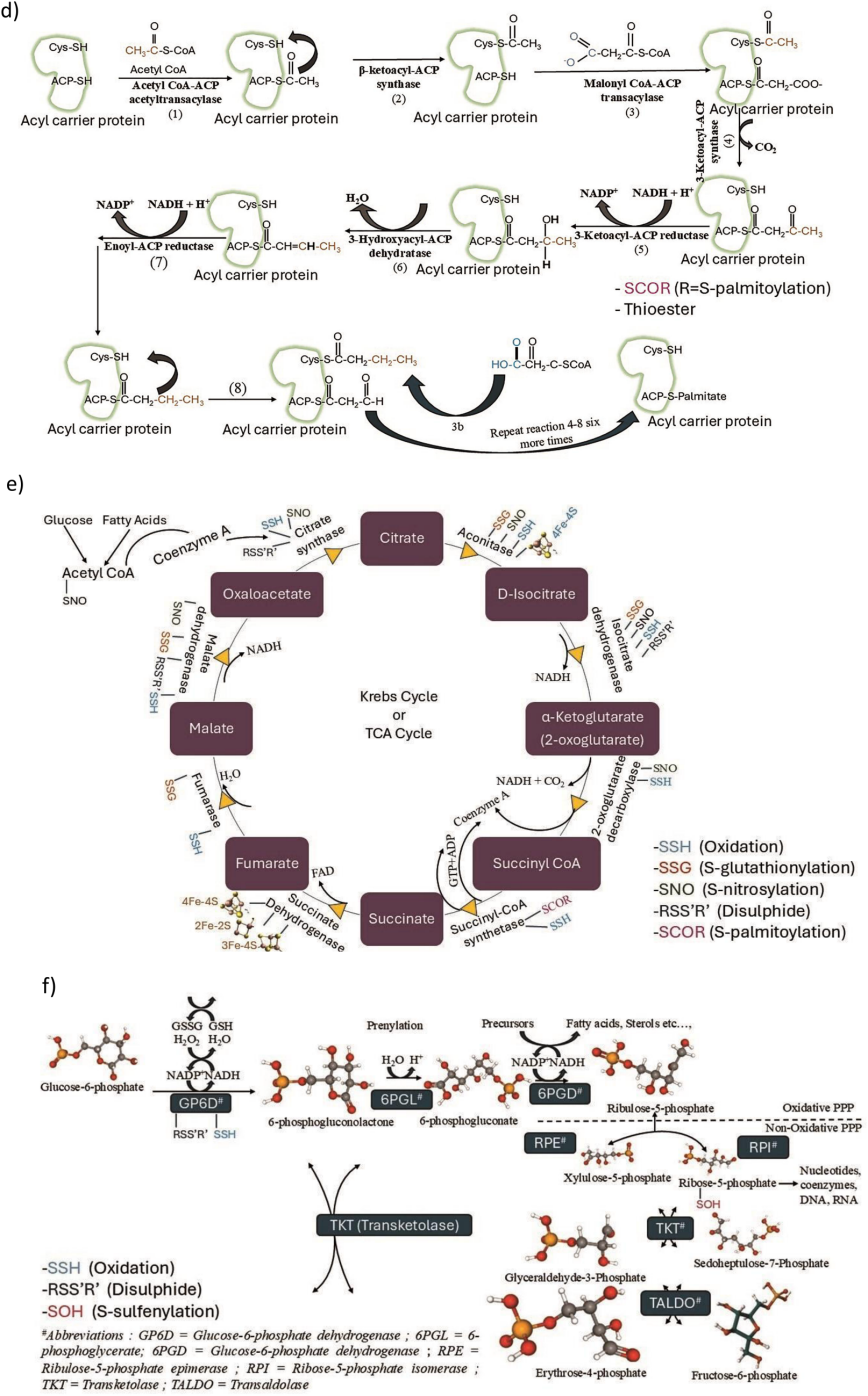
Schematic representations of Cys-PTMs in different pathways curated from literature, (a) ETC, (b) Fe–S cluster biogenesis, (c) glutathione biosynthesis, (d) fatty acid biosynthesis, (e) Kreb’s cycle, (f) pentose phosphate pathways. The amino acids and protein structures were depicted using VMD software. The small molecules were obtained from PubChem database. Cartoon diagrams were created using Microsoft PowerPoint.

The preferences of cysteine protein microenvironments towards seven biological pathways were studied by comparing the normalized populations of different cysteine microenvironment clusters (Table 5). The cysteine microenvironment was maximally populated in the ‘buried-hydrophobic’ region in all the pathways, agreeing with the hydrophobic nature of the cysteine residue. However, in the photosynthetic pathway the maximum cysteine microenvironment was populated in the ‘buried-hydrophilic’ region. Similarly, Kreb’s cycle also has a large proportion of cysteine embedded in the ‘buried-hydrophilic’ microenvironment. There were six cysteines from the Kreb’s cycle (Table S2) and sixty-eight from photosynthesis (Table S3), all embedded in buried-hydrophilic microenvironments. In Kreb’s cycle, all six functional cysteine residues embedded in the buried-hydrophilic microenvironment were from the aconitase enzyme [41]. To note, it has been reported that the Fe–S clusters in aconitase have a hydrophilic microenvironment created by the polar groups [43] that matched our current observations. In photosynthesis, the functional cysteines embedded in the buried-hydrophilic microenvironment mainly belong to photosynthetic reaction center II proteins (like proteins D1, D2, CP43, CP47, cytochrome C subunit), cytochrome c-550, etc. To note, most of the photosystem II proteins were membrane proteins and not globular proteins. Whereas the MENV computation was designed only for globular proteins, where the surface of a protein was exposed to water molecules, in contrast to membrane proteins, exposed to the lipid bilayer. Hence, the predictions of membrane proteins are likely to be less accurate compared to the globular proteins. The Cys-PTMs predicted were thioether, metal-binding, and sulfenylation (one of the cysteine oxidations). The experimentally curated ones were metal binding (as Fe–S cluster), and cysteine oxidations (Fig. 6). The protein and amino acid structures were depicted using VMD [42], and the small molecules were curated from PubChem database [44]. Thus, DeepCys-predicted and experimental Cys-PTMs, fairly, agree with each other.

**Table 5:** Normalized cysteine populations in different biological pathways across microenvironment clusters. Pathways having higher normalized cysteine populations in buried hydrophilic cluster for different pathways were highlighted in bold

Cluster type	Electron transport chain	Glutathione metabolism	Fe–S-cluster biogenesis	Fatty acid synthesis	Photosynthesis	Krebs cycle	Pentose phosphate pathway
Buried hydrophobic	0.60	0.74	0.60	0.73	0.42	0.57	0.50
Buired hydrophilic	0.33	0.22	0.33	0.24	**0.54**	**0.42**	0.25
Exposed hydrophilic	0.06	0.03	0.06	0.02	0.03	0.00	0.25

The cysteine population within the cluster was normalized by the number of cysteines per biological pathway.

### Diversity of Cys-PTMs and their microenvironments across different taxonomic kingdoms

**Figure 7:**
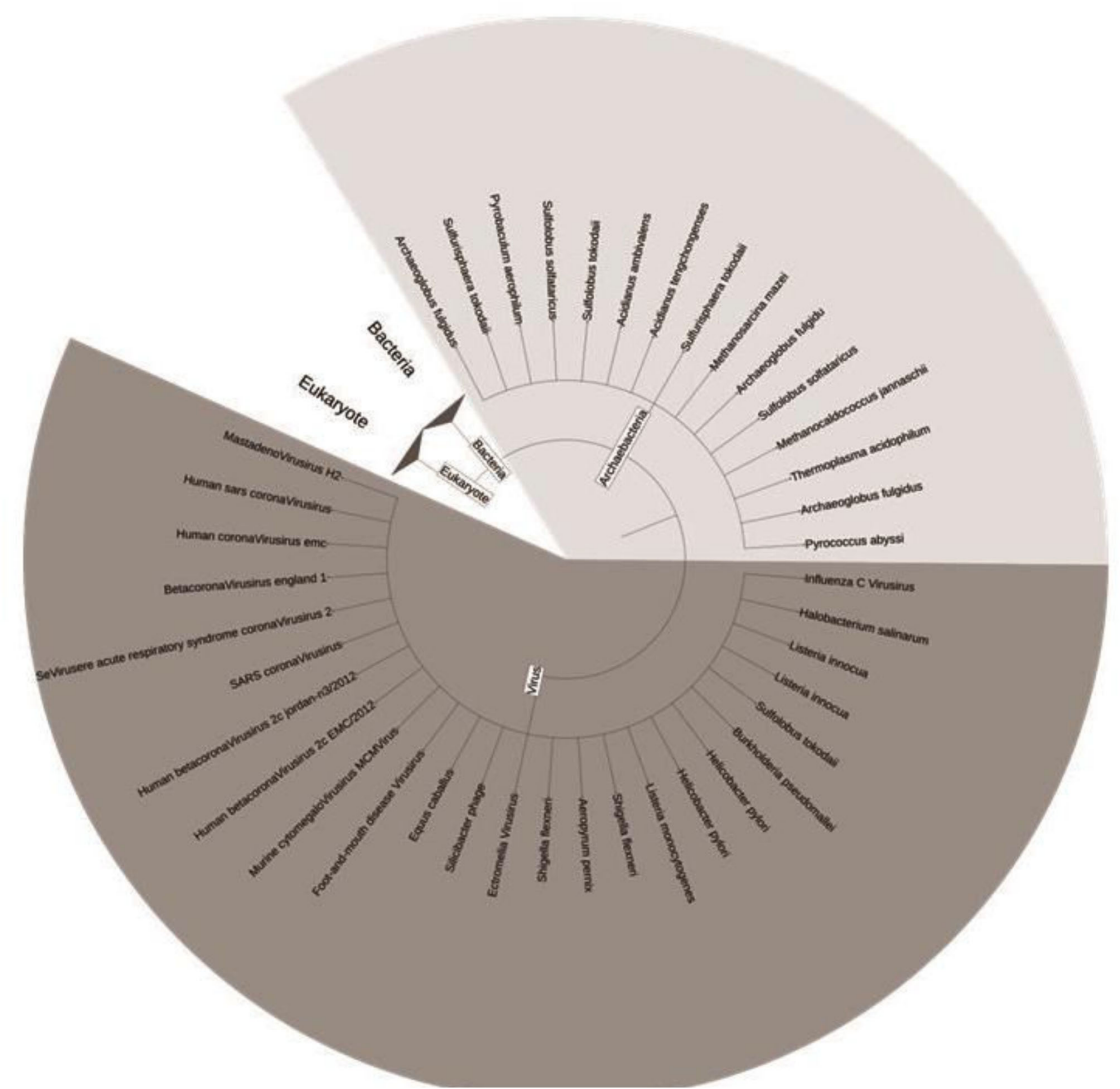
Simple tree representing the species in this study based on taxonomy for virus and archaebacteria. The figure was generated using ITOL version 7.

**Figure 8:**
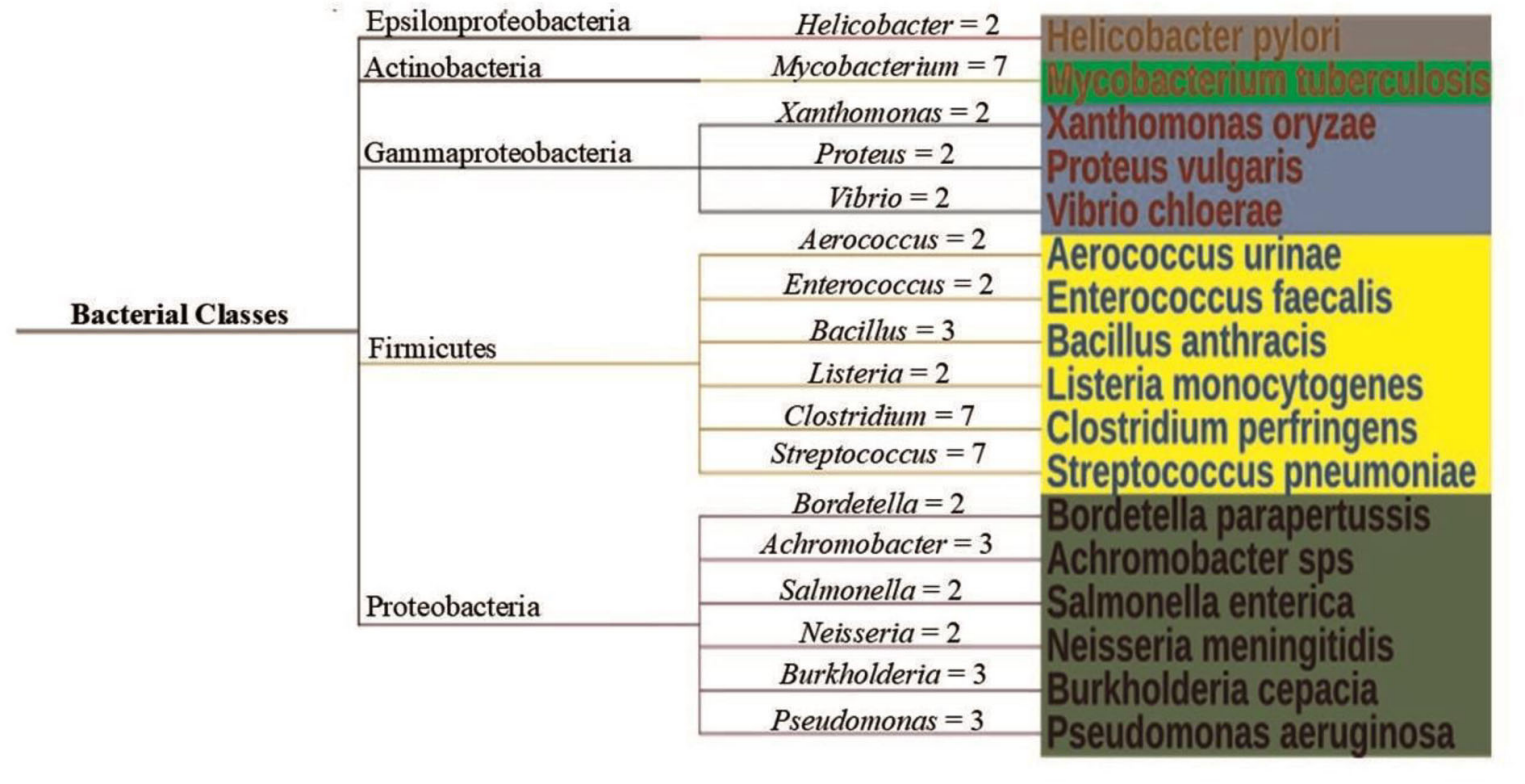
Simple tree for disease-causing bacteria, classified according to their taxonomy. The number of species per genera is shown on the connecting branch. One example per genera is shown for clarity. The figure was generated using ITOL version 7.

The DUF proteins were classified into four different taxonomic kingdoms, namely bacteria, archaebacteria, viruses, and eukaryotes, as per NCBI Taxonomy [45]. A total of 607 organisms were reported in this database. Simple trees were constructed (using Interactive Tree of Life (ITOL) version 7 software [46]) to represent the taxonomic variations for virus and Archaebacteria (Fig. 7), and Eukaryotes and bacteria (Fig. S2). The highest number of species was observed for Bacteria, both pathogenic and non-pathogenic (*n* = 342). The disease-causing bacterial species, classified according to their taxonomy were represented by a simple tree (Fig. 8). The complete list of the species names and corresponding diseases was shown (Table S4). The literature report also suggested that most of the DUF proteins belonged to kingdom bacteria [11]. The second largest kingdom in this database was Eukaryotes. The DUF proteins from Kingdom Virus (*n* = 25), were reported for the first time. All the viruses reported were disease-causing (Table S4). Here, we explored the diversity of Cys-PTMs and their microenvironments across different taxonomic kingdoms by comparing the normalized populations of cysteine microenvironment clusters across the kingdoms (Table 6). Proteins from all the kingdoms exhibited the highest populations in buried-hydrophobic cluster, complementing the hydrophobic nature of the cysteine residue. A significant population of ‘buried-hydrophilic’ microenvironment was observed around proteins from Archaebacteria and Bacteria. This could presumably be attributed to the extremophile nature of bacteria (*n* = 139) out of 146 cysteine in the same cluster. Interesting to note the presence of exposed-hydrophilic cluster in viruses despite its negligible presence in other kingdoms. This observation plausibly indicated the possible exposure of the catalytic cysteine residues on the viral protein surfaces.

**Table 6:** Normalized cysteine populations in different kingdoms across microenvironment clusters.

Domain kingdom	Eukaryotes	Archaebacteria	Viruses	Bacteria
Buried hydrophobic	0.66	0.53	0.55	0.53
Buried hydrophilic	0.29	**0.40**	0.31	**0.38^#^**
Exposed hydrophilic	0.03	0.06	0.13*	0.08

The cysteine population within the cluster was normalized by the number of cysteines, per kingdom. Significant numbers are reported in bold. The pathogens are named in the footnote.

*Coronavirus.; ^#^*Clostridium botulinum, Mycobacterium tuberculosis, Shewanella frigidimarina*.

### Diversity of Cys-PTMs and their microenvironments across different diseases

Cysteine plays a key role at the enzyme catalytic sites and also in balancing the cell redox chemistry. Several Cys-PTMs were identified on the viral proteins crucial for viral proliferation and propagation. For example, 147 cysteine residues having three different Cys-PTMs (namely, disulfide, metal-binding, and sulfenylation) were identified on the SARS-COV-2 genome, from the ViralZone of the Expasy database (https://viralzone.expasy.org/8996). The gene segments containing these Cys-PTMs were replicase polyprotein 1a (pp1a), ppa1b, spike glycoprotein, open reading frame (ORF)7a, and ORF8. Hence, understanding the role of these Cys-PTMs on the viral life cycle and infectivity is crucial. This kind of systematic search to establish the relationship between diseases and Cys-PTMs was not done earlier.

DUF proteins involved in viral diseases (*n* = 10) were classified as Animal-inherited diseases specifically infecting humans (Table S4). The DUF proteins related to SARS-COV-2 virus causing lung diseases were reported for the first time, in this database. A few fungal diseases (*n* = 8) associated with DUF proteins were reported here those mainly invade plants. The parasitic (worm) infections (*n* = 14), were caused by liver fluke (*n* = 5), hookworm (*n* = 2), and parasitic worm (*n* = 7) (Table S4). The protozoan diseases (*n* = 15) reported in this DUF database were mostly animal-inherited (*n* = 13). Two human protozoan diseases were reported causing Gastric, by *Entamoeba histolytica* (*n* = 1) and sexually transmitted diseases/urinary tract infections, caused by *Trichomonas vaginalis* (*n* = 1). There were eight plant diseases (*n* = 8) reported here caused by bacteria and fungi.

Here we explored twenty diseases reported in CysDuF database caused by 156 different species. Most of those were bacterial species (*n* = 101). The full list of pathogens and the diseases caused by those are reported (Table S4).

**Figure 9:**
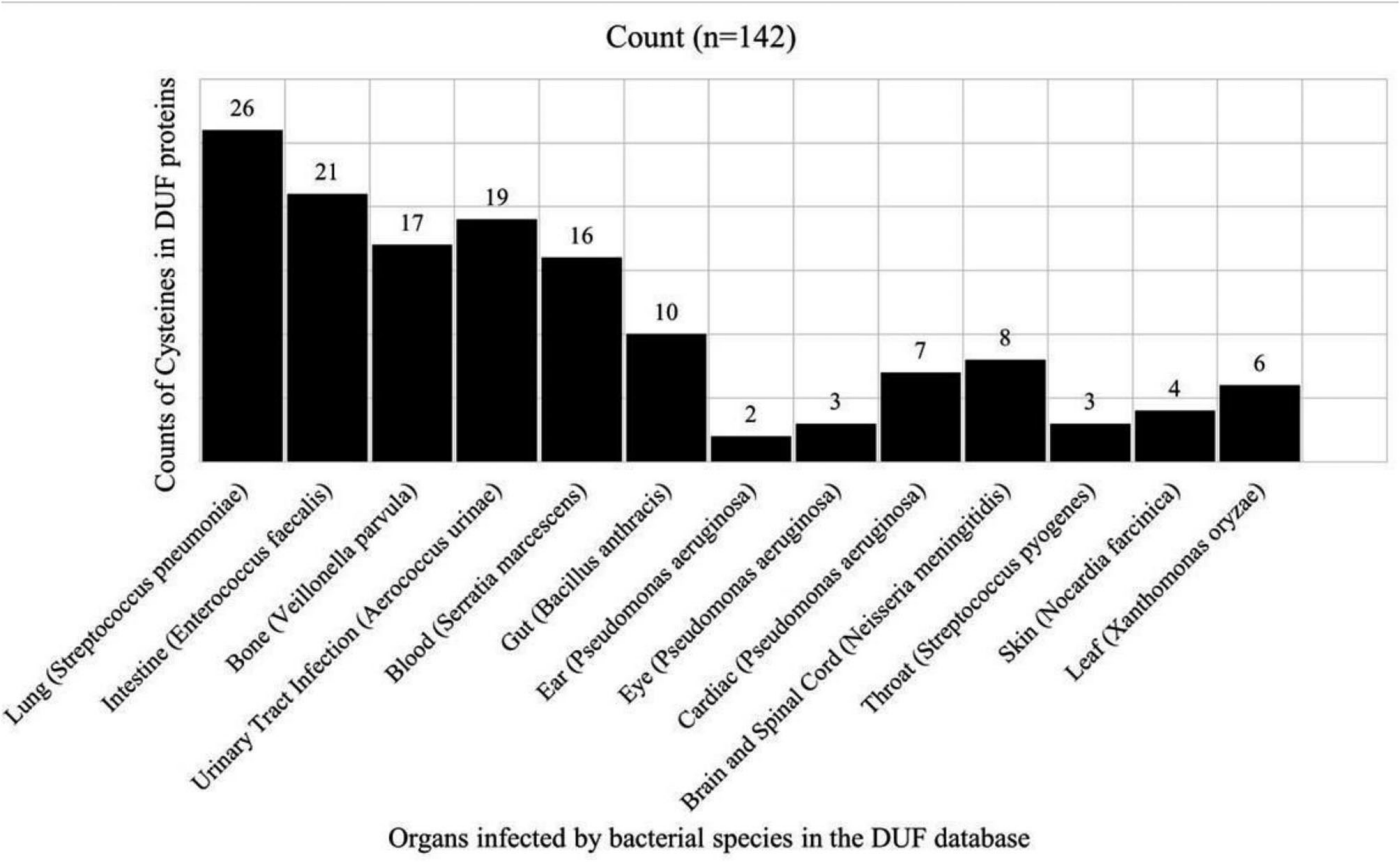
Disease-causing bacteria infecting different organs, categories based on anatomy. Counts of cysteine residues present in DUF proteins per disease category are shown.

One hundred and forty-two cysteine residues were present in the DUF proteins belonging to disease-causing bacterial species. Those 142 cysteine residues were classified into thirteen bacterial infections, categorized based on anatomy (organs) (Fig. 9).

**Figure 10:**
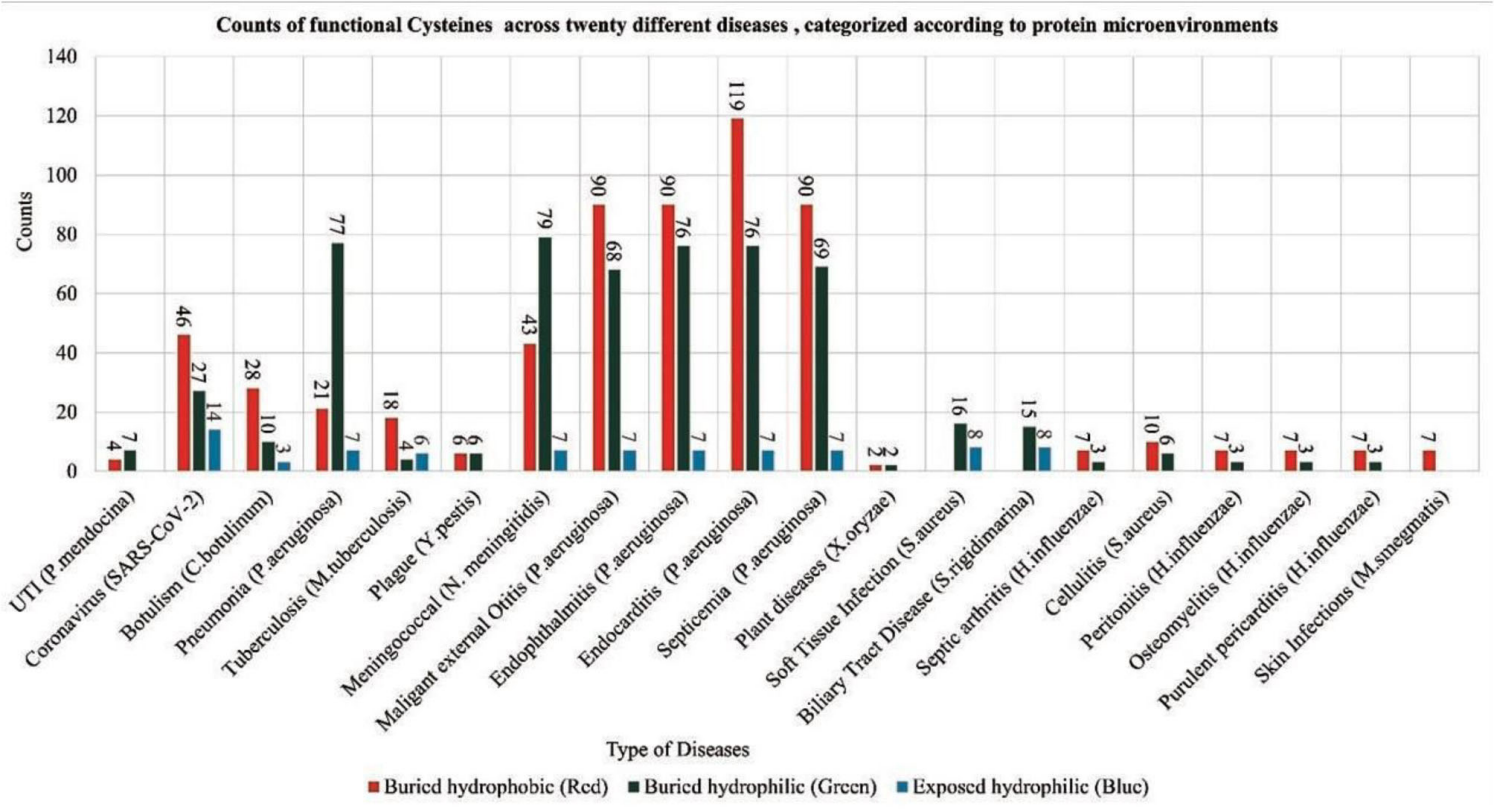
Counts of functional cysteines across twenty different diseases, categorized according to protein microenvironment clusters, (a) DeepCys-structure-based prediction tool and (b) CysDuF database.

The functional cysteine residues are often governed by the local microenvironment, solvent exposure etc. Hence, characterizing cysteine local microenvironments from the viral, bacterial, or other pathogen proteins would provide enhanced understanding on role of the Cys-PTMs in those pathogen-induced diseases. Here, we characterized the functional cysteines from various diseases based on protein microenvironments (Fig. 10).

Protein microenvironment-based classification of functional cysteines from pathogenic species revealed disease preference towards cysteine microenvironments (Table 7). Functional cysteines in several diseases, such as skin infection, septic arthritis, etc, showed higher preferences towards buried hydrophobic clusters. Functional cysteines from certain diseases, like soft tissue infections caused by *S. aureus*, or biliary tract disease, exclude buried hydrophobic microenvironments. Functional cysteines from disease-causing viruses and bacteria, namely, Coronavirus, *Clostridium botulinum, Mycobacterium tuberculosis, Shewanella frigidimarina, mostly prefer buried hydrophilic and exposed hydrophilic microenvironments* (Table S5).

**Table 7:** Normalized cysteine populations in different diseases across microenvironment clusters. The cysteine population within the cluster was normalized by the number of cysteines per disease.

Diseases type (pathogen, if applicable)	Buried hydrophobic	Buried hydrophilic	Exposed hydrophilic
Skin infections	1.00		
Septic arthritis	0.70	0.30	
Peritonitis	0.70	0.30	
Osteomyelitis	0.70	0.30	
Purulent pericarditis	0.70	0.30	
Cellulitis	0.62	0.37	
Plague	0.50	0.50	
Plant diseases	0.50	0.50	
UTI	0.36	0.63	
Coronavirus	0.52	0.31	0.16
Botulism (*Clostridium botulinum*)	0.68	0.24	0.07
Pneumoniae (*Streptococcus pneumoniae*)	0.20	0.73	0.06
Tuberculosis (*Mycobacterium tuberculosis*)	0.64	0.14	0.21
Meningococcal disease (*Neisseria meningitidis*)	0.33	0.61	0.05
Malignant external Otitis (*Pseudomonas aeruginosa*)	0.54	0.41	0.04
Endophthalmitis (*Pseudomonas aeruginosa*)	0.52	0.43	0.04
Endocarditis (*Pseudomonas aeruginosa*)	0.58	0.37	0.03
Septicemia (*Aeromonas hydrophila*)	0.54	0.41	0.04
Soft Tissue Infection (*Staphylococcus aureus*)		0.66	0.33
Biliary tract disease (*Shewanella frigidimarina*)		0.65	0.34

Pathogen names are given along with the diseases for some cases.

The observation of solvent-exposed catalytic cysteine from viruses in DUF proteins was supported by crystal structures: an example, Cys111, catalytic residue from MERS Corona Virus (DUF ID: DUF1175) was exposed on the protein surface and underwent disulfide bond formation with β-mercaptoethanol in the crystal structure (PDB ID: 4R3D); [47]. This cysteine111 in CysDuF database was identified in the exposed-hydrophilic microenvironment, with the predicted S-sulfenylation modification (an oxidized state of the thiol group). The same cysteine residue was reported to undergo ROS-induced oxidative stress, leading to thiol-disulfide imbalance and further oxidation of cysteine, such as sulfenylation [48]. In the DUF protein (DUF: DUF455) from *Mycobacterium tuberculosis* (tuberculosis causing-bacteria), Cys70 formed a zwitter ionic catalytic triad with His110 and Asp127, and the thiolate acted as a nucleophile; thus, the cysteine required hydrophilic microenvironment, concurring with our observation (PDB:4BGF) [49]. The presence of thioether bonds in the ‘exposed hydrophilic’ microenvironment, around cysteines from DUF proteins (DUF: DUF4333) in *Shewanella frigidimarina* causing soft tissue infection and biliary tract diseases was reported in the literature [50] (PDB:1QO8), (PDB:1QJB).

### Comparison of the current database with literature reports

The CysDuF database developed here was compared with fifty databases and prediction servers reported in the literature related to cysteine modifications, motifs, redox properties, regulatory networks, chemoproteomics, evolutionary information, physiochemical properties, etc. (Table S6). Two parameters were compared across the web tools—(a) the number of proteins and (b) the number of cysteine residues in different databases, although each database was designed to manifest different aspects of cysteine properties. There were twenty-two web tools where both features were present. In the remaining databases, either one or zero parameters were present. Many of these web tools were not currently functional. Some of the prediction tools are specific to one Cys_PTM only. For example, the disulfide prediction tool Dipro [51] can predict disulfide bond formation between two sulfydryl groups; however, it is unable to predict the disulfide bond formation probability from a half-cystine (half-cystine is defined as one of the two Sulfydryl groups forming the disulfide bond). Hence, the results of Dipro cannot be compared to those of DeepCys, which predicts the probability of a half-cystine. Similarly, the results from the literature reported metal binding site prediction server, MIB2 [52], are not comparable to those from DeepCys. The metal-binding prediction server MIB2 provides eighteen different types of metal ion binding site predictions. However, the DeepCys tool can only predict cysteine metal-binding probability in general. As DeepCys can predict only disulfide, metal-ion, and thioether from the current CysDUF database, DeepCys efficacy cannot be compared for CysDUF database with the existing prediction tools. To note, thioether is predicted for the first time, using DeepCys prediction server.

### Web application

#### DeepCys web application

**Figure 11:**
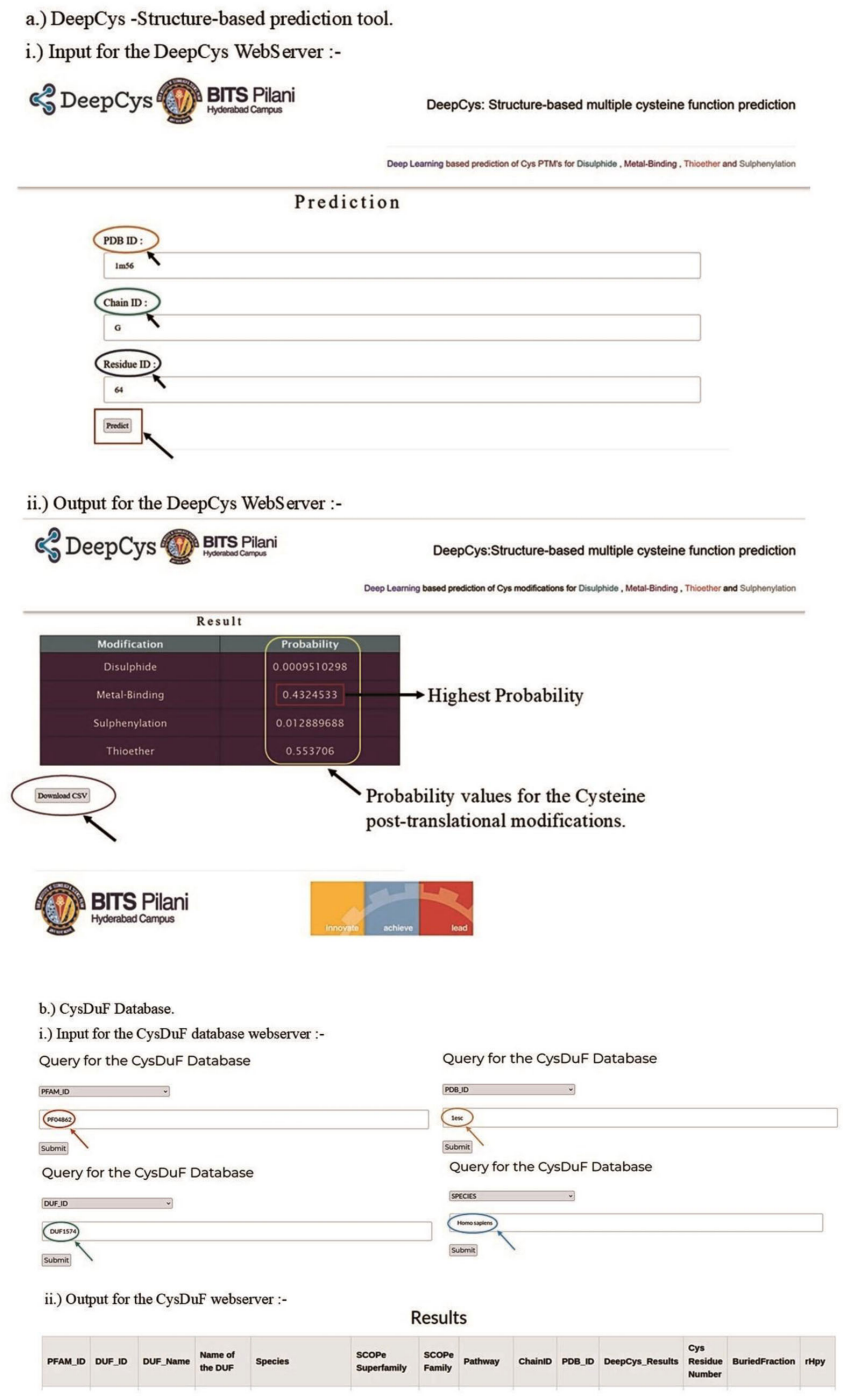
Web application for (a) DeepCys—structure-based prediction tool and (b) CysDUF database.

A user-friendly web application DeepCys (https:/deepcys.bits-hyderabad.ac.in) was built using the Flask web framework. The input, output, and work flow of the web application are shown (Fig. 11a). The web application is deployed using the NGINX and http reverse proxy server. The structure-based prediction tool can be accessed by clicking the prediction button on the navigation bar. The web application has a form that requests three inputs corresponding to a cysteine, namely, (a) PDB ID of the protein, (b) Chain ID, and (c) residue of the Cys. Based on these inputs additional parameters were internally computed to predict four probability values and the most probable cysteine modifications.

#### DUF database web application

A user-friendly web application DUF Database (https://cysduf.bits-hyderabad.ac.in/) was built using the Flask web framework. The flowchart for input, output, and the internal storage of information used in this web application is shown (Fig. 11b). The web application is deployed using the NGINX and HTTP reverse proxy server. The DUF database application has a form that requests any one of three inputs—PDB ID, DUF ID, or PFAM ID. The results are downloadable in multiple formats, CSV, text, or JSON.

## Conclusions

With the advent of high-throughput structure prediction methods, a large number of protein structures, including DUF proteins, were experimentally solved, which required functional characterization. The rigor, expense, and time required for experimental characterization, could be reduced by computational approaches. Aim of this study was to characterize and annotate the functions of catalytic cysteine in DUF proteins, using computational methods. Annotation and characterization of functional cysteine in DUF proteins were performed on seven biochemical processes, namely, ETC, glutathione metabolism, Fe–S-cluster biogenesis, fatty acid synthesis, photosynthesis, Kreb’s cycle, and pentose phosphate pathway. Cysteine post-translation modifications were predicted using DeepCys software, and the results were validated with the experimental findings reported in the PDB header files. Structure-based protein microenvironment computation was done using software developed earlier. The DeepCys tool can currently predict only four Cys-PTMs, ignoring other important modifications. This limitation can be addressed by upgrading the Cys-PTM prediction model based on protein sequences, where larger datasets are available, to be reported elsewhere. The sequence, structure, microenvironment, disease, biochemical pathways related to the DUF proteins, and their functional cysteines were consolidated in a database, CysDUF. This database was the first of its kind that stores and retrieves cysteine functional annotations in DUF proteins and elucidated on seven different pathways. The detailed elucidation of cysteine protein microenvironments in all the DUF proteins revealed that, in general, cysteine residues were embedded in buried hydrophobic microenvironments. However, in certain viral proteins, functional cysteine residues were embedded in the exposed and hydrophilic microenvironments. This secondary database would serve as a reference guide to the functional cysteines of DUF proteins and related information. There is a scope for improvement in the cysteine function prediction, as the current method predicts only four cysteine PTMs, this was due to the limited availability of PDB crystal structure data while training the deep neural network. The prediction method could be complemented using the sequence-based method, albeit, less accurate compared to the structure-based method, where sufficient data is available for a larger number of cysteine PTMs to train deep neural network models. Prediction of a larger number of cysteine modifications would add further significance to the database.

## Supplementary Material

baag002_Supplemental_File

## Data Availability

The data and software are available in the following GitHub link: https://github.com/devhimd19/CysDUF.
