# Supplementary material for: CysDuF database: annotation and characterization of cysteine residues in domain of unknown function proteins based on cysteine post-translational modifications, their protein microenvironments, biochemical pathways, taxonomy, and diseases
Source: Database (Oxford). 2026 Jan 23;2026:baag002. doi: 10.1093/database/baag002 (PMC12828279; doi:10.1093/database/baag002)
Supplement: baag002_Supplemental_File [file baag002_supplemental_file.docx]

Supplementary Material

**Accuracy = (TP + TN)/ (TP + TN + FP + FN) Eq 1**

Accuracy is the proportion of the true positive and false negative instances predicted correctly by the classifier with respect to all the data points.

**Precision = TP/ (TP + FP) Eq 2**

Precision is the proportion of the true positive instances predicted correctly by the classifier, with respect to all the data points.

**Recall = TP/(TP+FN) Eq 3**

Recall is the proportion of the true positive instances predicted correctly out of the true positive and false negative data points. In other words, the ability of a classifier to retrieve all appropriate examples is called recall (also known as sensitivity).

**F1 Score = 2*(Recall * Precision) / (Recall + Precision) Eq 4**

F1 scores include both precision and recall into their calculation, they are often regarded as inferior to accuracy metrics. It ranges from 0.0 to 1.0. To compare classifier models, it is recommended to utilise the weighted average of F1 rather than overall accuracy.

**Weighted average F1-score =**$\sum_{i=1}^{5} \mathbf{F1-score*(no. of instances))/5}$ **Eq 5**


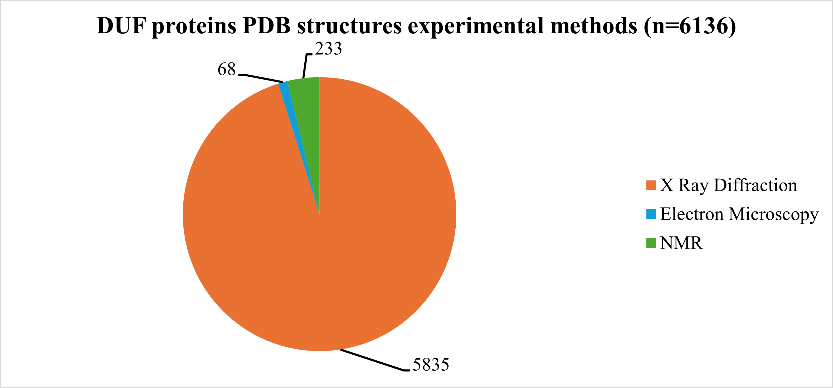


Figure S1: Data Statistics of PDB Structures in the DUF Database

Figure S2: Simple trees for a) eukaryote and b) bacteria

a)


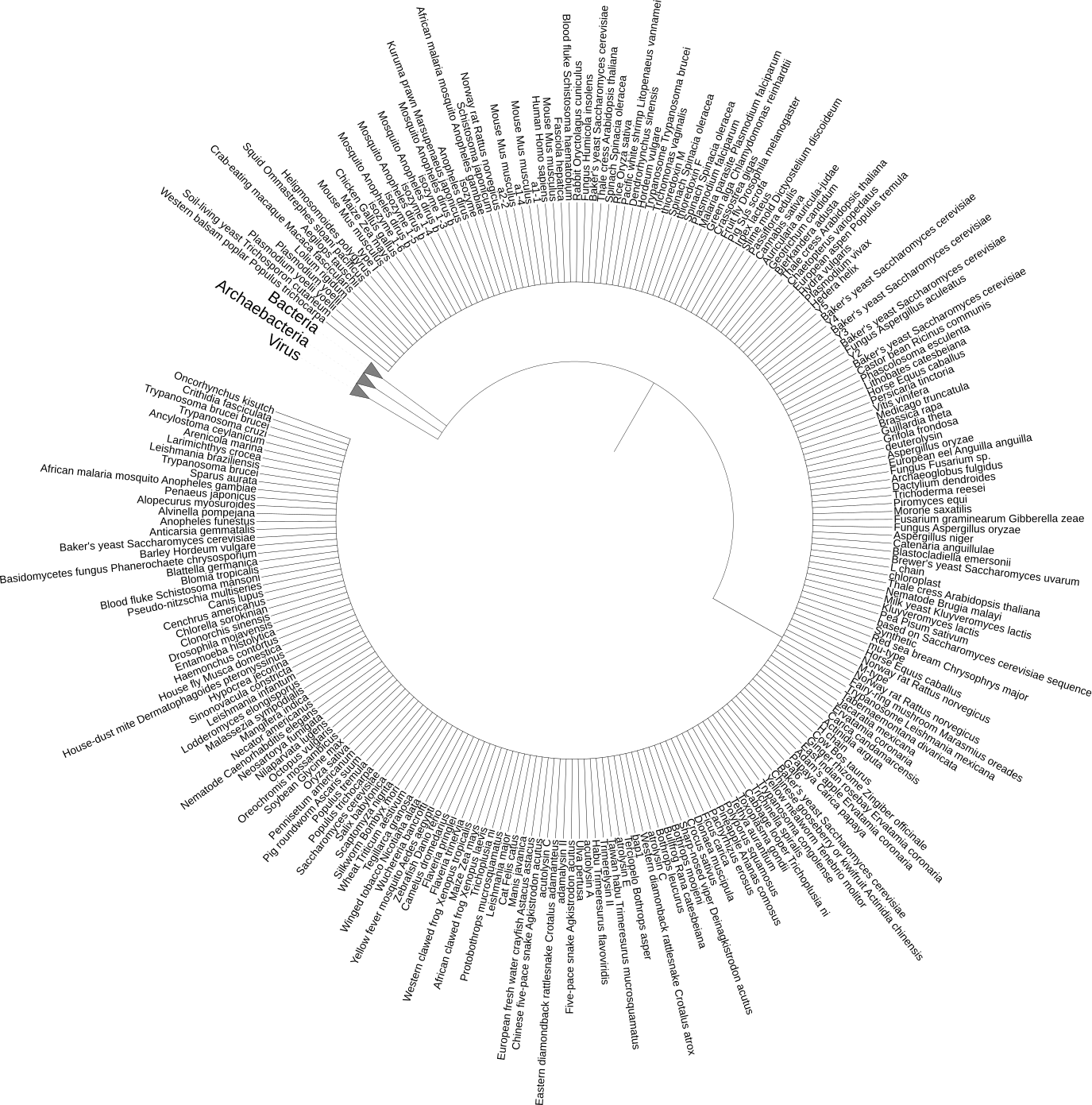


b)


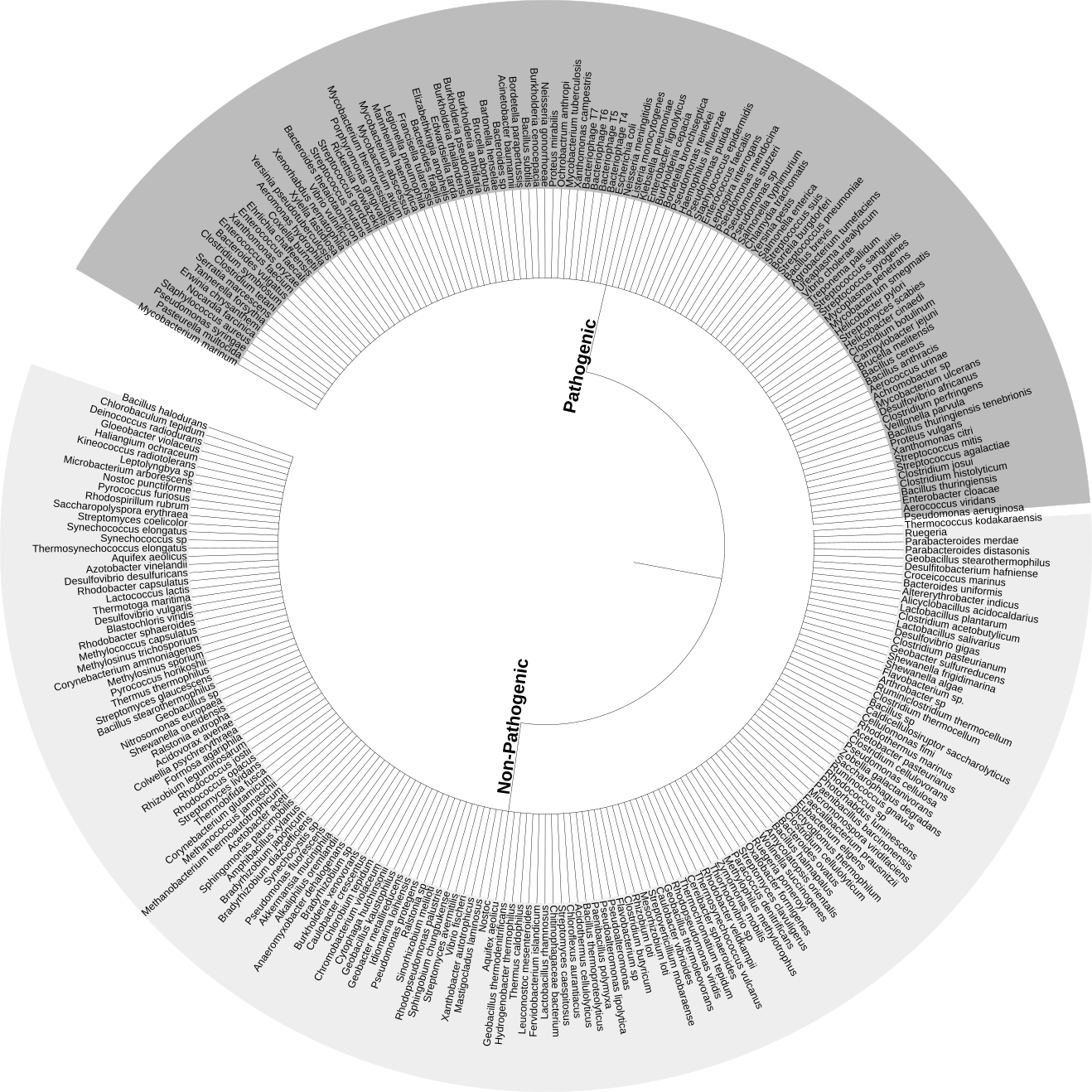


Table S1: List of PDB IDs from X-ray, NMR and electron microscopy

1esc.pdb, 1esd.pdb, 1ese.pdb, 1flc.pdb, 1bwp.pdb, 1bwq.pdb, 1bwr.pdb, 1es9.pdb, 1fxw.pdb, 1wab.pdb, 1vyh.pdb, 2hsj.pdb, 3dt6.pdb, 3dt8.pdb, 3dt9.pdb, 1deo.pdb, 1dex.pdb, 1k7c.pdb, 1pp4.pdb, 3c1u.pdb, 1yzf.pdb, 1ivn.pdb, 1j00.pdb, 1jrl.pdb, 1u8u.pdb, 1v2g.pdb, 5tic.pdb, 5tid.pdb, 5tie.pdb, 5tif.pdb, 6lfb.pdb, 6lfc.pdb, 1vjg.pdb, 1z8h.pdb, 1zmb.pdb, 2aea.pdb, 2apj.pdb, 2o14.pdb, 3bzw.pdb, 3dc7.pdb, 3rjt.pdb, 7c2a.pdb, 7c2c.pdb, 7c2d.pdb, 7c82.pdb, 7c84.pdb, 7c85.pdb, 4ppy.pdb, 4hf7.pdb, 4iyj.pdb, 6njc.pdb, 6xpg.pdb, 6xpm.pdb, 7c23.pdb, 7c29.pdb, 4rsh.pdb, 3w7v.pdb, 4jhl.pdb, 4jj4.pdb, 4jj6.pdb, 4jko.pdb, 4oao.pdb, 4oap.pdb, 5bn1.pdb, 3p94.pdb, 4q9a.pdb, 3hp4.pdb, 4jgg.pdb, 5jd3.pdb, 4rw0.pdb, 5zur.pdb, 6ebo.pdb, 6ebp.pdb, 6ebz.pdb, 6qo5.pdb, 6qo9.pdb, 6qob.pdb, 6tqx.pdb, 6tqy.pdb, 6tqz.pdb, 1s3q.pdb, 5ls9.pdb, 5v5k.pdb, 1sq3.pdb, 6qo7.pdb, 6qo8.pdb, 6tqv.pdb, 6tqw.pdb, 4bmo.pdb, 4bmp.pdb, 4bmq.pdb, 4bmr.pdb, 4bmt.pdb, 4bmu.pdb, 2rcc.pdb, 2chp.pdb, 4dr0.pdb, 3fvb.pdb, 3kwo.pdb, 5wpn.pdb, 4cmy.pdb, 6zjk.pdb, 6lij.pdb, 2f7n.pdb, 2c2f.pdb, 2c2u.pdb, 7emk.pdb, 5uwz.pdb, 5n5f.pdb, 5lbh.pdb, 5u1a.pdb, 5u1b.pdb, 2vux.pdb, 4zjk.pdb, 5jkk.pdb, 5jkl.pdb, 5jkm.pdb, 5n26.pdb, 5n27.pdb, 5xb1.pdb, 5yi5.pdb, 7ck8.pdb, 7ck9.pdb, 7k3v.pdb, 7k3w.pdb, 4a25.pdb, 6a4u.pdb, 7dqp.pdb, 7ek4.pdb, 7ek5.pdb, 7ek7.pdb, 6lkp.pdb, 2yjj.pdb, 2yjk.pdb, 3wnw.pdb, 7a4m.pdb, 2z90.pdb, 3b3h.pdb, 3bkn.pdb, 4m32.pdb, 4m33.pdb, 4m34.pdb, 4m35.pdb, 5ww3.pdb, 5ww4.pdb, 5ww5.pdb, 5ww6.pdb, 5ww7.pdb, 5ww8.pdb, 5ww9.pdb, 3qd8.pdb, 3uof.pdb, 3uoi.pdb, 3oj5.pdb, 2wtl.pdb, 3qb9.pdb, 7o6e.pdb, 7die.pdb, 5hjf.pdb, 5hjh.pdb, 5i4j.pdb, 6lk9.pdb, 6kh0.pdb, 6kh3.pdb, 6kh4.pdb, 6kh5.pdb, 6lbc.pdb, 6lbd.pdb, 6lru.pdb, 6lrv.pdb, 6lrw.pdb, 6lrx.pdb, 6ls2.pdb, 7cpc.pdb, 7cpi.pdb, 7dqo.pdb, 4zkh.pdb, 4zkw.pdb, 4zkx.pdb, 4zl5.pdb, 4zl6.pdb, 4zlw.pdb, 4zmc.pdb, 3r2h.pdb, 3r2k.pdb, 3r2l.pdb, 3r2m.pdb, 3r2o.pdb, 3r2r.pdb, 3r2s.pdb, 4to9.pdb, 4toa.pdb, 4tod.pdb, 4toe.pdb, 4tof.pdb, 4tog.pdb, 4toh.pdb, 5d8o.pdb, 5d8p.pdb, 6nlg.pdb, 2jd6.pdb, 2jd7.pdb, 2jd8.pdb, 2x17.pdb, 5da5.pdb, 5l89.pdb, 5l8b.pdb, 5l8g.pdb, 6sv1.pdb, 5l8f.pdb, 6suw.pdb, 6y2n.pdb, 6lp5.pdb, 3a68.pdb, 3a9q.pdb, 6j4j.pdb, 6j4m.pdb, 2d5k.pdb, 2wla.pdb, 2wlu.pdb, 2xgw.pdb, 4n83.pdb, 5xx9.pdb, 4cya.pdb, 4cyb.pdb, 2vxx.pdb, 5ouw.pdb, 5ouz.pdb, 6gka.pdb, 6gkb.pdb, 6gkc.pdb, 6som.pdb, 6son.pdb, 6soo.pdb, 6sop.pdb, 6soq.pdb, 6sor.pdb, 7pfi.pdb, 6kzy.pdb, 6l55.pdb, 6l56.pdb, 2c41.pdb, 2fjc.pdb, 1z6o.pdb, 3vnx.pdb, 3iq1.pdb, 7aik.pdb, 7vp8.pdb, 1bg7.pdb, 1mfr.pdb, 1rcc.pdb, 1rcd.pdb, 1rce.pdb, 1rcg.pdb, 1rci.pdb, 3rgd.pdb, 4lpj.pdb, 4lq7.pdb, 4lqh.pdb, 4lqj.pdb, 4lqv.pdb, 4lyu.pdb, 4lyx.pdb, 4my7.pdb, 5xhi.pdb, 5xhm.pdb, 5xhn.pdb, 5xho.pdb, 6i36.pdb, 1aew.pdb, 1dat.pdb, 1gwg.pdb, 1hrs.pdb, 1ier.pdb, 1ies.pdb, 1xz1.pdb, 1xz3.pdb, 2g4h.pdb, 2gyd.pdb, 2v2i.pdb, 2v2j.pdb, 2v2n.pdb, 2v2o.pdb, 2v2s.pdb, 2w0o.pdb, 2z5p.pdb, 2z5q.pdb, 2z5r.pdb, 2za6.pdb, 2za7.pdb, 2zg7.pdb, 2zg8.pdb, 2zg9.pdb, 2zur.pdb, 3af7.pdb, 3af8.pdb, 3af9.pdb, 3f32.pdb, 3f33.pdb, 3f34.pdb, 2ffx.pdb, 2fg4.pdb, 2fg8.pdb, 3hx2.pdb, 3hx5.pdb, 3hx7.pdb, 3kxu.pdb, 5lg8.pdb, 6wx6.pdb, 1fha.pdb, 2fha.pdb, 3ajo.pdb, 3ajp.pdb, 3ajq.pdb, 4dyx.pdb, 4dyy.pdb, 4dyz.pdb, 4dz0.pdb, 4oyn.pdb, 4y08.pdb, 4ykh.pdb, 5cmq.pdb, 5gn8.pdb, 5gou.pdb, 5up7.pdb, 5up8.pdb, 5up9.pdb, 5vtd.pdb, 5znd.pdb, 6b8f.pdb, 6b8g.pdb, 6ftv.pdb, 6h6t.pdb, 6h6u.pdb, 6ipo.pdb, 6ipp.pdb, 6ipq.pdb, 6job.pdb, 6ke2.pdb, 6ke4.pdb, 6m52.pdb, 6m54.pdb, 6wyf.pdb, 6wyg.pdb, 6wyh.pdb, 6z6u.pdb, 6z9e.pdb, 6z9f.pdb, 7a6a.pdb, 7a6b.pdb, 7jgk.pdb, 7jgo.pdb, 7jgq.pdb, 7k26.pdb, 7r5o.pdb, 1r03.pdb, 1h96.pdb, 1lb3.pdb, 1sof.pdb, 1nf4.pdb, 1nf6.pdb, 1nfv.pdb, 3e1j.pdb, 3e2c.pdb, 3ghq.pdb, 4xks.pdb, 4xkt.pdb, 4xku.pdb, 5xgo.pdb, 6p8k.pdb, 6p8l.pdb, 4u3g.pdb, 1bcf.pdb, 1bfr.pdb, 2htn.pdb, 2vxi.pdb, 2y3q.pdb, 3e1l.pdb, 3e1m.pdb, 3e1n.pdb, 3e1o.pdb, 3e1p.pdb, 3e1q.pdb, 1jgc.pdb, 1o9r.pdb, 1ji5.pdb, 1jig.pdb, 1n1q.pdb, 6gcm.pdb, 6qvx.pdb, 1dps.pdb, 1f30.pdb, 1f33.pdb, 1jre.pdb, 1jts.pdb, 1l8h.pdb, 1l8i.pdb, 6zgl.pdb, 1moj.pdb, 1tjo.pdb, 1tk6.pdb, 1tko.pdb, 1tkp.pdb, 1ji4.pdb, 3t9j.pdb, 3ta8.pdb, 4evb.pdb, 4evc.pdb, 4evd.pdb, 4eve.pdb, 1zuj.pdb, 1zs3.pdb, 1qgh.pdb, 2bjy.pdb, 2bk6.pdb, 2bkc.pdb, 6hui.pdb, 6hv1.pdb, 6sev.pdb, 6hvq.pdb, 2pyb.pdb, 1vei.pdb, 1vel.pdb, 1veq.pdb, 2yw6.pdb, 2yw7.pdb, 1umn.pdb, 2bw1.pdb, 2ux1.pdb, 2v15.pdb, 2xjm.pdb, 2xjn.pdb, 2xjo.pdb, 2xkq.pdb, 2fzf.pdb, 1vjx.pdb, 1j30.pdb, 1yux.pdb, 1yuz.pdb, 1yv1.pdb, 1krq.pdb, 4reu.pdb, 4xgs.pdb, 1eum.pdb, 3e6r.pdb, 3e6s.pdb, 4ism.pdb, 4isp.pdb, 4itt.pdb, 4itw.pdb, 4iwj.pdb, 4iwk.pdb, 4ixk.pdb, 1vlg.pdb, 7dy8.pdb, 7dy9.pdb, 7dya.pdb, 7dyb.pdb, 1b71.pdb, 1dvb.pdb, 1jyb.pdb, 1lkm.pdb, 1lko.pdb, 1lkp.pdb, 1qyb.pdb, 1ryt.pdb, 1s2z.pdb, 1s30.pdb, 1nnq.pdb, 2hr5.pdb, 3kx9.pdb, 2fkz.pdb, 2fl0.pdb, 4am2.pdb, 4am4.pdb, 4am5.pdb, 3ge4.pdb, 3ka3.pdb, 3ka4.pdb, 3ka6.pdb, 3ka8.pdb, 3ka9.pdb, 3rbc.pdb, 3re7.pdb, 3se1.pdb, 3sh6.pdb, 3shx.pdb, 4das.pdb, 4lpm.pdb, 4lpn.pdb, 4mjy.pdb, 4mku.pdb, 4ml5.pdb, 4mn9.pdb, 4p18.pdb, 4di0.pdb, 6ljg.pdb, 7dlb.pdb, 4ztt.pdb, 4cvp.pdb, 4cvr.pdb, 4cvs.pdb, 4cvt.pdb, 7aqs.pdb, 6b0d.pdb, 3bve.pdb, 3bvf.pdb, 3bvi.pdb, 3bvk.pdb, 3bvl.pdb, 3egm.pdb, 5c6f.pdb, 2v2l.pdb, 2v2m.pdb, 2v2p.pdb, 2v2r.pdb, 2za8.pdb, 3np2.pdb, 2cei.pdb, 2chi.pdb, 2cih.pdb, 2clu.pdb, 2cn6.pdb, 2cn7.pdb, 2iu2.pdb, 2z6m.pdb, 5z8j.pdb, 5z8s.pdb, 5z8u.pdb, 5z91.pdb, 7o63.pdb, 2iy4.pdb, 5j8s.pdb, 5j8w.pdb, 5j93.pdb, 5j9v.pdb, 5jac.pdb, 6i9p.pdb, 6i9t.pdb, 6iaf.pdb, 6iaj.pdb, 5oba.pdb, 1uvh.pdb, 5h46.pdb, 6lpd.pdb, 6lpe.pdb, 3is7.pdb, 3is8.pdb, 3ise.pdb, 3isf.pdb, 4e6k.pdb, 4tob.pdb, 4toc.pdb, 5d8q.pdb, 5d8r.pdb, 5d8s.pdb, 5d8x.pdb, 5d8y.pdb, 6nlf.pdb, 6nli.pdb, 6nlj.pdb, 6nlk.pdb, 6nll.pdb, 6nlm.pdb, 6nln.pdb, 7k5e.pdb, 7k5f.pdb, 7k5g.pdb, 7k5h.pdb, 3mps.pdb, 3pwf.pdb, 3pza.pdb, 3qvd.pdb, 3gvy.pdb, 3ak8.pdb, 3ak9.pdb, 2cf7.pdb, 4cy9.pdb, 6ls0.pdb, 6ls3.pdb, 1z4a.pdb, 6txh.pdb, 6txi.pdb, 6txj.pdb, 6txk.pdb, 6txl.pdb, 6txm.pdb, 6txn.pdb, 3qz3.pdb, 4dyu.pdb, 7ppt.pdb, 1afr.pdb, 1oq4.pdb, 1oq7.pdb, 1oq9.pdb, 1oqb.pdb, 1fyz.pdb, 1fz0.pdb, 1fz1.pdb, 1fz2.pdb, 1fz3.pdb, 1fz4.pdb, 1fz5.pdb, 1fz6.pdb, 1fz7.pdb, 1fz8.pdb, 1fz9.pdb, 1fzh.pdb, 1fzi.pdb, 1mmo.pdb, 1mty.pdb, 1xmf.pdb, 1xmg.pdb, 1xmh.pdb, 1xu3.pdb, 1xu5.pdb, 1xvb.pdb, 1xvc.pdb, 1xvd.pdb, 1xve.pdb, 1xvf.pdb, 1xvg.pdb, 1mhy.pdb, 1mhz.pdb, 4gam.pdb, 1otk.pdb, 1za0.pdb, 1jk0.pdb, 1smq.pdb, 1sms.pdb, 1syy.pdb, 1kgn.pdb, 1kgo.pdb, 1kgp.pdb, 1oqu.pdb, 3dhz.pdb, 3mjo.pdb, 1av8.pdb, 1biq.pdb, 1jpr.pdb, 1jqc.pdb, 1mrr.pdb, 1mxr.pdb, 1pfr.pdb, 1pim.pdb, 1piu.pdb, 1piy.pdb, 1piz.pdb, 1pj0.pdb, 1pj1.pdb, 1pm2.pdb, 1r65.pdb, 1rib.pdb, 1rnr.pdb, 1rsr.pdb, 1rsv.pdb, 1xik.pdb, 1yfd.pdb, 2alx.pdb, 2av8.pdb, 2xof.pdb, 5ci0.pdb, 5ci1.pdb, 5ci4.pdb, 2uw2.pdb, 1h0n.pdb, 1h0o.pdb, 1w68.pdb, 1w69.pdb, 1xsm.pdb, 1uzr.pdb, 1r2f.pdb, 2bq1.pdb, 2r2f.pdb, 3n1x.pdb, 3n1y.pdb, 3n1z.pdb, 3n20.pdb, 3rn9.pdb, 3rna.pdb, 3rnb.pdb, 3rnc.pdb, 3rne.pdb, 3rnf.pdb, 3rng.pdb, 1t0q.pdb, 1t0r.pdb, 1t0s.pdb, 2inc.pdb, 2ind.pdb, 2rdb.pdb, 2j2f.pdb, 2xz0.pdb, 4v0j.pdb, 2ani.pdb, 4d8f.pdb, 4d8g.pdb, 4m1h.pdb, 4m1i.pdb, 3n37.pdb, 3n38.pdb, 3n39.pdb, 3n3a.pdb, 3n3b.pdb, 4m1f.pdb, 3pvr.pdb, 3pvt.pdb, 3pvy.pdb, 3pw1.pdb, 3pw8.pdb, 3pwq.pdb, 4ii4.pdb, 5ci2.pdb, 5ci3.pdb, 2uw1.pdb, 2iyh.pdb, 3hf1.pdb, 3olj.pdb, 3vpm.pdb, 3vpn.pdb, 3vpo.pdb, 4djn.pdb, 6d7k.pdb, 6vk4.pdb, 6vk5.pdb, 6vk6.pdb, 6vk7.pdb, 6vk8.pdb, 6yd0.pdb, 6ydi.pdb, 6ydu.pdb, 6yy3.pdb, 7m8q.pdb, 7m8r.pdb, 7s6r.pdb, 2o1z.pdb, 3dhg.pdb, 3dhh.pdb, 3dhi.pdb, 3ge3.pdb, 3ge8.pdb, 3i5j.pdb, 3i63.pdb, 3q14.pdb, 3q2a.pdb, 3q3m.pdb, 3q3n.pdb, 3q3o.pdb, 3ri7.pdb, 3rmk.pdb, 4p1b.pdb, 4p1c.pdb, 5tds.pdb, 5tdt.pdb, 5tdu.pdb, 5tdv.pdb, 1t3g.pdb, 2js7.pdb, 2z5v.pdb, 4dom.pdb, 4eo7.pdb, 7beq.pdb, 7ber.pdb, 7l6w.pdb, 7szl.pdb, 4w8g.pdb, 2rd3.pdb, 3ibx.pdb, 2q32.pdb, 2qpp.pdb, 2rgz.pdb, 4wmh.pdb, 5uc8.pdb, 5uc9.pdb, 5uca.pdb, 5kzl.pdb, 2gm7.pdb, 2gm8.pdb, 3mvu.pdb, 4fn6.pdb, 3no6.pdb, 1z72.pdb, 2qzc.pdb, 1mli.pdb, 1i1g.pdb, 1ri7.pdb, 2e1c.pdb, 2zny.pdb, 2znz.pdb, 2cg4.pdb, 2cfx.pdb, 2cyy.pdb, 2e7w.pdb, 1lq9.pdb, 1n5q.pdb, 1n5s.pdb, 1n5t.pdb, 1n5v.pdb, 1si9.pdb, 1tr0.pdb, 1nwj.pdb, 1q4r.pdb, 1q83.pdb, 2q3p.pdb, 1rjj.pdb, 1tz0.pdb, 1xbw.pdb, 1sqe.pdb, 2zdp.pdb, 3lgm.pdb, 3lgn.pdb, 3qgp.pdb, 4fnh.pdb, 4fni.pdb, 1iuj.pdb, 2go8.pdb, 2zdo.pdb, 1q8b.pdb, 1mwq.pdb, 1tuw.pdb, 1s7i.pdb, 1vdh.pdb, 1t0t.pdb, 6vsa.pdb, 5t2k.pdb, 2omo.pdb, 2pd1.pdb, 1x7v.pdb, 1y0h.pdb, 1r6y.pdb, 1tuv.pdb, 1wd6.pdb, 2asy.pdb, 2hiq.pdb, 1vqs.pdb, 2ap6.pdb, 1vqy.pdb, 3mm1.pdb, 3mm2.pdb, 3mm3.pdb, 3vxi.pdb, 3vxj.pdb, 2d3q.pdb, 2gvk.pdb, 2hag.pdb, 2iiz.pdb, 3afv.pdb, 2ftr.pdb, 2fiu.pdb, 2cb2.pdb, 2yav.pdb, 2yaw.pdb, 2yax.pdb, 6qjc.pdb, 6qka.pdb, 6qkm.pdb, 6qmv.pdb, 6qne.pdb, 6qo0.pdb, 3bxv.pdb, 6m35.pdb, 6m3x.pdb, 2okq.pdb, 2ifx.pdb, 3hds.pdb, 3hf5.pdb, 3hfk.pdb, 2od4.pdb, 2od6.pdb, 2op5.pdb, 2pgc.pdb, 1x8d.pdb, 7byu.pdb, 7byw.pdb, 4au9.pdb, 4uzi.pdb, 4w7j.pdb, 4w7k.pdb, 4w7l.pdb, 4w7m.pdb, 4w7n.pdb, 4w7o.pdb, 5ag0.pdb, 5ag1.pdb, 5ikd.pdb, 5ikg.pdb, 3fmb.pdb, 2qyc.pdb, 4lbh.pdb, 4lbi.pdb, 4lbp.pdb, 5b08.pdb, 5b09.pdb, 5b0a.pdb, 5b0b.pdb, 5b0c.pdb, 5b0d.pdb, 5b0e.pdb, 5b0f.pdb, 5b0g.pdb, 3lo3.pdb, 5vj0.pdb, 3qmq.pdb, 5gt2.pdb, 6hhn.pdb, 6fiy.pdb, 6fks.pdb, 6fkt.pdb, 6fl2.pdb, 6rpd.pdb, 6rpe.pdb, 6rqy.pdb, 6rr1.pdb, 6rr4.pdb, 6rr5.pdb, 6rr6.pdb, 6rr8.pdb, 5loq.pdb, 4wws.pdb, 6fxj.pdb, 6fxq.pdb, 4dpo.pdb, 3hx9.pdb, 4nl5.pdb, 5uq4.pdb, 6ds7.pdb, 6ds8.pdb, 6ple.pdb, 2p5v.pdb, 2p6s.pdb, 2p6t.pdb, 5xzq.pdb, 5xzt.pdb, 5y02.pdb, 2qlw.pdb, 2qlx.pdb, 3qnr.pdb, 3qns.pdb, 3vec.pdb, 3ved.pdb, 3vee.pdb, 3vef.pdb, 3veg.pdb, 4hov.pdb, 3znj.pdb, 3znu.pdb, 2bbe.pdb, 7o9j.pdb, 7o9l.pdb, 7odz.pdb, 4gu7.pdb, 6yr4.pdb, 6yrc.pdb, 6yrd.pdb, 6yrj.pdb, 2e7x.pdb, 2efp.pdb, 2efn.pdb, 2efo.pdb, 2efq.pdb, 2pmh.pdb, 2pn6.pdb, 2yx4.pdb, 2yx7.pdb, 3bgu.pdb, 5de0.pdb, 2gff.pdb, 7d8m.pdb, 1zma.pdb, 1nm3.pdb, 1aaz.pdb, 1aba.pdb, 1de1.pdb, 1de2.pdb, 1ego.pdb, 1egr.pdb, 1grx.pdb, 1qfn.pdb, 1fov.pdb, 3grx.pdb, 1b4q.pdb, 1jhb.pdb, 1kte.pdb, 1r7h.pdb, 4fiw.pdb, 1h75.pdb, 1z6n.pdb, 1ttz.pdb, 1xpv.pdb, 1fo5.pdb, 1nho.pdb, 1ilo.pdb, 1se1.pdb, 1vrs.pdb, 1uc7.pdb, 2fwe.pdb, 2fwf.pdb, 2fwg.pdb, 2fwh.pdb, 1nsw.pdb, 1nw2.pdb, 1quw.pdb, 1rqm.pdb, 3dyr.pdb, 4x43.pdb, 6yev.pdb, 1f6m.pdb, 1keb.pdb, 1oaz.pdb, 1skr.pdb, 1sks.pdb, 1skw.pdb, 1sl0.pdb, 1sl1.pdb, 1sl2.pdb, 1srx.pdb, 1t7p.pdb, 1t8e.pdb, 1tho.pdb, 1tk0.pdb, 1tk5.pdb, 1tk8.pdb, 1tkd.pdb, 1txx.pdb, 1x9m.pdb, 1x9s.pdb, 1x9w.pdb, 1xoa.pdb, 1xob.pdb, 1zcp.pdb, 1zyq.pdb, 1zzy.pdb, 2ajq.pdb, 2bto.pdb, 2eio.pdb, 2eiq.pdb, 2fch.pdb, 2fd3.pdb, 2h6x.pdb, 2h6y.pdb, 2h6z.pdb, 2h70.pdb, 2h71.pdb, 2h72.pdb, 2h73.pdb, 2h74.pdb, 2h75.pdb, 2h76.pdb, 2o8v.pdb, 2tir.pdb, 2trx.pdb, 4hu7.pdb, 4hu9.pdb, 5e4w.pdb, 5hr1.pdb, 6h1y.pdb, 6h7j.pdb, 6h7l.pdb, 6h7m.pdb, 6h7n.pdb, 6h7o.pdb, 1ti3.pdb, 1xw9.pdb, 1xwa.pdb, 1xwb.pdb, 1xwc.pdb, 1dby.pdb, 1ep7.pdb, 1ep8.pdb, 1tof.pdb, 1aiu.pdb, 1auc.pdb, 1cqg.pdb, 1cqh.pdb, 1ert.pdb, 1eru.pdb, 1erv.pdb, 1erw.pdb, 1m7t.pdb, 1mdi.pdb, 1mdj.pdb, 1mdk.pdb, 1trs.pdb, 1tru.pdb, 1trv.pdb, 1trw.pdb, 2hsh.pdb, 2hxk.pdb, 2ifq.pdb, 2iiy.pdb, 3e3e.pdb, 3m9j.pdb, 3m9k.pdb, 3trx.pdb, 4ll1.pdb, 4ll4.pdb, 4oo4.pdb, 4oo5.pdb, 4trx.pdb, 5dqy.pdb, 1syr.pdb, 1thx.pdb, 2mmn.pdb, 2mmo.pdb, 4j56.pdb, 4j57.pdb, 1f9m.pdb, 1faa.pdb, 1fb6.pdb, 1fb0.pdb, 1gl8.pdb, 2o7k.pdb, 2o85.pdb, 2o87.pdb, 2o89.pdb, 3die.pdb, 1xfl.pdb, 2f51.pdb, 1r26.pdb, 1wik.pdb, 1sen.pdb, 1gh2.pdb, 1wjk.pdb, 2i4a.pdb, 3lef.pdb, 2gzy.pdb, 2gzz.pdb, 2ipa.pdb, 4hua.pdb, 4ip1.pdb, 4ip6.pdb, 2eir.pdb, 5hr0.pdb, 5hr2.pdb, 5hr3.pdb, 2vm1.pdb, 2vm2.pdb, 2k8v.pdb, 2yan.pdb, 3kd0.pdb, 3qfa.pdb, 3qfb.pdb, 3zyw.pdb, 4rqr.pdb, 4hs1.pdb, 4k8m.pdb, 4f2i.pdb, 3zzx.pdb, 4aj6.pdb, 4aj7.pdb, 4aj8.pdb, 4v2l.pdb, 4v2m.pdb, 4v2n.pdb, 5g2z.pdb, 5g30.pdb, 5g31.pdb, 1wmj.pdb, 2pu9.pdb, 2puk.pdb, 7c2b.pdb, 1hyu.pdb, 1zyn.pdb, 1zyp.pdb, 2b5e.pdb, 2djj.pdb, 2djk.pdb, 2kp2.pdb, 1bjx.pdb, 1mek.pdb, 2bjx.pdb, 1a8l.pdb, 1j08.pdb, 1xtx.pdb, 2kp1.pdb, 2rue.pdb, 2ruf.pdb, 1a8y.pdb, 3us3.pdb, 3v1w.pdb, 1k0m.pdb, 1k0n.pdb, 1k0o.pdb, 1rk4.pdb, 1oe7.pdb, 1oe8.pdb, 2c80.pdb, 2ca8.pdb, 1fhe.pdb, 2fhe.pdb, 1ags.pdb, 1gsd.pdb, 1gse.pdb, 1gsf.pdb, 1guh.pdb, 1gul.pdb, 1gum.pdb, 1k3l.pdb, 1k3o.pdb, 1k3y.pdb, 1pkw.pdb, 1pkz.pdb, 1pl1.pdb, 1pl2.pdb, 1usb.pdb, 1ydk.pdb, 2r3x.pdb, 2r6k.pdb, 3i69.pdb, 3i6a.pdb, 3ik7.pdb, 3ik9.pdb, 3ktl.pdb, 3l0h.pdb, 3q74.pdb, 3u6v.pdb, 4hj2.pdb, 5jcu.pdb, 5lcz.pdb, 5ld0.pdb, 6ato.pdb, 6atp.pdb, 6atq.pdb, 6atr.pdb, 6yaw.pdb, 1f3a.pdb, 1f3b.pdb, 1b48.pdb, 1guk.pdb, 1ml6.pdb, 1ev4.pdb, 1ev9.pdb, 1bg5.pdb, 1dug.pdb, 1gne.pdb, 1gta.pdb, 1gtb.pdb, 1m99.pdb, 1m9a.pdb, 1m9b.pdb, 1u87.pdb, 1u88.pdb, 3crt.pdb, 3cru.pdb, 5gzz.pdb, 6rwd.pdb, 1a0f.pdb, 1b8x.pdb, 1n2a.pdb, 2nto.pdb, 2pvq.pdb, 1pmt.pdb, 2pmt.pdb, 1f2e.pdb, 1pn9.pdb, 3f63.pdb, 3g7i.pdb, 1jlv.pdb, 1jlw.pdb, 1r5a.pdb, 1v2a.pdb, 1c72.pdb, 1gsu.pdb, 1gtu.pdb, 1hna.pdb, 1hnb.pdb, 1hnc.pdb, 1xw5.pdb, 1xw6.pdb, 1xwk.pdb, 1yj6.pdb, 1ykc.pdb, 2ab6.pdb, 2f3m.pdb, 2gtu.pdb, 3gtu.pdb, 3gur.pdb, 4gtu.pdb, 5hwl.pdb, 7beu.pdb, 1b4p.pdb, 1gsb.pdb, 1gsc.pdb, 1mtc.pdb, 2gst.pdb, 3fyg.pdb, 3gst.pdb, 4gst.pdb, 5fwg.pdb, 5gst.pdb, 6gst.pdb, 6gsu.pdb, 6gsv.pdb, 6gsw.pdb, 6gsx.pdb, 6gsy.pdb, 1eem.pdb, 3lf1.pdb, 3vln.pdb, 4is0.pdb, 4yqm.pdb, 4yqu.pdb, 4yqv.pdb, 5ueh.pdb, 5v3q.pdb, 5yvn.pdb, 5yvo.pdb, 6mhb.pdb, 6mhc.pdb, 6mhd.pdb, 6pnm.pdb, 6pnn.pdb, 6pno.pdb, 1axd.pdb, 1bye.pdb, 1aw9.pdb, 1bx9.pdb, 1gnw.pdb, 10gs.pdb, 11gs.pdb, 12gs.pdb, 13gs.pdb, 14gs.pdb, 16gs.pdb, 17gs.pdb, 18gs.pdb, 19gs.pdb, 1aqv.pdb, 1aqw.pdb, 1aqx.pdb, 1eog.pdb, 1eoh.pdb, 1gss.pdb, 1kbn.pdb, 1lbk.pdb, 1md3.pdb, 1md4.pdb, 1pgt.pdb, 1px6.pdb, 1px7.pdb, 1zgn.pdb, 20gs.pdb, 21gs.pdb, 22gs.pdb, 2a2r.pdb, 2a2s.pdb, 2gss.pdb, 2pgt.pdb, 3csh.pdb, 3csi.pdb, 3csj.pdb, 3dd3.pdb, 3dgq.pdb, 3gss.pdb, 3gus.pdb, 3hjm.pdb, 3hjo.pdb, 3hkr.pdb, 3ie3.pdb, 3n9j.pdb, 3pgt.pdb, 4gss.pdb, 4pgt.pdb, 5dak.pdb, 5dal.pdb, 5dcg.pdb, 5ddl.pdb, 5djl.pdb, 5djm.pdb, 5gss.pdb, 5j41.pdb, 5jcw.pdb, 5l6x.pdb, 5x79.pdb, 6ap9.pdb, 6gss.pdb, 6llx.pdb, 6y1e.pdb, 7bia.pdb, 7gss.pdb, 8gss.pdb, 9gss.pdb, 1bay.pdb, 1glp.pdb, 1glq.pdb, 1gsy.pdb, 1gti.pdb, 2glr.pdb, 2oa7.pdb, 2oac.pdb, 3o76.pdb, 1tu7.pdb, 1tu8.pdb, 2gsr.pdb, 1m0u.pdb, 1tw9.pdb, 1iyh.pdb, 1iyi.pdb, 1v40.pdb, 2cvd.pdb, 3ee2.pdb, 3vi5.pdb, 3vi7.pdb, 5ywe.pdb, 5ywx.pdb, 5yx1.pdb, 6n4e.pdb, 6w58.pdb, 6w8h.pdb, 6ztc.pdb, 7jr6.pdb, 7jr8.pdb, 1pd2.pdb, 5y9z.pdb, 6n69.pdb, 1gsq.pdb, 2gsq.pdb, 1gwc.pdb, 1oyj.pdb, 1ljr.pdb, 2ljr.pdb, 3ljr.pdb, 1fw1.pdb, 1e6b.pdb, 1g7o.pdb, 7d9l.pdb, 7dkp.pdb, 7dkr.pdb, 1nhy.pdb, 1zgm.pdb, 2fno.pdb, 1z9h.pdb, 2pbj.pdb, 1okt.pdb, 1pa3.pdb, 1q4j.pdb, 3fr9.pdb, 3frc.pdb, 4zxg.pdb, 1g6w.pdb, 1g6y.pdb, 1hqo.pdb, 1jzr.pdb, 1k0a.pdb, 1k0b.pdb, 1k0c.pdb, 1k0d.pdb, 2c4j.pdb, 6zb6.pdb, 2aaw.pdb, 3fr3.pdb, 3fr6.pdb, 5a4u.pdb, 5a4v.pdb, 5a4w.pdb, 5a5k.pdb, 1a0r.pdb, 1b9x.pdb, 1b9y.pdb, 2trc.pdb, 1g7e.pdb, 1ovn.pdb, 2c0e.pdb, 2c0f.pdb, 2c0g.pdb, 2c1y.pdb, 1pqn.pdb, 1qgv.pdb, 1syx.pdb, 4bwq.pdb, 4bws.pdb, 2av4.pdb, 1eej.pdb, 1g0t.pdb, 1jzd.pdb, 1jzo.pdb, 1tjd.pdb, 1t3b.pdb, 1v57.pdb, 1v58.pdb, 2h0g.pdb, 2h0h.pdb, 2h0i.pdb, 5g1k.pdb, 5g1l.pdb, 4i5q.pdb, 4ilf.pdb, 4npb.pdb, 1prx.pdb, 5b6m.pdb, 5b6n.pdb, 1xiy.pdb, 4d73.pdb, 1xcc.pdb, 3tb2.pdb, 1weo.pdb, 2bmx.pdb, 4ma9.pdb, 4mab.pdb, 4xra.pdb, 4xrd.pdb, 4xs1.pdb, 4xs4.pdb, 4xts.pdb, 1kyg.pdb, 1n8j.pdb, 1yep.pdb, 1yex.pdb, 1yf0.pdb, 1yf1.pdb, 5uka.pdb, 2cx3.pdb, 2cx4.pdb, 4gqc.pdb, 4gqf.pdb, 1gp1.pdb, 2f8a.pdb, 3ios.pdb, 1jfu.pdb, 2h30.pdb, 2fy6.pdb, 2jzr.pdb, 2jzs.pdb, 2k9f.pdb, 1x0r.pdb, 2cv4.pdb, 1h4o.pdb, 1hd2.pdb, 1oc3.pdb, 1urm.pdb, 1zye.pdb, 1foh.pdb, 1pn0.pdb, 1tp9.pdb, 1psq.pdb, 2cvb.pdb, 2ywo.pdb, 1xvw.pdb, 1xxu.pdb, 1lu4.pdb, 3hvs.pdb, 3hvv.pdb, 3hvx.pdb, 3i43.pdb, 1qxh.pdb, 1q98.pdb, 1xvq.pdb, 1y25.pdb, 1st9.pdb, 1su9.pdb, 2f9s.pdb, 2h19.pdb, 2h1a.pdb, 2h1b.pdb, 2h1d.pdb, 2h1g.pdb, 2b5x.pdb, 1qmv.pdb, 5ijt.pdb, 7kiz.pdb, 7kj0.pdb, 7kj1.pdb, 1qq2.pdb, 2h01.pdb, 1zof.pdb, 1kng.pdb, 1z5y.pdb, 1on4.pdb, 1xzo.pdb, 2b7j.pdb, 2b7k.pdb, 1wp0.pdb, 2ggt.pdb, 1ewx.pdb, 1ezk.pdb, 1o7u.pdb, 1o85.pdb, 1o8w.pdb, 1o8x.pdb, 1okd.pdb, 1qk8.pdb, 1o73.pdb, 1fg4.pdb, 1i5g.pdb, 1o6j.pdb, 1o81.pdb, 1oc8.pdb, 1oc9.pdb, 1e2y.pdb, 4llr.pdb, 1vgs.pdb, 2e2g.pdb, 2e2m.pdb, 2nvl.pdb, 3a2v.pdb, 3a2w.pdb, 3a2x.pdb, 3a5w.pdb, 5xbs.pdb, 6krk.pdb, 6krm.pdb, 6krp.pdb, 6krq.pdb, 6krr.pdb, 6krs.pdb, 7c87.pdb, 7c89.pdb, 7c8a.pdb, 7cqj.pdb, 2zct.pdb, 1we0.pdb, 4fh8.pdb, 4kw6.pdb, 2v2g.pdb, 2v32.pdb, 2v41.pdb, 2wfc.pdb, 2b5y.pdb, 3c71.pdb, 3c73.pdb, 3sbc.pdb, 5dvb.pdb, 4txo.pdb, 4je1.pdb, 2b1k.pdb, 2b1l.pdb, 2g0f.pdb, 4af2.pdb, 3k8n.pdb, 2rii.pdb, 2vl2.pdb, 2vl3.pdb, 2vl9.pdb, 3hy2.pdb, 3mng.pdb, 3tjb.pdb, 3tjf.pdb, 3tjg.pdb, 3tjj.pdb, 3tjk.pdb, 3tkp.pdb, 3tkq.pdb, 3tkr.pdb, 3tks.pdb, 4xcs.pdb, 5hqp.pdb, 5jcg.pdb, 5ucx.pdb, 7lj1.pdb, 3qpm.pdb, 4kb3.pdb, 4kce.pdb, 3s9f.pdb, 3tue.pdb, 4k1f.pdb, 2z9s.pdb, 5zte.pdb, 6gxg.pdb, 6gxy.pdb, 1uul.pdb, 7e4u.pdb, 4wet.pdb, 4wey.pdb, 4wf4.pdb, 4wf5.pdb, 1a23.pdb, 1a24.pdb, 1a2j.pdb, 1a2l.pdb, 1a2m.pdb, 1ac1.pdb, 1acv.pdb, 1bq7.pdb, 1dsb.pdb, 1fvj.pdb, 1fvk.pdb, 1ti1.pdb, 1u3a.pdb, 1un2.pdb, 2hi7.pdb, 2ndo.pdb, 4zij.pdb, 5qkc.pdb, 5qkd.pdb, 5qke.pdb, 5qkf.pdb, 5qkg.pdb, 5qkh.pdb, 5qki.pdb, 5qkj.pdb, 5qkk.pdb, 5qkl.pdb, 5qkm.pdb, 5qkn.pdb, 5qko.pdb, 5qkp.pdb, 5qkq.pdb, 5qkr.pdb, 5qks.pdb, 5qkt.pdb, 5qku.pdb, 5qkv.pdb, 5qkw.pdb, 5qkx.pdb, 5qky.pdb, 5qkz.pdb, 5ql0.pdb, 5ql1.pdb, 5ql2.pdb, 5ql3.pdb, 5ql4.pdb, 5ql5.pdb, 5ql6.pdb, 5ql7.pdb, 5ql8.pdb, 5ql9.pdb, 5qla.pdb, 5qlb.pdb, 5qlc.pdb, 5qld.pdb, 5qle.pdb, 5qlf.pdb, 5qlg.pdb, 5qlh.pdb, 5qli.pdb, 5qlj.pdb, 5qlk.pdb, 5qll.pdb, 5qlm.pdb, 5qln.pdb, 5qlo.pdb, 5qlp.pdb, 5qlq.pdb, 5qlr.pdb, 5qls.pdb, 5qlt.pdb, 5qlu.pdb, 5qlv.pdb, 5qlw.pdb, 5qlx.pdb, 5qly.pdb, 5qlz.pdb, 5qm0.pdb, 5qm1.pdb, 5qm2.pdb, 5qm3.pdb, 5qm4.pdb, 5qm5.pdb, 5qm6.pdb, 5qm7.pdb, 5qm8.pdb, 5qm9.pdb, 5qma.pdb, 5qmb.pdb, 5qmc.pdb, 5qmd.pdb, 5qme.pdb, 5qmf.pdb, 5qmg.pdb, 5qmh.pdb, 5qmi.pdb, 5qmj.pdb, 5qmk.pdb, 5qml.pdb, 5qmm.pdb, 5qmn.pdb, 5qmo.pdb, 5qmp.pdb, 5qmq.pdb, 5qmr.pdb, 5qms.pdb, 5qmt.pdb, 5qmu.pdb, 5qmv.pdb, 5qmw.pdb, 5qmx.pdb, 5qmy.pdb, 5qmz.pdb, 5qn0.pdb, 5qn1.pdb, 5qn2.pdb, 5qn3.pdb, 5qn4.pdb, 5qn5.pdb, 5qn6.pdb, 5qn7.pdb, 5qn8.pdb, 5qn9.pdb, 5qna.pdb, 5qnb.pdb, 5qnc.pdb, 5qnd.pdb, 5qne.pdb, 5qnf.pdb, 5qng.pdb, 5qnh.pdb, 5qni.pdb, 5qnj.pdb, 5qnk.pdb, 5qnl.pdb, 5qnm.pdb, 5qnn.pdb, 5qno.pdb, 5qnp.pdb, 5qnq.pdb, 5qnr.pdb, 5qns.pdb, 5qnt.pdb, 5qnu.pdb, 5qnv.pdb, 5qnw.pdb, 5qnx.pdb, 5qny.pdb, 5qnz.pdb, 5qo0.pdb, 5qo1.pdb, 5qo2.pdb, 5qo3.pdb, 5qo4.pdb, 5qo5.pdb, 5qo6.pdb, 5qo7.pdb, 5qo8.pdb, 5qo9.pdb, 5qoa.pdb, 5qob.pdb, 5qoc.pdb, 5qod.pdb, 5qoe.pdb, 5qof.pdb, 5qog.pdb, 6bqx.pdb, 6br4.pdb, 6pbi.pdb, 6pc9.pdb, 6pd7.pdb, 6pdh.pdb, 6pg1.pdb, 6pg2.pdb, 6pgj.pdb, 6piq.pdb, 6pli.pdb, 6pmf.pdb, 6pml.pdb, 6poh.pdb, 6poi.pdb, 6poq.pdb, 6pvy.pdb, 6pvz.pdb, 6whd.pdb, 3dks.pdb, 1bed.pdb, 1z6m.pdb, 1r4w.pdb, 2b3s.pdb, 2b6m.pdb, 1yzx.pdb, 3rpn.pdb, 3rpp.pdb, 2mbs.pdb, 4mcu.pdb, 4oce.pdb, 4ocf.pdb, 4od7.pdb, 3l9s.pdb, 4dvc.pdb, 7lui.pdb, 2ijy.pdb, 1j0f.pdb, 1t1v.pdb, 1sj6.pdb, 1t4y.pdb, 1t4z.pdb, 1r5p.pdb, 4kso.pdb, 2qke.pdb, 1wwj.pdb, 1vgl.pdb, 5jwo.pdb, 5jwr.pdb, 5jyt.pdb, 5jyv.pdb, 1wou.pdb, 1v9w.pdb, 1xg8.pdb, 1wpi.pdb, 2axo.pdb, 2hfd.pdb, 2gzp.pdb, 2qgv.pdb, 2fug.pdb, 3i9v.pdb, 3iam.pdb, 3ias.pdb, 6y11.pdb, 1s3a.pdb, 2fa8.pdb, 2a2p.pdb, 2a4h.pdb, 2ojl.pdb, 2oka.pdb, 2obk.pdb, 2p0g.pdb, 2dlx.pdb, 4p3y.pdb, 2hls.pdb, 2il3.pdb, 2imi.pdb, 2imk.pdb, 4gsn.pdb, 6khx.pdb, 4tr0.pdb, 4tr1.pdb, 7c10.pdb, 7c12.pdb, 7c13.pdb, 6riv.pdb, 2xhf.pdb, 4knd.pdb, 4igj.pdb, 4kae.pdb, 4kdy.pdb, 3f6d.pdb, 3g7j.pdb, 3zmk.pdb, 3zml.pdb, 4hde.pdb, 4nmu.pdb, 5dbq.pdb, 2ywm.pdb, 2yzh.pdb, 5ovq.pdb, 2ayt.pdb, 3ic4.pdb, 4xhm.pdb, 5abr.pdb, 3zij.pdb, 3zit.pdb, 2jsy.pdb, 2jsz.pdb, 2voc.pdb, 3erw.pdb, 3eur.pdb, 3ewl.pdb, 3gkx.pdb, 3hxs.pdb, 3hyp.pdb, 2lrn.pdb, 2ls5.pdb, 2kuc.pdb, 3fw2.pdb, 2l5l.pdb, 2lja.pdb, 4grf.pdb, 4k9z.pdb, 4ka0.pdb, 2fa4.pdb, 2hsy.pdb, 2oe0.pdb, 2oe1.pdb, 2oe3.pdb, 3cmi.pdb, 3ctf.pdb, 3ctg.pdb, 3d4m.pdb, 3d5j.pdb, 3d6i.pdb, 3pin.pdb, 6utl.pdb, 2m80.pdb, 3l4n.pdb, 4dss.pdb, 5j3r.pdb, 5ykj.pdb, 5ykw.pdb, 7bvv.pdb, 2iwt.pdb, 2klx.pdb, 4f0b.pdb, 4f0c.pdb, 4zb6.pdb, 4zb7.pdb, 4zb8.pdb, 4zb9.pdb, 4zba.pdb, 4zbb.pdb, 4zbd.pdb, 5o00.pdb, 4q5r.pdb, 4q5n.pdb, 2c8u.pdb, 2cai.pdb, 2caq.pdb, 2f8f.pdb, 1u3i.pdb, 2v1m.pdb, 2wgr.pdb, 2xbi.pdb, 2xbq.pdb, 2xc2.pdb, 3zl5.pdb, 3ztl.pdb, 3zvj.pdb, 3hd5.pdb, 4wbj.pdb, 4wbr.pdb, 4mf7.pdb, 4nhz.pdb, 2kok.pdb, 2mzc.pdb, 2khp.pdb, 6ckp.pdb, 4iel.pdb, 5enu.pdb, 4f82.pdb, 4qq7.pdb, 4mf5.pdb, 4mf6.pdb, 4k2d.pdb, 6v91.pdb, 2dsa.pdb, 2gdr.pdb, 1sji.pdb, 4j2f.pdb, 6esx.pdb, 5ev0.pdb, 1vf1.pdb, 1vf2.pdb, 1vf3.pdb, 1vf4.pdb, 4i2t.pdb, 4i2u.pdb, 3or5.pdb, 3gl3.pdb, 3keb.pdb, 3iso.pdb, 4l5l.pdb, 4l5o.pdb, 6gn9.pdb, 6gnd.pdb, 4mh2.pdb, 4mh3.pdb, 5jy5.pdb, 3hcz.pdb, 3gnj.pdb, 6zom.pdb, 4hi7.pdb, 2hze.pdb, 2hzf.pdb, 5zf2.pdb, 5zpv.pdb, 6udg.pdb, 4cw9.pdb, 5y63.pdb, 3gx0.pdb, 5hfk.pdb, 1yka.pdb, 2wci.pdb, 3c7m.pdb, 4ksm.pdb, 4kx4.pdb, 2vim.pdb, 2wb9.pdb, 2wdu.pdb, 2wrt.pdb, 3lgc.pdb, 3msz.pdb, 6weg.pdb, 5u51.pdb, 5u56.pdb, 3ein.pdb, 3f6f.pdb, 3gh6.pdb, 3mak.pdb, 4pnf.pdb, 4png.pdb, 4yh2.pdb, 5f0g.pdb, 6t2t.pdb, 6zmu.pdb, 7dax.pdb, 7day.pdb, 7daz.pdb, 7db0.pdb, 7db1.pdb, 7db2.pdb, 7db3.pdb, 7db4.pdb, 2ywi.pdb, 3hdc.pdb, 3kcm.pdb, 5xft.pdb, 6i19.pdb, 6i1c.pdb, 6q46.pdb, 6q47.pdb, 6q6t.pdb, 6q6u.pdb, 6q6v.pdb, 7ncv.pdb, 7ncw.pdb, 2ws2.pdb, 3lyk.pdb, 6kil.pdb, 6bkv.pdb, 2vlt.pdb, 2vlu.pdb, 2vlv.pdb, 6zj9.pdb, 6zjc.pdb, 3vwx.pdb, 5zwp.pdb, 4q5q.pdb, 1tdi.pdb, 1u6t.pdb, 1w4v.pdb, 1w89.pdb, 1wry.pdb, 1x5c.pdb, 1x5d.pdb, 1x5e.pdb, 1xbs.pdb, 1xwg.pdb, 2ahe.pdb, 2c3n.pdb, 2c3q.pdb, 2c3t.pdb, 2cq9.pdb, 2ct6.pdb, 2d2z.pdb, 2diz.pdb, 2dj0.pdb, 2fls.pdb, 2gqk.pdb, 2gql.pdb, 2gqm.pdb, 2gs3.pdb, 2gt5.pdb, 2gt6.pdb, 2gvp.pdb, 2he3.pdb, 2hrf.pdb, 2hrn.pdb, 2ht9.pdb, 2i3y.pdb, 2j9h.pdb, 2lns.pdb, 2lnt.pdb, 2obi.pdb, 2p31.pdb, 2per.pdb, 2pn8.pdb, 2r37.pdb, 2r4v.pdb, 2r5g.pdb, 2rli.pdb, 2vcq.pdb, 2vcr.pdb, 2vct.pdb, 2vcv.pdb, 2vcw.pdb, 2vcx.pdb, 2vcz.pdb, 2vd0.pdb, 2vd1.pdb, 2wju.pdb, 2wz9.pdb, 3cyn.pdb, 3gix.pdb, 3h8q.pdb, 3idv.pdb, 3kij.pdb, 3km6.pdb, 3kmn.pdb, 3kmo.pdb, 3kxo.pdb, 3ma4.pdb, 3o3t.pdb, 3p8w.pdb, 3p90.pdb, 3ph9.pdb, 3q18.pdb, 3q19.pdb, 3qag.pdb, 3qr6.pdb, 3swl.pdb, 3tgz.pdb, 3uj1.pdb, 3uvh.pdb, 3uvt.pdb, 3vww.pdb, 3wgd.pdb, 3wge.pdb, 3zfb.pdb, 3zfl.pdb, 4acs.pdb, 4ec0.pdb, 4edy.pdb, 4edz.pdb, 4ee0.pdb, 4ef0.pdb, 4gwr.pdb, 4in0.pdb, 4iqa.pdb, 4jzq.pdb, 4k0g.pdb, 4k0n.pdb, 4k7i.pdb, 4k7n.pdb, 4k7o.pdb, 4mmm.pdb, 4mpf.pdb, 4mpg.pdb, 4pok.pdb, 4pol.pdb, 4pom.pdb, 5ais.pdb, 5aiv.pdb, 5aix.pdb, 5b8a.pdb, 5b8b.pdb, 6elw.pdb, 6hkq.pdb, 6hn3.pdb, 6owv.pdb, 6y2h.pdb, 6vpd.pdb, 6dxn.pdb, 3niv.pdb, 4jrr.pdb, 4kun.pdb, 6e0g.pdb, 4ivf.pdb, 2j23.pdb, 5g5e.pdb, 5g5f.pdb, 5kej.pdb, 4iq1.pdb, 4iw9.pdb, 3hz4.pdb, 3uap.pdb, 3uar.pdb, 2cz2.pdb, 2cz3.pdb, 2dc5.pdb, 2dj1.pdb, 2dj2.pdb, 2dml.pdb, 2lv3.pdb, 2oad.pdb, 3aps.pdb, 3vwv.pdb, 5l71.pdb, 6ery.pdb, 6erz.pdb, 4pob.pdb, 4xwt.pdb, 5vo7.pdb, 6ap5.pdb, 2i1u.pdb, 2l4q.pdb, 2lqo.pdb, 2lqq.pdb, 3nof.pdb, 2l59.pdb, 3o6t.pdb, 5epf.pdb, 2on5.pdb, 2on7.pdb, 3w8s.pdb, 4ofm.pdb, 4ofn.pdb, 4oft.pdb, 4hoj.pdb, 3dvw.pdb, 3dvx.pdb, 3hz8.pdb, 2l5o.pdb, 2znm.pdb, 3a3t.pdb, 1yq1.pdb, 1zl9.pdb, 5j9b.pdb, 5j9c.pdb, 3wyw.pdb, 5h5l.pdb, 5b7c.pdb, 2hnl.pdb, 5y7i.pdb, 5d9t.pdb, 5d9v.pdb, 5d9w.pdb, 5d9x.pdb, 5an1.pdb, 5iqy.pdb, 4q5f.pdb, 3cxg.pdb, 2c0d.pdb, 3ul3.pdb, 4hjm.pdb, 4kje.pdb, 4kjf.pdb, 4mzb.pdb, 4mzc.pdb, 4n0z.pdb, 4n10.pdb, 4n11.pdb, 4o32.pdb, 7dik.pdb, 7dim.pdb, 7din.pdb, 7dio.pdb, 7dip.pdb, 7diq.pdb, 7dir.pdb, 7dis.pdb, 7dit.pdb, 7diu.pdb, 7div.pdb, 7diw.pdb, 7diz.pdb, 7dj0.pdb, 2i81.pdb, 4l0u.pdb, 2h66.pdb, 2feg.pdb, 4l0w.pdb, 4evm.pdb, 2e7p.pdb, 3fz9.pdb, 3fza.pdb, 4ri6.pdb, 4ri7.pdb, 5nyk.pdb, 5nyl.pdb, 5nym.pdb, 5nyn.pdb, 5nyo.pdb, 2p5q.pdb, 2p5r.pdb, 3d21.pdb, 3d22.pdb, 2m72.pdb, 6p0w.pdb, 3kh7.pdb, 3kh9.pdb, 6tum.pdb, 4eci.pdb, 4ecj.pdb, 2mbt.pdb, 3h93.pdb, 4zl7.pdb, 4zl8.pdb, 4zl9.pdb, 5dch.pdb, 5tlq.pdb, 3lyp.pdb, 3m3m.pdb, 4ikh.pdb, 3mdk.pdb, 4nax.pdb, 2jl4.pdb, 2v6k.pdb, 3uma.pdb, 3m8n.pdb, 6nup.pdb, 5y4t.pdb, 7dw1.pdb, 7dw2.pdb, 7dw3.pdb, 7dw4.pdb, 7dwd.pdb, 7dwe.pdb, 7dwf.pdb, 7dwg.pdb, 3l9u.pdb, 3l9v.pdb, 4kh7.pdb, 3ir4.pdb, 4i97.pdb, 1y6e.pdb, 3d0z.pdb, 4i8b.pdb, 4wr4.pdb, 4wr5.pdb, 5jly.pdb, 6ji6.pdb, 6n8u.pdb, 4kgi.pdb, 3vfi.pdb, 3ay8.pdb, 3vk9.pdb, 3vpq.pdb, 3vpt.pdb, 3vur.pdb, 3wd6.pdb, 4e8e.pdb, 4e8h.pdb, 5zfg.pdb, 3u5r.pdb, 2vo4.pdb, 3fhs.pdb, 4chs.pdb, 4top.pdb, 5agy.pdb, 6m1t.pdb, 3p7x.pdb, 4ruv.pdb, 2m46.pdb, 5um7.pdb, 3fz4.pdb, 2yp6.pdb, 4hqs.pdb, 4hqz.pdb, 3dex.pdb, 1t00.pdb, 3drn.pdb, 3hhv.pdb, 3hjp.pdb, 3tco.pdb, 2e0q.pdb, 2ywn.pdb, 4g2e.pdb, 3qmx.pdb, 4mja.pdb, 4mjb.pdb, 4mjc.pdb, 4mje.pdb, 3ipz.pdb, 3rhb.pdb, 3rhc.pdb, 5ech.pdb, 5eci.pdb, 5eck.pdb, 5ecl.pdb, 5ecm.pdb, 5ecn.pdb, 5eco.pdb, 5ecp.pdb, 5ecq.pdb, 5ecr.pdb, 5ecs.pdb, 5el8.pdb, 5ela.pdb, 5elg.pdb, 5g5a.pdb, 5lol.pdb, 5o84.pdb, 6ep6.pdb, 6ep7.pdb, 6ezy.pdb, 6f01.pdb, 6f05.pdb, 6g61.pdb, 6g62.pdb, 6lyw.pdb, 6lyx.pdb, 6n5u.pdb, 7bzk.pdb, 7c3f.pdb, 7c65.pdb, 1v98.pdb, 2yzu.pdb, 2lst.pdb, 4tn8.pdb, 2cvk.pdb, 3e0u.pdb, 3h79.pdb, 2ltk.pdb, 2myg.pdb, 2rm5.pdb, 2rm6.pdb, 2vup.pdb, 3dwv.pdb, 3f0i.pdb, 3feu.pdb, 5k1g.pdb, 5k2i.pdb, 5k2j.pdb, 2lku.pdb, 2n5f.pdb, 5ey6.pdb, 5f05.pdb, 5f06.pdb, 5f07.pdb, 5j4u.pdb, 5j5n.pdb, 5mye.pdb, 5n9u.pdb, 7zs3.pdb, 7zzn.pdb, 5zvl.pdb, 6x0b.pdb, 5d73.pdb, 4hz2.pdb, 4l8e.pdb, 2x64.pdb, 2rem.pdb, 3ixr.pdb, 5ft3.pdb, 7ebt.pdb, 3rdw.pdb, 1yy7.pdb, 4g9h.pdb, 4gci.pdb, 2xpd.pdb, 2xpe.pdb, 3zrd.pdb, 3zre.pdb, 2yjh.pdb, 3uiw.pdb, 7dil.pdb, 7pq7.pdb, 7opz.pdb, 1kqf.pdb, 1kqg.pdb, 1q90.pdb, 1vf5.pdb, 2d2c.pdb, 2e75.pdb, 2e76.pdb, 1ezv.pdb, 1kb9.pdb, 1kyo.pdb, 1p84.pdb, 2ibz.pdb, 1bcc.pdb, 2bcc.pdb, 3bcc.pdb, 3cwb.pdb, 3l70.pdb, 3l71.pdb, 3l74.pdb, 3l75.pdb, 1be3.pdb, 1bgy.pdb, 1l0l.pdb, 1l0n.pdb, 1ntk.pdb, 1ntm.pdb, 1ntz.pdb, 1nu1.pdb, 1pp9.pdb, 1ppj.pdb, 1qcr.pdb, 1sqb.pdb, 1sqp.pdb, 1sqv.pdb, 1sqx.pdb, 2a06.pdb, 2fyu.pdb, 5klv.pdb, 6nhg.pdb, 4pd4.pdb, 6t0b.pdb, 6ymx.pdb, 3h1h.pdb, 3h1j.pdb, 3l72.pdb, 3l73.pdb, 1sqq.pdb, 5okd.pdb, 1um3.pdb, 2e74.pdb, 4h13.pdb, 4i7z.pdb, 4pv1.pdb, 2zt9.pdb, 4ogq.pdb, 3cx5.pdb, 3cxh.pdb, 5d8d.pdb, 5dmx.pdb, 6ll9.pdb, 3r5x.pdb, 2yw2.pdb, 2yya.pdb, 3r23.pdb, 4eg0.pdb, 5nrh.pdb, 4egq.pdb, 5nri.pdb, 4egj.pdb, 3ouu.pdb, 3ouz.pdb, 6mel.pdb, 3tqt.pdb, 3lp8.pdb, 4fu0.pdb, 5jvn.pdb, 5jvj.pdb, 5jvl.pdb, 5lu4.pdb, 6mgg.pdb, 6pfn.pdb, 2yrw.pdb, 2yrx.pdb, 2ys6.pdb, 2ys7.pdb, 2dzd.pdb, 4mv1.pdb, 4mv3.pdb, 4mv4.pdb, 4mv6.pdb, 4mv7.pdb, 4mv8.pdb, 4mv9.pdb, 4rzq.pdb, 6oi8.pdb, 6ojh.pdb, 2qk4.pdb, 7kbl.pdb, 7kc7.pdb, 7kct.pdb, 3lwb.pdb, 5mlk.pdb, 5vev.pdb, 2c00.pdb, 2vqd.pdb, 3q1k.pdb, 3i12.pdb, 2i80.pdb, 2vpq.pdb, 3n8d.pdb, 2i87.pdb, 2i8c.pdb, 3k3p.pdb, 2fb9.pdb, 2yzg.pdb, 2yzm.pdb, 2yzn.pdb, 2ip4.pdb, 2zdg.pdb, 2zdh.pdb, 2zdq.pdb, 6u1c.pdb, 6u1d.pdb, 6u1e.pdb, 6u1f.pdb, 6u1g.pdb, 6u1h.pdb, 6u1i.pdb, 6u1j.pdb, 6u1k.pdb, 6dgi.pdb, 3r5f.pdb, 3e5n.pdb, 3rfc.pdb, 4l1k.pdb, 4me6.pdb, 3v4z.pdb, 4zqi.pdb, 5bpf.pdb, 5bph.pdb, 5c1o.pdb, 5c1p.pdb, 1iov.pdb, 1iow.pdb, 2dln.pdb, 1e4e.pdb, 1ehi.pdb, 1glv.pdb, 1gsa.pdb, 1gsh.pdb, 2glt.pdb, 4c5a.pdb, 4c5b.pdb, 4c5c.pdb, 4i4t.pdb, 4i50.pdb, 4i55.pdb, 4ihj.pdb, 4iij.pdb, 4o2a.pdb, 4o2b.pdb, 4o4j.pdb, 4tuy.pdb, 4tv8.pdb, 4tv9.pdb, 4yj2.pdb, 4zhq.pdb, 4zi7.pdb, 4zol.pdb, 5bmv.pdb, 5c8y.pdb, 5ca0.pdb, 5ca1.pdb, 5cb4.pdb, 5ezy.pdb, 5gon.pdb, 5h74.pdb, 5h7o.pdb, 5iyz.pdb, 5j2t.pdb, 5jcb.pdb, 5jh7.pdb, 5jqg.pdb, 5jvd.pdb, 5kx5.pdb, 5la6.pdb, 5lov.pdb, 5lp6.pdb, 5lxs.pdb, 5lxt.pdb, 5lyj.pdb, 5m7e.pdb, 5m7g.pdb, 5m8d.pdb, 5m8g.pdb, 5mf4.pdb, 5nfz.pdb, 5ng1.pdb, 5njh.pdb, 5o7a.pdb, 5osk.pdb, 5ov7.pdb, 5s4l.pdb, 5s4m.pdb, 5s4n.pdb, 5s4o.pdb, 5s4p.pdb, 5s4q.pdb, 5s4r.pdb, 5s4s.pdb, 5s4t.pdb, 5s4u.pdb, 5s4v.pdb, 5s4w.pdb, 5s4x.pdb, 5s4y.pdb, 5s4z.pdb, 5s50.pdb, 5s51.pdb, 5s52.pdb, 5s53.pdb, 5s54.pdb, 5s55.pdb, 5s56.pdb, 5s57.pdb, 5s58.pdb, 5s59.pdb, 5s5a.pdb, 5s5b.pdb, 5s5c.pdb, 5s5d.pdb, 5s5e.pdb, 5s5f.pdb, 5s5g.pdb, 5s5h.pdb, 5s5i.pdb, 5s5j.pdb, 5s5k.pdb, 5s5l.pdb, 5s5m.pdb, 5s5n.pdb, 5s5o.pdb, 5s5p.pdb, 5s5q.pdb, 5s5r.pdb, 5s5s.pdb, 5s5t.pdb, 5s5u.pdb, 5s5v.pdb, 5s5w.pdb, 5s5x.pdb, 5s5y.pdb, 5s5z.pdb, 5s60.pdb, 5s61.pdb, 5s62.pdb, 5s63.pdb, 5s64.pdb, 5s65.pdb, 5s66.pdb, 5s67.pdb, 5sb4.pdb, 5sb9.pdb, 5xaf.pdb, 5xhc.pdb, 5xi5.pdb, 5xi7.pdb, 5xiw.pdb, 5xke.pdb, 5xkf.pdb, 5xkg.pdb, 5xkh.pdb, 5xlt.pdb, 5xlz.pdb, 5xp3.pdb, 5yl2.pdb, 5yl4.pdb, 5ylj.pdb, 5yls.pdb, 5yz3.pdb, 6br1.pdb, 6brf.pdb, 6bry.pdb, 6bs2.pdb, 6d88.pdb, 6eg5.pdb, 6fii.pdb, 6fjf.pdb, 6fjm.pdb, 6fkj.pdb, 6fkl.pdb, 6gj4.pdb, 6i5c.pdb, 6k9v.pdb, 6knz.pdb, 6lsm.pdb, 6lsn.pdb, 6n47.pdb, 6nng.pdb, 6o5m.pdb, 6o5n.pdb, 6pc4.pdb, 6qtn.pdb, 6s9e.pdb, 6y4n.pdb, 6y6d.pdb, 7au5.pdb, 7cda.pdb, 7ce6.pdb, 7ce8.pdb, 7cek.pdb, 7cld.pdb, 7cnm.pdb, 7cnn.pdb, 7cno.pdb, 7cpd.pdb, 7cpq.pdb, 7dad.pdb, 7dae.pdb, 7daf.pdb, 7db9.pdb, 7dba.pdb, 7dbb.pdb, 7dbc.pdb, 7dbd.pdb, 7dp8.pdb, 7e4q.pdb, 7e4r.pdb, 7e4z.pdb, 8a0l.pdb, 5fnv.pdb, 3tig.pdb, 3tii.pdb, 3tin.pdb, 4o4h.pdb, 4o4l.pdb, 4o4i.pdb, 5j2u.pdb, 1w93.pdb, 1w96.pdb, 1ulz.pdb, 1bnc.pdb, 1dv1.pdb, 1dv2.pdb, 2gps.pdb, 2gpw.pdb, 2j9g.pdb, 2v58.pdb, 2v59.pdb, 2v5a.pdb, 2vr1.pdb, 2w6m.pdb, 2w6n.pdb, 2w6o.pdb, 2w6p.pdb, 2w6q.pdb, 2w6z.pdb, 2w70.pdb, 2w71.pdb, 3jzf.pdb, 3jzi.pdb, 3rup.pdb, 3rv3.pdb, 3rv4.pdb, 4hr7.pdb, 1a9x.pdb, 1bxr.pdb, 1c30.pdb, 1c3o.pdb, 1ce8.pdb, 1cs0.pdb, 1jdb.pdb, 1kee.pdb, 1m6v.pdb, 1t36.pdb, 1gso.pdb, 1vkz.pdb, 1eyz.pdb, 1ez1.pdb, 1kj8.pdb, 1kj9.pdb, 1kji.pdb, 1kjj.pdb, 1kjq.pdb, 1b6r.pdb, 1b6s.pdb, 3eth.pdb, 3etj.pdb, 3g8c.pdb, 3g8d.pdb, 6oi9.pdb, 3mjf.pdb, 1auv.pdb, 1aux.pdb, 1pk8.pdb, 1px2.pdb, 1i7l.pdb, 1i7n.pdb, 2p0a.pdb, 1dik.pdb, 1ggo.pdb, 1jde.pdb, 1kbl.pdb, 1kc7.pdb, 2dik.pdb, 2r82.pdb, 1vbg.pdb, 1vbh.pdb, 2x0s.pdb, 1h6z.pdb, 1m0t.pdb, 1m0w.pdb, 2hgs.pdb, 1uc8.pdb, 1uc9.pdb, 3vpd.pdb, 2io7.pdb, 2io8.pdb, 2io9.pdb, 2ioa.pdb, 2iob.pdb, 2r7k.pdb, 2r7l.pdb, 2r7m.pdb, 2r7n.pdb, 2r84.pdb, 2r85.pdb, 2r86.pdb, 2r87.pdb, 2pbz.pdb, 4gaa.pdb, 3hq2.pdb, 2fpq.pdb, 2qn0.pdb, 2w2d.pdb, 2xhl.pdb, 3deb.pdb, 6f47.pdb, 6f4e.pdb, 6g8u.pdb, 6g8v.pdb, 7kz7.pdb, 1yvg.pdb, 1z7h.pdb, 4j7l.pdb, 5giv.pdb, 5e3x.pdb, 2c6f.pdb, 2c6n.pdb, 2ddy.pdb, 2mze.pdb, 2mzh.pdb, 2mzi.pdb, 2xyd.pdb, 3b8z.pdb, 3dwb.pdb, 3edg.pdb, 3edh.pdb, 3edi.pdb, 3hy7.pdb, 3hy9.pdb, 3hyg.pdb, 3ljt.pdb, 3mdj.pdb, 3nxq.pdb, 3qnf.pdb, 3se6.pdb, 4bxk.pdb, 4bzs.pdb, 4ca6.pdb, 4dd8.pdb, 4fyq.pdb, 4fyr.pdb, 4fyt.pdb, 4jbs.pdb, 4p8q.pdb, 4pj6.pdb, 4ufa.pdb, 4ufb.pdb, 4wk7.pdb, 4wke.pdb, 4wki.pdb, 5am9.pdb, 5ama.pdb, 5amc.pdb, 5cu5.pdb, 5j6s.pdb, 5k1v.pdb, 5lhd.pdb, 5mj6.pdb, 5ue2.pdb, 5ue5.pdb, 6bsl.pdb, 6bsm.pdb, 6btn.pdb, 6bto.pdb, 6btp.pdb, 6btq.pdb, 6ea4.pdb, 6en5.pdb, 6en6.pdb, 6f9r.pdb, 6f9v.pdb, 6h5x.pdb, 6q4r.pdb, 6t6r.pdb, 6tt1.pdb, 6tt3.pdb, 6tt4.pdb, 6u7e.pdb, 6u7f.pdb, 6u7g.pdb, 6yjm.pdb, 6zpq.pdb, 6zpt.pdb, 7q24.pdb, 4iuw.pdb, 6ya1.pdb, 6yze.pdb, 3zuk.pdb, 6xly.pdb, 4quo.pdb, 4fke.pdb, 4fkh.pdb, 4fkk.pdb, 4h5h.pdb, 4hol.pdb, 4hom.pdb, 4naq.pdb, 4nz8.pdb, 4ou3.pdb, 5lds.pdb, 5lg6.pdb, 5z65.pdb, 6buy.pdb, 6bv0.pdb, 6bv1.pdb, 6bv2.pdb, 6bv3.pdb, 6bv4.pdb, 4j4m.pdb, 5d7w.pdb, 4r3v.pdb, 3q7j.pdb, 5wvu.pdb, 3hoa.pdb, 7a03.pdb, 1c7k.pdb, 1kuh.pdb, 4hx3.pdb, 1bkc.pdb, 1zxc.pdb, 2a8h.pdb, 2ddf.pdb, 2fv5.pdb, 2fv9.pdb, 3b92.pdb, 3cki.pdb, 3e8r.pdb, 3edz.pdb, 3ewj.pdb, 3g42.pdb, 3kmc.pdb, 3kme.pdb, 3l0t.pdb, 3l0v.pdb, 3le9.pdb, 3lea.pdb, 3lgp.pdb, 3o64.pdb, 2i47.pdb, 2oi0.pdb, 1eub.pdb, 1fls.pdb, 1fm1.pdb, 1xuc.pdb, 1xud.pdb, 1xur.pdb, 1you.pdb, 1ztq.pdb, 2d1n.pdb, 2e2d.pdb, 2ow9.pdb, 2ozr.pdb, 2pjt.pdb, 2yig.pdb, 3elm.pdb, 3i7g.pdb, 3i7i.pdb, 3kec.pdb, 3kej.pdb, 3kek.pdb, 3kry.pdb, 3ljz.pdb, 3o2x.pdb, 3tvc.pdb, 3wv1.pdb, 3wv2.pdb, 3wv3.pdb, 3zxh.pdb, 456c.pdb, 4a7b.pdb, 4fu4.pdb, 4fvl.pdb, 4g0d.pdb, 4jp4.pdb, 4jpa.pdb, 4l19.pdb, 5b5o.pdb, 5b5p.pdb, 5bot.pdb, 5boy.pdb, 5bpa.pdb, 5uwk.pdb, 5uwl.pdb, 5uwm.pdb, 6hv2.pdb, 7ju8.pdb, 830c.pdb, 1cxv.pdb, 1ayk.pdb, 1cge.pdb, 1cgf.pdb, 1cgl.pdb, 1hfc.pdb, 1su3.pdb, 2ayk.pdb, 2j0t.pdb, 2tcl.pdb, 3ayk.pdb, 3shi.pdb, 4ayk.pdb, 966c.pdb, 1fbl.pdb, 1ck7.pdb, 1eak.pdb, 1gxd.pdb, 1qib.pdb, 1gkc.pdb, 1gkd.pdb, 1l6j.pdb, 2ovx.pdb, 2ovz.pdb, 2ow0.pdb, 2ow1.pdb, 2ow2.pdb, 4h1q.pdb, 4h2e.pdb, 4h3x.pdb, 4h82.pdb, 4hma.pdb, 4wzv.pdb, 4xct.pdb, 5cuh.pdb, 5i12.pdb, 6esm.pdb, 1jiz.pdb, 1jk3.pdb, 1os2.pdb, 1os9.pdb, 1rmz.pdb, 1ros.pdb, 1utt.pdb, 1utz.pdb, 1y93.pdb, 1ycm.pdb, 2hu6.pdb, 2k2g.pdb, 2k9c.pdb, 2mlr.pdb, 2mls.pdb, 2n8r.pdb, 2oxu.pdb, 2oxw.pdb, 2oxz.pdb, 2poj.pdb, 2wo8.pdb, 2wo9.pdb, 2woa.pdb, 2z2d.pdb, 3ba0.pdb, 3ehx.pdb, 3ehy.pdb, 3lik.pdb, 3lil.pdb, 3lir.pdb, 3ljg.pdb, 3n2u.pdb, 3n2v.pdb, 3rts.pdb, 3rtt.pdb, 3rtu.pdb, 4guy.pdb, 4ijo.pdb, 5cxa.pdb, 5czm.pdb, 5d2b.pdb, 5d3c.pdb, 5i0l.pdb, 5i2z.pdb, 5i3m.pdb, 5i43.pdb, 5i4o.pdb, 5l79.pdb, 5l7f.pdb, 5lab.pdb, 5n5j.pdb, 5n5k.pdb, 6ekn.pdb, 6ela.pdb, 6enm.pdb, 6eox.pdb, 6rd0.pdb, 6rly.pdb, 1mmp.pdb, 1mmq.pdb, 1mmr.pdb, 1rm8.pdb, 1bqq.pdb, 1buv.pdb, 1hov.pdb, 1a85.pdb, 1a86.pdb, 1bzs.pdb, 1i73.pdb, 1i76.pdb, 1jan.pdb, 1jao.pdb, 1jap.pdb, 1jaq.pdb, 1jh1.pdb, 1jj9.pdb, 1kbc.pdb, 1mmb.pdb, 1mnc.pdb, 1zp5.pdb, 1zs0.pdb, 1zvx.pdb, 2oy2.pdb, 2oy4.pdb, 3dng.pdb, 3dpe.pdb, 3dpf.pdb, 4qkz.pdb, 5h8x.pdb, 1b3d.pdb, 1b8y.pdb, 1biw.pdb, 1bm6.pdb, 1bqo.pdb, 1c3i.pdb, 1c8t.pdb, 1caq.pdb, 1ciz.pdb, 1cqr.pdb, 1d5j.pdb, 1d7x.pdb, 1d8f.pdb, 1d8m.pdb, 1g05.pdb, 1g49.pdb, 1g4k.pdb, 1hfs.pdb, 1hy7.pdb, 1oo9.pdb, 1qia.pdb, 1qic.pdb, 1slm.pdb, 1sln.pdb, 1uea.pdb, 1ums.pdb, 1umt.pdb, 1usn.pdb, 2d1o.pdb, 2jnp.pdb, 2jt5.pdb, 2jt6.pdb, 2srt.pdb, 2usn.pdb, 3ohl.pdb, 3oho.pdb, 3usn.pdb, 4dpe.pdb, 4g9l.pdb, 4ja1.pdb, 6mav.pdb, 6n9d.pdb, 1q3a.pdb, 1hv5.pdb, 2gtq.pdb, 4pu2.pdb, 4pvb.pdb, 4pw4.pdb, 4qhp.pdb, 4qir.pdb, 4qme.pdb, 4qpe.pdb, 5dyf.pdb, 2xdt.pdb, 2dy0.pdb, 1gw6.pdb, 1h19.pdb, 1hs6.pdb, 1sqm.pdb, 2r59.pdb, 2vj8.pdb, 3b7r.pdb, 3b7s.pdb, 3b7t.pdb, 3b7u.pdb, 3cho.pdb, 3chp.pdb, 3chq.pdb, 3chr.pdb, 3chs.pdb, 3fh5.pdb, 3fh7.pdb, 3fh8.pdb, 3fhe.pdb, 3fts.pdb, 3ftu.pdb, 3ftv.pdb, 3ftw.pdb, 3ftx.pdb, 3fty.pdb, 3ftz.pdb, 3fu0.pdb, 3fu3.pdb, 3fu5.pdb, 3fu6.pdb, 3fud.pdb, 3fue.pdb, 3fuf.pdb, 3fuh.pdb, 3fui.pdb, 3fuj.pdb, 3fuk.pdb, 3ful.pdb, 3fum.pdb, 3fun.pdb, 3u9w.pdb, 4dpr.pdb, 4l2l.pdb, 4mkt.pdb, 4ms6.pdb, 4r7l.pdb, 4rsy.pdb, 4rvb.pdb, 5bpp.pdb, 5n3w.pdb, 5ni2.pdb, 5ni4.pdb, 5ni6.pdb, 5nia.pdb, 5nid.pdb, 5nie.pdb, 6o5h.pdb, 7llq.pdb, 1z1w.pdb, 1z5h.pdb, 5aen.pdb, 5fwq.pdb, 6enc.pdb, 6end.pdb, 7auz.pdb, 7av0.pdb, 7av1.pdb, 7av2.pdb, 1j7n.pdb, 1jky.pdb, 1pwp.pdb, 1pwq.pdb, 1pwu.pdb, 1pwv.pdb, 1pww.pdb, 1yqy.pdb, 1zxv.pdb, 4dv8.pdb, 4pkq.pdb, 4pks.pdb, 4pkt.pdb, 4pku.pdb, 4pkv.pdb, 4pkw.pdb, 4wf6.pdb, 4xm6.pdb, 4xm7.pdb, 4xm8.pdb, 5d1s.pdb, 5d1t.pdb, 5d1u.pdb, 1oz9.pdb, 1tvi.pdb, 1xm5.pdb, 1xax.pdb, 3cmn.pdb, 3e11.pdb, 2ejq.pdb, 1bqb.pdb, 1ezm.pdb, 1u4g.pdb, 3dbk.pdb, 4k89.pdb, 6f8b.pdb, 6fzx.pdb, 1esp.pdb, 1npc.pdb, 1fj3.pdb, 1fjo.pdb, 1fjq.pdb, 1fjt.pdb, 1fju.pdb, 1fjv.pdb, 1fjw.pdb, 1gxw.pdb, 1hyt.pdb, 1kei.pdb, 1kjo.pdb, 1kjp.pdb, 1kkk.pdb, 1kl6.pdb, 1kr6.pdb, 1kro.pdb, 1ks7.pdb, 1kto.pdb, 1l3f.pdb, 1lna.pdb, 1lnb.pdb, 1lnc.pdb, 1lnd.pdb, 1lne.pdb, 1lnf.pdb, 1no0.pdb, 1os0.pdb, 1pe5.pdb, 1pe7.pdb, 1pe8.pdb, 1qf0.pdb, 1qf1.pdb, 1qf2.pdb, 1thl.pdb, 1tli.pdb, 1tlp.pdb, 1tlx.pdb, 1tmn.pdb, 1y3g.pdb, 1z9g.pdb, 1zdp.pdb, 2a7g.pdb, 2g4z.pdb, 2tli.pdb, 2tlx.pdb, 2tmn.pdb, 2whz.pdb, 2wi0.pdb, 3dnz.pdb, 3do0.pdb, 3do1.pdb, 3do2.pdb, 3eim.pdb, 3f28.pdb, 3f2p.pdb, 3fb0.pdb, 3fbo.pdb, 3fcq.pdb, 3fgd.pdb, 3flf.pdb, 3for.pdb, 3fv4.pdb, 3fvp.pdb, 3fwd.pdb, 3fxp.pdb, 3fxs.pdb, 3ls7.pdb, 3ms3.pdb, 3msa.pdb, 3msf.pdb, 3msn.pdb, 3n21.pdb, 3nn7.pdb, 3p7p.pdb, 3p7q.pdb, 3p7r.pdb, 3p7s.pdb, 3p7t.pdb, 3p7u.pdb, 3p7v.pdb, 3p7w.pdb, 3qgo.pdb, 3qh1.pdb, 3qh5.pdb, 3ssb.pdb, 3t2h.pdb, 3t2i.pdb, 3t2j.pdb, 3t73.pdb, 3t74.pdb, 3t87.pdb, 3t8c.pdb, 3t8d.pdb, 3t8f.pdb, 3t8g.pdb, 3t8h.pdb, 3tli.pdb, 3tmn.pdb, 3zi6.pdb, 4d91.pdb, 4d9w.pdb, 4h57.pdb, 4mtw.pdb, 4mwp.pdb, 4mxj.pdb, 4mzn.pdb, 4n4e.pdb, 4n5p.pdb, 4n66.pdb, 4oi5.pdb, 4ow3.pdb, 4tli.pdb, 4tln.pdb, 4tmn.pdb, 4tnl.pdb, 5dpe.pdb, 5dpf.pdb, 5fsj.pdb, 5fsp.pdb, 5fss.pdb, 5js3.pdb, 5jss.pdb, 5jt9.pdb, 5jvi.pdb, 5jxn.pdb, 5k7t.pdb, 5l3u.pdb, 5l41.pdb, 5l8p.pdb, 5lif.pdb, 5lvd.pdb, 5lwd.pdb, 5m5f.pdb, 5m69.pdb, 5m9w.pdb, 5ma7.pdb, 5mnr.pdb, 5n2t.pdb, 5n2x.pdb, 5n2z.pdb, 5n31.pdb, 5n34.pdb, 5n3v.pdb, 5n3y.pdb, 5o8n.pdb, 5t9i.pdb, 5t9k.pdb, 5t9q.pdb, 5tac.pdb, 5tad.pdb, 5tae.pdb, 5tai.pdb, 5taj.pdb, 5tak.pdb, 5tli.pdb, 5tln.pdb, 5tmn.pdb, 5un3.pdb, 5uu7.pdb, 5uu8.pdb, 5uu9.pdb, 5uua.pdb, 5uub.pdb, 5uuc.pdb, 5uud.pdb, 5uue.pdb, 6d5n.pdb, 6d5o.pdb, 6d5p.pdb, 6d5q.pdb, 6d5r.pdb, 6d5s.pdb, 6d5t.pdb, 6d5u.pdb, 6fj2.pdb, 6ig7.pdb, 6lzn.pdb, 6lzo.pdb, 6n4w.pdb, 6n4z.pdb, 6qar.pdb, 6qf2.pdb, 6qf3.pdb, 6sel.pdb, 6tli.pdb, 6tmn.pdb, 7akn.pdb, 7tli.pdb, 7tln.pdb, 8tli.pdb, 8tln.pdb, 5dlh.pdb, 5wr2.pdb, 5wr3.pdb, 5wr4.pdb, 5wr5.pdb, 5wr6.pdb, 6fsm.pdb, 6ghx.pdb, 6y4i.pdb, 6yi6.pdb, 6ymr.pdb, 6yms.pdb, 5hqd.pdb, 4b52.pdb, 4ger.pdb, 7ecc.pdb, 3nqy.pdb, 1lml.pdb, 1dmt.pdb, 1r1h.pdb, 1r1i.pdb, 1r1j.pdb, 1y8j.pdb, 2qpj.pdb, 5jmy.pdb, 6gid.pdb, 6suk.pdb, 6svy.pdb, 6thp.pdb, 6xvp.pdb, 2yb9.pdb, 4cth.pdb, 4xbh.pdb, 4zr5.pdb, 5v48.pdb, 7c8d.pdb, 1r42.pdb, 1r4l.pdb, 2ajf.pdb, 3d0g.pdb, 3d0h.pdb, 3d0i.pdb, 3sci.pdb, 3scj.pdb, 3sck.pdb, 3scl.pdb, 6lzg.pdb, 6m0j.pdb, 6vw1.pdb, 7bh9.pdb, 7drv.pdb, 7dx6.pdb, 7dx8.pdb, 7efp.pdb, 7efr.pdb, 7ekc.pdb, 7eke.pdb, 7ekf.pdb, 7ekg.pdb, 7ekh.pdb, 7fdi.pdb, 7l0n.pdb, 7lo4.pdb, 7mjm.pdb, 7nxc.pdb, 7t9k.pdb, 7u0n.pdb, 7xaz.pdb, 7xb1.pdb, 7ya1.pdb, 7fc3.pdb, 1j36.pdb, 1j37.pdb, 1j38.pdb, 1o86.pdb, 1o8a.pdb, 1uze.pdb, 1uzf.pdb, 2oc2.pdb, 2xy9.pdb, 3bkk.pdb, 3bkl.pdb, 3l3n.pdb, 4bzr.pdb, 4ca5.pdb, 6f9t.pdb, 6f9u.pdb, 6h5w.pdb, 6zpu.pdb, 7q28.pdb, 7q29.pdb, 1s4b.pdb, 2o36.pdb, 1i1i.pdb, 1k9x.pdb, 1ka2.pdb, 1ka4.pdb, 6s1y.pdb, 6s1z.pdb, 2x8y.pdb, 2x8z.pdb, 2x90.pdb, 2x91.pdb, 2x92.pdb, 2x93.pdb, 2x94.pdb, 2x95.pdb, 2x96.pdb, 2x97.pdb, 2xhm.pdb, 3zqz.pdb, 4aa1.pdb, 4ca7.pdb, 4ca8.pdb, 5a2r.pdb, 2iul.pdb, 2iux.pdb, 2ydm.pdb, 4c2n.pdb, 4c2o.pdb, 4c2p.pdb, 4c2q.pdb, 4c2r.pdb, 2o3e.pdb, 4fxy.pdb, 7dhx.pdb, 1go7.pdb, 1go8.pdb, 1k7g.pdb, 1k7i.pdb, 1k7q.pdb, 3hb2.pdb, 3hbu.pdb, 3hbv.pdb, 3hda.pdb, 1akl.pdb, 1jiw.pdb, 1kap.pdb, 1g9k.pdb, 1h71.pdb, 1o0q.pdb, 1o0t.pdb, 1om6.pdb, 1om7.pdb, 1om8.pdb, 1omj.pdb, 1af0.pdb, 1sat.pdb, 1smp.pdb, 1srp.pdb, 3u1r.pdb, 6ixx.pdb, 1e1h.pdb, 1xtf.pdb, 1xtg.pdb, 2g7k.pdb, 2g7n.pdb, 2ilp.pdb, 2ima.pdb, 2imb.pdb, 2imc.pdb, 2nyy.pdb, 3bok.pdb, 3bon.pdb, 3boo.pdb, 3bta.pdb, 3ds9.pdb, 3dse.pdb, 3nf3.pdb, 3qw5.pdb, 3qw6.pdb, 3qw7.pdb, 3qw8.pdb, 4ej5.pdb, 4hev.pdb, 4ks6.pdb, 4ktx.pdb, 4kuf.pdb, 5vgv.pdb, 5vgx.pdb, 7kyf.pdb, 1epw.pdb, 1f31.pdb, 1f82.pdb, 1fqh.pdb, 1g9a.pdb, 1g9b.pdb, 1g9c.pdb, 1g9d.pdb, 1i1e.pdb, 1s0b.pdb, 1s0c.pdb, 1s0d.pdb, 1s0e.pdb, 1s0f.pdb, 1s0g.pdb, 2etf.pdb, 2np0.pdb, 1t3a.pdb, 1t3c.pdb, 3d3x.pdb, 1zkw.pdb, 1zkx.pdb, 1zl5.pdb, 1zl6.pdb, 1zn3.pdb, 2a8a.pdb, 2a97.pdb, 2ise.pdb, 2isg.pdb, 2ish.pdb, 3bwi.pdb, 3c88.pdb, 3c89.pdb, 3c8a.pdb, 3c8b.pdb, 3fie.pdb, 3fii.pdb, 4el4.pdb, 4elc.pdb, 6xcb.pdb, 6xcc.pdb, 6xcd.pdb, 6xce.pdb, 6xcf.pdb, 7dvl.pdb, 7ky2.pdb, 3qix.pdb, 3qiy.pdb, 3qiz.pdb, 3qj0.pdb, 1ast.pdb, 1iaa.pdb, 1iab.pdb, 1iac.pdb, 1iad.pdb, 1iae.pdb, 1qji.pdb, 1qjj.pdb, 6saz.pdb, 6ht9.pdb, 1r54.pdb, 1r55.pdb, 1qua.pdb, 1aig.pdb, 2aig.pdb, 3aig.pdb, 4aig.pdb, 1bsw.pdb, 1bud.pdb, 1wni.pdb, 1kuf.pdb, 1kui.pdb, 1kug.pdb, 1kuk.pdb, 1nd1.pdb, 2w12.pdb, 2w13.pdb, 2w14.pdb, 2w15.pdb, 1atl.pdb, 1dth.pdb, 1htd.pdb, 4q1l.pdb, 3gbo.pdb, 6x5x.pdb, 1yp1.pdb, 6wlw.pdb, 6wm2.pdb, 1r5z.pdb, 1v9m.pdb, 3j9t.pdb, 6o7u.pdb, 6m0r.pdb, 6pe4.pdb, 3qsd.pdb, 3s3q.pdb, 3s3r.pdb, 4i07.pdb, 5ogq.pdb, 5ogr.pdb, 6yi7.pdb, 3u8e.pdb, 5a24.pdb, 2o6x.pdb, 4yyq.pdb, 4yyr.pdb, 4yyu.pdb, 4yyv.pdb, 4yyw.pdb, 1dkv.pdb, 2etl.pdb, 2nqd.pdb, 2xu3.pdb, 2xu4.pdb, 2xu5.pdb, 2y5b.pdb, 2yj2.pdb, 2yj8.pdb, 2yj9.pdb, 2yjb.pdb, 2yjc.pdb, 2zfy.pdb, 3a7s.pdb, 3bc3.pdb, 3h89.pdb, 3h8b.pdb, 3h8c.pdb, 3i3t.pdb, 3iv2.pdb, 3mtn.pdb, 3of8.pdb, 3of9.pdb, 3rii.pdb, 3ris.pdb, 3sqa.pdb, 3tb3.pdb, 3von.pdb, 4axl.pdb, 4axm.pdb, 4i6l.pdb, 5cvm.pdb, 5f02.pdb, 5j94.pdb, 5ja7.pdb, 5k16.pdb, 5k1c.pdb, 5l8h.pdb, 5l8w.pdb, 5mae.pdb, 5maj.pdb, 5mqy.pdb, 5ohk.pdb, 5ohp.pdb, 6cpm.pdb, 6crn.pdb, 6ezp.pdb, 6gh9.pdb, 6gha.pdb, 6jlq.pdb, 6ml1.pdb, 6p3q.pdb, 6ql8.pdb, 2wdt.pdb, 2we6.pdb, 5cht.pdb, 5chv.pdb, 4guz.pdb, 4dhi.pdb, 4dhj.pdb, 3d9w.pdb, 6k9n.pdb, 6k9p.pdb, 6kbe.pdb, 2b1m.pdb, 6mis.pdb, 6okj.pdb, 6u7d.pdb, 6y6l.pdb, 6ycb.pdb, 6ycc.pdb, 6ycd.pdb, 6yce.pdb, 6ycf.pdb, 6ycg.pdb, 3phz.pdb, 5mua.pdb, 2bsz.pdb, 6zq3.pdb, 3f75.pdb, 4i6n.pdb, 4hwy.pdb, 7e3f.pdb, 5fpw.pdb, 3hhi.pdb, 3mor.pdb, 3qj3.pdb, 3qt4.pdb, 1ito.pdb, 1qdq.pdb, 1sp4.pdb, 1csb.pdb, 1gmy.pdb, 1huc.pdb, 2pbh.pdb, 3pbh.pdb, 1cpj.pdb, 1cte.pdb, 1mir.pdb, 1the.pdb, 1atk.pdb, 1au0.pdb, 1au2.pdb, 1au3.pdb, 1au4.pdb, 1ayu.pdb, 1ayv.pdb, 1ayw.pdb, 1bgo.pdb, 1by8.pdb, 1mem.pdb, 1nl6.pdb, 1nlj.pdb, 1q6k.pdb, 1snk.pdb, 1tu6.pdb, 1u9v.pdb, 1u9w.pdb, 1u9x.pdb, 1vsn.pdb, 1yk7.pdb, 1yk8.pdb, 1yt7.pdb, 2ato.pdb, 2aux.pdb, 2auz.pdb, 2bdl.pdb, 2fdz.pdb, 2ftd.pdb, 2r6n.pdb, 3c9e.pdb, 3kw9.pdb, 3kwb.pdb, 3kwz.pdb, 3kx1.pdb, 3o0u.pdb, 3o1g.pdb, 3ovz.pdb, 4dmx.pdb, 4dmy.pdb, 4n79.pdb, 4n8w.pdb, 4x6h.pdb, 4x6i.pdb, 4x6j.pdb, 4yv8.pdb, 4yva.pdb, 5jh3.pdb, 5tdi.pdb, 5tun.pdb, 6ash.pdb, 6hgy.pdb, 6pxf.pdb, 6qbs.pdb, 6qlx.pdb, 7pck.pdb, 5t6u.pdb, 6bki.pdb, 2f7d.pdb, 1cjl.pdb, 1cs8.pdb, 1icf.pdb, 1mhw.pdb, 1glo.pdb, 1ms6.pdb, 1npz.pdb, 1nqc.pdb, 2f1g.pdb, 2fye.pdb, 2g6d.pdb, 2g7y.pdb, 2h7j.pdb, 2hh5.pdb, 2hhn.pdb, 2hxz.pdb, 2op3.pdb, 2r9m.pdb, 2r9n.pdb, 2r9o.pdb, 3iej.pdb, 3kwn.pdb, 3mpe.pdb, 3mpf.pdb, 3n3g.pdb, 3n4c.pdb, 3ovx.pdb, 5qbw.pdb, 5qbz.pdb, 5qcd.pdb, 5qcf.pdb, 5qci.pdb, 1fh0.pdb, 3h6s.pdb, 3kqf.pdb, 1deu.pdb, 1ef7.pdb, 1aec.pdb, 2act.pdb, 1a6r.pdb, 1gcb.pdb, 2dzy.pdb, 2dzz.pdb, 2e00.pdb, 2e01.pdb, 2e02.pdb, 2e03.pdb, 3gcb.pdb, 1cb5.pdb, 2cb5.pdb, 1meg.pdb, 1pci.pdb, 1ppo.pdb, 1k3b.pdb, 1jqp.pdb, 1m6d.pdb, 1nb3.pdb, 1nb5.pdb, 8pch.pdb, 1yal.pdb, 1aim.pdb, 1ewl.pdb, 1ewm.pdb, 1ewo.pdb, 1ewp.pdb, 1f29.pdb, 1f2a.pdb, 1f2b.pdb, 1f2c.pdb, 1me3.pdb, 1me4.pdb, 1u9q.pdb, 2aim.pdb, 2efm.pdb, 2oz2.pdb, 3hd3.pdb, 3i06.pdb, 3iut.pdb, 3kku.pdb, 3lxs.pdb, 4klb.pdb, 4pi3.pdb, 4qh6.pdb, 4w5b.pdb, 4xui.pdb, 6n3s.pdb, 6o2x.pdb, 6ux6.pdb, 7juj.pdb, 1iwd.pdb, 1o0e.pdb, 1yvb.pdb, 2ghu.pdb, 2oul.pdb, 3bpf.pdb, 3pnr.pdb, 1gec.pdb, 1xkg.pdb, 2as8.pdb, 3f5v.pdb, 1bp4.pdb, 1bqi.pdb, 1cvz.pdb, 1khp.pdb, 1khq.pdb, 1pad.pdb, 1pe6.pdb, 1pip.pdb, 1pop.pdb, 1ppd.pdb, 1ppn.pdb, 1ppp.pdb, 1stf.pdb, 2cio.pdb, 2pad.pdb, 3e1z.pdb, 3ima.pdb, 3lfy.pdb, 4pad.pdb, 5pad.pdb, 6h8t.pdb, 6pad.pdb, 9pap.pdb, 1cqd.pdb, 1pxv.pdb, 1x9y.pdb, 1y4h.pdb, 1cv8.pdb, 1dki.pdb, 1pvj.pdb, 1s4v.pdb, 3p5u.pdb, 3p5v.pdb, 3p5w.pdb, 3p5x.pdb, 3ioq.pdb, 2dc6.pdb, 2dc7.pdb, 2dc8.pdb, 2dc9.pdb, 2dca.pdb, 2dcb.pdb, 2dcc.pdb, 2dcd.pdb, 2psc.pdb, 4yys.pdb, 2c0y.pdb, 2fq9.pdb, 2fra.pdb, 2frq.pdb, 2ft2.pdb, 2fud.pdb, 3pdf.pdb, 6jd0.pdb, 6jd8.pdb, 3bpm.pdb, 3bwk.pdb, 4bpv.pdb, 4bqv.pdb, 4bs5.pdb, 4bs6.pdb, 4bsq.pdb, 4mzo.pdb, 4mzs.pdb, 2pns.pdb, 2pre.pdb, 3bcn.pdb, 2p7u.pdb, 2p86.pdb, 6ex8.pdb, 6exo.pdb, 6exq.pdb, 6p4e.pdb, 1ukf.pdb, 1tff.pdb, 4fjv.pdb, 5qio.pdb, 5qip.pdb, 5qiq.pdb, 5qir.pdb, 5qis.pdb, 5qit.pdb, 5qiu.pdb, 5qiv.pdb, 5qiw.pdb, 5qix.pdb, 5qiy.pdb, 5qiz.pdb, 1y08.pdb, 2au1.pdb, 2im9.pdb, 2btw.pdb, 2bu3.pdb, 2evr.pdb, 2fg0.pdb, 2ebf.pdb, 2ebh.pdb, 2ec5.pdb, 2iho.pdb, 3ef2.pdb, 2hly.pdb, 1qmy.pdb, 1qol.pdb, 2jqf.pdb, 2j7q.pdb, 2if6.pdb, 2p82.pdb, 2d1i.pdb, 2z0d.pdb, 2cy7.pdb, 2z0e.pdb, 2zzp.pdb, 4p16.pdb, 4pt5.pdb, 4rna.pdb, 5v69.pdb, 5v6a.pdb, 5w8t.pdb, 5w8u.pdb, 4rez.pdb, 4rf0.pdb, 4rf1.pdb, 2fe8.pdb, 6w9c.pdb, 4r3d.pdb, 4wur.pdb, 5tl6.pdb, 5y3e.pdb, 5y3q.pdb, 3e9s.pdb, 4m0w.pdb, 4mm3.pdb, 5tl7.pdb, 4ovz.pdb, 4ow0.pdb, 6wrh.pdb, 6wzu.pdb, 6xa9.pdb, 6xaa.pdb, 6xg3.pdb, 6yva.pdb, 7cjd.pdb, 7cmd.pdb, 7d47.pdb, 7d6h.pdb, 7d7k.pdb, 7d7l.pdb, 7e35.pdb, 7jir.pdb, 7jit.pdb, 7jiv.pdb, 7jiw.pdb, 7jn2.pdb, 7jrn.pdb, 7koj.pdb, 7kok.pdb, 7kol.pdb, 7krx.pdb, 7lbr.pdb, 7lbs.pdb, 7llf.pdb, 7llz.pdb, 7los.pdb, 7m1y.pdb, 7ofs.pdb, 7oft.pdb, 7qcg.pdb, 7rbs.pdb, 7uv5.pdb, 2p0r.pdb, 1ziv.pdb, 1kfu.pdb, 1kfx.pdb, 1df0.pdb, 1mdw.pdb, 1u5i.pdb, 1kxr.pdb, 1qxp.pdb, 1tl9.pdb, 1tlo.pdb, 2g8e.pdb, 2g8j.pdb, 2nqg.pdb, 2nqi.pdb, 2r9c.pdb, 2r9f.pdb, 1x3w.pdb, 2f4m.pdb, 1bl2.pdb, 1evu.pdb, 1ex0.pdb, 1f13.pdb, 1fie.pdb, 1ggt.pdb, 1ggu.pdb, 1ggy.pdb, 1qrk.pdb, 1l9m.pdb, 1l9n.pdb, 1nud.pdb, 1nuf.pdb, 1nug.pdb, 1rle.pdb, 1rll.pdb, 1sgx.pdb, 1vjj.pdb, 1kv3.pdb, 2q3z.pdb, 3ly6.pdb, 4pyg.pdb, 6a8p.pdb, 1god.pdb, 1x3z.pdb, 4kty.pdb, 2f4o.pdb, 1gx3.pdb, 1w5r.pdb, 1w6f.pdb, 1w4t.pdb, 1e2t.pdb, 4nv7.pdb, 4nv8.pdb, 2vfb.pdb, 2vfc.pdb, 4b55.pdb, 4c5p.pdb, 3ltw.pdb, 4bgf.pdb, 2len.pdb, 4dm9.pdb, 4jkj.pdb, 1uch.pdb, 1xd3.pdb, 6isu.pdb, 1cmx.pdb, 3ifw.pdb, 3irt.pdb, 3kvf.pdb, 3kw5.pdb, 1avp.pdb, 1nln.pdb, 4ekf.pdb, 2bzp.pdb, 2ckg.pdb, 2ckh.pdb, 2g4d.pdb, 2iy0.pdb, 2iy1.pdb, 2iyc.pdb, 6nnq.pdb, 1tgz.pdb, 1th0.pdb, 2io0.pdb, 2io1.pdb, 2io2.pdb, 2io3.pdb, 5aek.pdb, 1xt9.pdb, 2bkq.pdb, 1euv.pdb, 2hkp.pdb, 2hl8.pdb, 2hl9.pdb, 2bkr.pdb, 2xph.pdb, 2xre.pdb, 3zo5.pdb, 1iu4.pdb, 2ayn.pdb, 2ayo.pdb, 6iik.pdb, 6iil.pdb, 6iim.pdb, 6iin.pdb, 6lvs.pdb, 2hd5.pdb, 2ibi.pdb, 5xu8.pdb, 5xve.pdb, 6dgf.pdb, 1vjv.pdb, 1nb8.pdb, 1nbf.pdb, 4m5w.pdb, 4m5x.pdb, 5kyb.pdb, 5kyc.pdb, 5kyd.pdb, 5kye.pdb, 5kyf.pdb, 5n9r.pdb, 5n9t.pdb, 5nge.pdb, 5ngf.pdb, 5uqv.pdb, 5uqx.pdb, 5vs6.pdb, 5vsb.pdb, 5whc.pdb, 6f5h.pdb, 6m1k.pdb, 6vn2.pdb, 6vn3.pdb, 6vn4.pdb, 6vn5.pdb, 7cm2.pdb, 2gfo.pdb, 3n3k.pdb, 3nhe.pdb, 3v6c.pdb, 3v6e.pdb, 1aco.pdb, 1ami.pdb, 1amj.pdb, 1c96.pdb, 1c97.pdb, 1fgh.pdb, 1nis.pdb, 1nit.pdb, 8acn.pdb, 1b0j.pdb, 1b0k.pdb, 1b0m.pdb, 5acn.pdb, 6acn.pdb, 7acn.pdb, 1l5j.pdb, 2b3x.pdb, 2b3y.pdb, 2ipy.pdb, 3sn2.pdb, 3snp.pdb, 6sdk.pdb, 7bm8.pdb, 7ng0.pdb, 1vz0.pdb, 1vk1.pdb, 5x0b.pdb, 5x0e.pdb, 5x0f.pdb, 5x0g.pdb, 5x0j.pdb, 5x0k.pdb, 2hwj.pdb, 1xw3.pdb, 1xw4.pdb, 1yzs.pdb, 2b6f.pdb, 3cyi.pdb, 1q16.pdb, 1siw.pdb, 1y4z.pdb, 1y5i.pdb, 1y5l.pdb, 1y5n.pdb, 3egw.pdb, 3ir5.pdb, 3ir6.pdb, 3ir7.pdb, 1aij.pdb, 1ds8.pdb, 1dv3.pdb, 1dv6.pdb, 1e14.pdb, 1e6d.pdb, 1f6n.pdb, 1fnp.pdb, 1fnq.pdb, 1jgw.pdb, 1jgx.pdb, 1jgy.pdb, 1jgz.pdb, 1jh0.pdb, 1k6l.pdb, 1k6n.pdb, 1kby.pdb, 1l9b.pdb, 1l9j.pdb, 1m3x.pdb, 1mps.pdb, 1ogv.pdb, 1pcr.pdb, 1pss.pdb, 1pst.pdb, 1qov.pdb, 1rg5.pdb, 1rgn.pdb, 1rqk.pdb, 1rvj.pdb, 1ry5.pdb, 1rzh.pdb, 1rzz.pdb, 1s00.pdb, 1umx.pdb, 1yf6.pdb, 1yst.pdb, 1z9k.pdb, 2gmr.pdb, 2rcr.pdb, 3dsy.pdb, 3dtr.pdb, 3dts.pdb, 3du2.pdb, 3du3.pdb, 3duq.pdb, 3v3y.pdb, 3v3z.pdb, 4h99.pdb, 4h9l.pdb, 4hbh.pdb, 4hbj.pdb, 4in5.pdb, 4in6.pdb, 4in7.pdb, 4lwy.pdb, 4n7k.pdb, 4n7l.pdb, 4rcr.pdb, 4tqq.pdb, 5lse.pdb, 6z02.pdb, 6z1j.pdb, 6z27.pdb, 7f0l.pdb, 7mh5.pdb, 7p17.pdb, 1dxr.pdb, 1prc.pdb, 1r2c.pdb, 1txw.pdb, 2jbl.pdb, 2prc.pdb, 3d38.pdb, 3prc.pdb, 4prc.pdb, 5nj4.pdb, 5o4c.pdb, 5prc.pdb, 6prc.pdb, 6zhw.pdb, 6zi4.pdb, 6zi5.pdb, 6zi6.pdb, 6zi9.pdb, 6zia.pdb, 6zid.pdb, 7prc.pdb, 1eys.pdb, 7vnm.pdb, 2boz.pdb, 5lri.pdb, 2axt.pdb, 4pj0.pdb, 7nho.pdb, 7nhp.pdb, 7nhq.pdb, 3a0h.pdb, 5h2f.pdb, 5zzn.pdb, 4il6.pdb, 7d1t.pdb, 7d1u.pdb, 7eda.pdb, 1vrn.pdb, 2i5n.pdb, 2x5u.pdb, 2x5v.pdb, 3g7f.pdb, 3t6d.pdb, 3t6e.pdb, 6et5.pdb, 6kac.pdb, 5xnl.pdb, 2bnp.pdb, 2bns.pdb, 2gnu.pdb, 2hg3.pdb, 2hg9.pdb, 2hh1.pdb, 2hhk.pdb, 2hit.pdb, 2hj6.pdb, 2j8c.pdb, 2j8d.pdb, 2jiy.pdb, 2jj0.pdb, 2uws.pdb, 2uwt.pdb, 2uwu.pdb, 2uwv.pdb, 2uww.pdb, 2ux3.pdb, 2ux4.pdb, 2ux5.pdb, 2uxj.pdb, 2uxk.pdb, 2uxl.pdb, 2uxm.pdb, 2wx5.pdb, 3i4d.pdb, 3zum.pdb, 3zuw.pdb, 7ddq.pdb, 6z5r.pdb, 6z5s.pdb, 2wjm.pdb, 2wjn.pdb, 6wj6.pdb, 3wmm.pdb, 3wmn.pdb, 5y5s.pdb, 7c52.pdb, 5kaf.pdb, 5kai.pdb, 5mx2.pdb, 5tis.pdb, 3bz1.pdb, 3bz2.pdb, 3arc.pdb, 3wu2.pdb, 4ub6.pdb, 4ub8.pdb, 5b5e.pdb, 5b66.pdb, 5gth.pdb, 5gti.pdb, 5v2c.pdb, 5ws5.pdb, 5ws6.pdb, 6jlj.pdb, 6jlk.pdb, 6jll.pdb, 6jlm.pdb, 6jln.pdb, 6jlo.pdb, 6jlp.pdb, 7cji.pdb, 7cjj.pdb, 7cou.pdb, 7dxa.pdb, 7dxh.pdb, 7c9r.pdb, 7eqd.pdb, 1v5f.pdb, 1v5g.pdb, 1ypd.pdb, 2vk1.pdb, 2vk8.pdb, 2w93.pdb, 7l9r.pdb, 2a1t.pdb, 2a1u.pdb, 3gls.pdb, 4bn4.pdb, 4bv3.pdb, 4bvb.pdb, 4bvh.pdb, 4c7b.pdb, 4i5i.pdb, 4if6.pdb, 4ig9.pdb, 4jsr.pdb, 4jt8.pdb, 4jt9.pdb, 4kxq.pdb, 4o8z.pdb, 5d7n.pdb, 5d6r.pdb, 5dx6.pdb, 5wdg.pdb, 6efg.pdb, 6efh.pdb, 6haf.pdb, 4fee.pdb, 4feg.pdb, 4kgd.pdb, 2g1i.pdb, 2vjy.pdb, 2vk4.pdb, 2v3w.pdb, 6a50.pdb, 3e9y.pdb, 3ea4.pdb, 2h2d.pdb, 2h2f.pdb, 2h2g.pdb, 2h2h.pdb, 2h2i.pdb, 2h4f.pdb, 2h4j.pdb, 5oj7.pdb, 5ojn.pdb, 2wva.pdb, 2wvg.pdb, 2wvh.pdb, 3oe1.pdb, 4zp1.pdb, 5tma.pdb, 1dhs.pdb, 1rlz.pdb, 1roz.pdb, 1rqd.pdb, 6p4v.pdb, 6pgr.pdb, 6wkz.pdb, 6wl6.pdb, 6xxh.pdb, 6xxi.pdb, 6xxj.pdb, 6xxk.pdb, 6xxl.pdb, 6xxm.pdb, 6w3z.pdb, 1efv.pdb, 1o96.pdb, 1o97.pdb, 3clr.pdb, 3cls.pdb, 3clt.pdb, 3clu.pdb, 1efp.pdb, 1jsc.pdb, 1n0h.pdb, 1t9a.pdb, 1t9b.pdb, 1t9c.pdb, 1t9d.pdb, 6bd3.pdb, 6bd9.pdb, 5fem.pdb, 5ims.pdb, 5wkc.pdb, 1ybh.pdb, 1yhy.pdb, 1yhz.pdb, 1yi0.pdb, 1yi1.pdb, 1z8n.pdb, 5k2o.pdb, 5k3s.pdb, 5k6q.pdb, 5k6r.pdb, 5k6t.pdb, 5wj1.pdb, 7u1d.pdb, 1bfd.pdb, 1mcz.pdb, 1pi3.pdb, 1po7.pdb, 1q6z.pdb, 1yno.pdb, 2fn3.pdb, 2fwn.pdb, 3f6b.pdb, 3f6e.pdb, 3fsj.pdb, 3fzn.pdb, 4gg1.pdb, 4gm0.pdb, 4gm1.pdb, 4gm4.pdb, 4gp9.pdb, 4gpe.pdb, 4jd5.pdb, 4ju8.pdb, 4ju9.pdb, 4jua.pdb, 4jub.pdb, 4juc.pdb, 4jud.pdb, 4juf.pdb, 4k9k.pdb, 4k9l.pdb, 4k9m.pdb, 4k9n.pdb, 4k9o.pdb, 4k9p.pdb, 4mpj.pdb, 4mpp.pdb, 4mpr.pdb, 4mq5.pdb, 4mzx.pdb, 4qel.pdb, 5dei.pdb, 5dgd.pdb, 5dgt.pdb, 6m2y.pdb, 6m2z.pdb, 1upa.pdb, 1upb.pdb, 1upc.pdb, 2iht.pdb, 2ihu.pdb, 2ihv.pdb, 1ozf.pdb, 1ozg.pdb, 1ozh.pdb, 1ovm.pdb, 2c31.pdb, 2ji6.pdb, 2ji7.pdb, 2ji8.pdb, 2ji9.pdb, 2jib.pdb, 1pvd.pdb, 1qpb.pdb, 1pyb.pdb, 1zpd.pdb, 1v5e.pdb, 2dji.pdb, 1pow.pdb, 1pox.pdb, 1y9d.pdb, 2ez4.pdb, 2ez8.pdb, 2ez9.pdb, 2ezt.pdb, 2ezu.pdb, 1d4o.pdb, 1djl.pdb, 1pt9.pdb, 1u31.pdb, 1e3t.pdb, 1hzz.pdb, 1nm5.pdb, 1pno.pdb, 1pnq.pdb, 1ptj.pdb, 1u28.pdb, 1u2d.pdb, 1u2g.pdb, 1xlt.pdb, 2fr8.pdb, 2frd.pdb, 2fsv.pdb, 2oo5.pdb, 2oor.pdb, 2bru.pdb, 4j16.pdb, 4j1t.pdb, 1ma3.pdb, 1s7g.pdb, 1yc2.pdb, 1ici.pdb, 1m2g.pdb, 1m2h.pdb, 1m2j.pdb, 1m2k.pdb, 1m2n.pdb, 1q14.pdb, 1q17.pdb, 1q1a.pdb, 1szc.pdb, 1szd.pdb, 1s5p.pdb, 1yc5.pdb, 2h4h.pdb, 2h59.pdb, 3d4b.pdb, 3d81.pdb, 3jr3.pdb, 3pdh.pdb, 2b4y.pdb, 2fzq.pdb, 2nyr.pdb, 5xhs.pdb, 6eqs.pdb, 1j8f.pdb, 4rmg.pdb, 4rmh.pdb, 4rmi.pdb, 4rmj.pdb, 4y6q.pdb, 5d7o.pdb, 5d7p.pdb, 5d7q.pdb, 5dy4.pdb, 5dy5.pdb, 5g4c.pdb, 5mar.pdb, 5mat.pdb, 5y0z.pdb, 5y5n.pdb, 5yql.pdb, 5yqm.pdb, 5yqn.pdb, 5yqo.pdb, 6l72.pdb, 6nr0.pdb, 6qcn.pdb, 2od7.pdb, 2od9.pdb, 3rig.pdb, 3riy.pdb, 3zgo.pdb, 3zgv.pdb, 6enx.pdb, 6eo0.pdb, 1ytl.pdb, 6a0a.pdb, 6a09.pdb, 6a0b.pdb, 5oyh.pdb, 6sir.pdb, 3et6.pdb, 2wz1.pdb, 3uvj.pdb, 4ni2.pdb, 5d0e.pdb, 5d0g.pdb, 5d0h.pdb, 5d15.pdb, 4wp8.pdb, 4wp9.pdb, 4wpa.pdb, 1yk9.pdb, 4p2f.pdb, 4p2m.pdb, 4p2x.pdb, 1ybt.pdb, 1ybu.pdb, 3r5g.pdb, 3mr7.pdb, 2w01.pdb, 1e7p.pdb, 1qla.pdb, 1qlb.pdb, 2bs3.pdb, 2bs4.pdb, 2bs2.pdb, 3p4p.pdb, 4kx6.pdb, 1kf6.pdb, 1kfy.pdb, 1l0v.pdb, 2b76.pdb, 3cir.pdb, 1zoy.pdb, 3ae4.pdb, 3aef.pdb, 3sfd.pdb, 3sfe.pdb, 1nek.pdb, 1nen.pdb, 2acz.pdb, 2wdq.pdb, 2wp9.pdb, 2wu2.pdb, 2wu5.pdb, 7jz2.pdb, 1yq3.pdb, 1yq4.pdb, 2fbw.pdb, 2h88.pdb, 2h89.pdb, 2wqy.pdb, 6myo.pdb, 6myp.pdb, 6myq.pdb, 6myr.pdb, 6mys.pdb, 6myt.pdb, 6myu.pdb, 3p4r.pdb, 3p4s.pdb, 3ae1.pdb, 3ae2.pdb, 3aeb.pdb, 4ytp.pdb, 4yxd.pdb, 4qnz.pdb, 4qo0.pdb, 4qo2.pdb, 2ic8.pdb, 2irv.pdb, 2nrf.pdb, 2o7l.pdb, 2xov.pdb, 2xow.pdb, 2xtu.pdb, 3b44.pdb, 3b45.pdb, 3txt.pdb, 3ubb.pdb, 3zeb.pdb, 3zmh.pdb, 3zmi.pdb, 3zmj.pdb, 3zot.pdb, 4h1d.pdb, 4njn.pdb, 4njp.pdb, 2nr9.pdb, 2xtv.pdb, 3odj.pdb, 2vzo.pdb, 2vzt.pdb, 2vzu.pdb, 2vzv.pdb, 5sv5.pdb, 5ifp.pdb, 5ift.pdb, 5ihr.pdb, 5juv.pdb, 5mgc.pdb, 5mgd.pdb, 5fax.pdb, 5fbz.pdb, 2qkg.pdb, 3eb7.pdb, 6dj4.pdb, 6ovb.pdb, 6owk.pdb, 6wpc.pdb, 7ear.pdb, 4qx0.pdb, 4qx1.pdb, 4qx2.pdb, 4qx3.pdb, 4arx.pdb, 4ary.pdb, 6d8k.pdb, 2vjx.pdb, 2vl4.pdb, 2vmf.pdb, 2vo5.pdb, 2vot.pdb, 2vr4.pdb, 2wbk.pdb, 2vqt.pdb, 2v4v.pdb, 3jqw.pdb, 3jqx.pdb, 3acf.pdb, 3acg.pdb, 3ach.pdb, 3aci.pdb, 2xh6.pdb, 4jkm.pdb, 6cxs.pdb, 3bn6.pdb, 2pqs.pdb, 6xxw.pdb, 6ygq.pdb, 3czj.pdb, 3dym.pdb, 3dyo.pdb, 3dyp.pdb, 3e1f.pdb, 3muy.pdb, 3sep.pdb, 3t08.pdb, 3t09.pdb, 3t0a.pdb, 3t0b.pdb, 3t0d.pdb, 3t2o.pdb, 3t2p.pdb, 3t2q.pdb, 4duv.pdb, 4duw.pdb, 4dux.pdb, 6leg.pdb, 3k46.pdb, 3k4a.pdb, 3k4d.pdb, 3lpf.pdb, 3lpg.pdb, 4jhz.pdb, 5a1a.pdb, 5czk.pdb, 6cvm.pdb, 6drv.pdb, 6kuz.pdb, 6lej.pdb, 6lel.pdb, 6lem.pdb, 6x1q.pdb, 7brs.pdb, 6bjq.pdb, 6bjw.pdb, 6bo6.pdb, 6ed2.pdb, 6u7i.pdb, 2xhn.pdb, 4iug.pdb, 5c70.pdb, 5c71.pdb, 2eid.pdb, 2wq8.pdb, 6xlr.pdb, 6xls.pdb, 6xlt.pdb, 2yc2.pdb, 2yc4.pdb, 2qqj.pdb, 2qqo.pdb, 2wuh.pdb, 2z4f.pdb, 3c8x.pdb, 3czu.pdb, 3hei.pdb, 3hpn.pdb, 3skj.pdb, 4omc.pdb, 4omd.pdb, 4qdq.pdb, 4qdr.pdb, 4ryd.pdb, 4z2a.pdb, 5dn2.pdb, 5dq0.pdb, 5jxg.pdb, 5jxi.pdb, 5mim.pdb, 6fmc.pdb, 6fmf.pdb, 6yd2.pdb, 6yd3.pdb, 6yd4.pdb, 6yd7.pdb, 7azb.pdb, 7b7n.pdb, 7cze.pdb, 6eca.pdb, 1wcq.pdb, 2ber.pdb, 2bq9.pdb, 2bzd.pdb, 3cqo.pdb, 2l9l.pdb, 4xuo.pdb, 4xup.pdb, 5ofx.pdb, 5ofz.pdb, 5odu.pdb, 5ofi.pdb, 1wcu.pdb, 3i2f.pdb, 3i2g.pdb, 3i2h.pdb, 3i2i.pdb, 3i2j.pdb, 3i2k.pdb, 3puh.pdb, 3pui.pdb, 4p08.pdb, 7f65.pdb, 3ida.pdb, 5z18.pdb, 6ec6.pdb, 6jz2.pdb, 6jz3.pdb, 6jz4.pdb, 6jz5.pdb, 6jz6.pdb, 6jz7.pdb, 2cdp.pdb, 2cdo.pdb, 4jkk.pdb, 4jkl.pdb, 3le0.pdb, 3leg.pdb, 3lei.pdb, 3lek.pdb, 4gwi.pdb, 4gwj.pdb, 2j1r.pdb, 2j1s.pdb, 2j1t.pdb, 2j1u.pdb, 2j1v.pdb, 2j22.pdb, 4cuc.pdb, 3og2.pdb, 3ogr.pdb, 3ogs.pdb, 3ogv.pdb, 7ay3.pdb, 6u7j.pdb, 5fui.pdb, 6d4o.pdb, 5z19.pdb, 1gof.pdb, 1gog.pdb, 1goh.pdb, 1k3i.pdb, 1t2x.pdb, 2eib.pdb, 2eic.pdb, 2eie.pdb, 2jkx.pdb, 2vz1.pdb, 2vz3.pdb, 1eut.pdb, 1euu.pdb, 1w8n.pdb, 1w8o.pdb, 1gny.pdb, 1us2.pdb, 1us3.pdb, 1j83.pdb, 1j84.pdb, 1nx9.pdb, 1ryy.pdb, 2b4k.pdb, 2b9v.pdb, 1mpx.pdb, 1ju3.pdb, 1ju4.pdb, 1l7q.pdb, 1l7r.pdb, 1lns.pdb, 1k42.pdb, 1k45.pdb, 1gui.pdb, 1cx1.pdb, 1gu3.pdb, 1ulo.pdb, 1ulp.pdb, 2y64.pdb, 2y6g.pdb, 2y6h.pdb, 2y6j.pdb, 2y6k.pdb, 2y6l.pdb, 3jxs.pdb, 4bj0.pdb, 5dpn.pdb, 1k12.pdb, 1l7l.pdb, 1oko.pdb, 1uoj.pdb, 2vxj.pdb, 2wyf.pdb, 3zyf.pdb, 3zyh.pdb, 4a6s.pdb, 4al9.pdb, 4cp9.pdb, 4cpb.pdb, 4ljh.pdb, 4lk6.pdb, 4lk7.pdb, 4yw6.pdb, 4yw7.pdb, 4ywa.pdb, 5d21.pdb, 5mih.pdb, 6yo3.pdb, 6yoh.pdb, 1hno.pdb, 1pmh.pdb, 1pmj.pdb, 1of3.pdb, 1of4.pdb, 1oh4.pdb, 1gwk.pdb, 1gwl.pdb, 1gwm.pdb, 1oh3.pdb, 1w8t.pdb, 1w8u.pdb, 1w8w.pdb, 1w8z.pdb, 1w90.pdb, 1w9f.pdb, 1kex.pdb, 4rn5.pdb, 5c7g.pdb, 5ijr.pdb, 5iyy.pdb, 5j1x.pdb, 5jgi.pdb, 5jgq.pdb, 5jhk.pdb, 2orx.pdb, 1sdd.pdb, 1czs.pdb, 1czt.pdb, 1czv.pdb, 1d7p.pdb, 1iqd.pdb, 2qqi.pdb, 2qqm.pdb, 2qqn.pdb, 3hnb.pdb, 3hny.pdb, 3hob.pdb, 3i97.pdb, 4ki5.pdb, 4pt6.pdb, 4xzu.pdb, 4mo3.pdb, 1wmd.pdb, 1wme.pdb, 1wmf.pdb, 1p8j.pdb, 1ot5.pdb, 1r64.pdb, 2id4.pdb, 1umh.pdb, 1umi.pdb, 2e33.pdb, 2rj2.pdb, 5b4n.pdb, 1o59.pdb, 1sg3.pdb, 1uww.pdb, 1wmx.pdb, 1wzx.pdb, 2c24.pdb, 1nkg.pdb, 3njv.pdb, 3njx.pdb, 1xoy.pdb, 1tg7.pdb, 1xc6.pdb, 1ux7.pdb, 1w0n.pdb, 2g9f.pdb, 2g9g.pdb, 2i74.pdb, 1i5p.pdb, 1dlc.pdb, 1ji6.pdb, 1w99.pdb, 1ciy.pdb, 1v0a.pdb, 4v0f.pdb, 2lro.pdb, 2lrp.pdb, 1wky.pdb, 2vng.pdb, 2vmg.pdb, 2vmh.pdb, 2vmi.pdb, 2vno.pdb, 2vnr.pdb, 1nqd.pdb, 1nqj.pdb, 2o8o.pdb, 4hpk.pdb, 5iku.pdb, 2quo.pdb, 3am2.pdb, 3x29.pdb, 3ziw.pdb, 3zix.pdb, 2bba.pdb, 2h1e.pdb, 1kgy.pdb, 1nuk.pdb, 1shw.pdb, 3etp.pdb, 2lw8.pdb, 2wo1.pdb, 2wo2.pdb, 2wo3.pdb, 3ckh.pdb, 3gxu.pdb, 3nru.pdb, 3p1i.pdb, 4et7.pdb, 4l0p.pdb, 1yq2.pdb, 1bgl.pdb, 1bgm.pdb, 1dp0.pdb, 1f49.pdb, 1f4a.pdb, 1f4h.pdb, 1gho.pdb, 1hn1.pdb, 1jyn.pdb, 1jyv.pdb, 1jyw.pdb, 1jyx.pdb, 1jyy.pdb, 1jyz.pdb, 1jz0.pdb, 1jz1.pdb, 1jz2.pdb, 1jz3.pdb, 1jz4.pdb, 1jz5.pdb, 1jz6.pdb, 1jz7.pdb, 1jz8.pdb, 1px3.pdb, 1px4.pdb, 3i3b.pdb, 3i3d.pdb, 3i3e.pdb, 3iap.pdb, 3iaq.pdb, 3muz.pdb, 3mv0.pdb, 3mv1.pdb, 3vd3.pdb, 3vd4.pdb, 3vd5.pdb, 3vd7.pdb, 3vd9.pdb, 3vda.pdb, 3vdb.pdb, 3vdc.pdb, 4ttg.pdb, 6tsh.pdb, 6tsk.pdb, 6tte.pdb, 7btk.pdb, 4cu6.pdb, 4cu7.pdb, 4cu8.pdb, 1bhg.pdb, 3hn3.pdb, 2je8.pdb, 2vqu.pdb, 2vzs.pdb, 2x05.pdb, 2x09.pdb, 1dyo.pdb, 1h6x.pdb, 1h6y.pdb, 1xna.pdb, 1xnt.pdb, 3k75.pdb, 3k77.pdb, 3lqc.pdb, 1gqp.pdb, 1jhj.pdb, 1tvg.pdb, 1xpw.pdb, 2itx.pdb, 4j1l.pdb, 1z3j.pdb, 2clt.pdb, 2jsd.pdb, 2w0d.pdb, 2y6c.pdb, 2y6d.pdb, 3f15.pdb, 3f16.pdb, 3f17.pdb, 3f18.pdb, 3f19.pdb, 3f1a.pdb, 3lk8.pdb, 3lka.pdb, 3ma2.pdb, 3nx7.pdb, 3ts4.pdb, 3tsk.pdb, 3tt4.pdb, 3uvc.pdb, 3v96.pdb, 4efs.pdb, 4gql.pdb, 4gr0.pdb, 4gr3.pdb, 4gr8.pdb, 4h30.pdb, 4h49.pdb, 4h76.pdb, 4h84.pdb, 4i03.pdb, 4ilw.pdb, 5h0u.pdb, 1eb6.pdb, 1g12.pdb, 1ge5.pdb, 1ge6.pdb, 1ge7.pdb, 1iag.pdb, 1etp.pdb, 1h1o.pdb, 1fcd.pdb, 3vrd.pdb, 1iqc.pdb, 1eb7.pdb, 2vhd.pdb, 1nml.pdb, 1rz5.pdb, 1rz6.pdb, 1h31.pdb, 1h32.pdb, 1h33.pdb, 2oz1.pdb, 1pgn.pdb, 1pgo.pdb, 1pgp.pdb, 1pgq.pdb, 2pgd.pdb, 1pgj.pdb, 1txg.pdb, 1evy.pdb, 1evz.pdb, 1jdj.pdb, 1m66.pdb, 1m67.pdb, 1n1e.pdb, 1n1g.pdb, 1gu7.pdb, 1guf.pdb, 1gyr.pdb, 1h0k.pdb, 1n9g.pdb, 4was.pdb, 1h2b.pdb, 1rjw.pdb, 1cdo.pdb, 1p0c.pdb, 1p0f.pdb, 1a71.pdb, 1a72.pdb, 1adb.pdb, 1adc.pdb, 1adf.pdb, 1adg.pdb, 1axe.pdb, 1axg.pdb, 1bto.pdb, 1ee2.pdb, 1het.pdb, 1heu.pdb, 1hf3.pdb, 1hld.pdb, 1ju9.pdb, 1lde.pdb, 1ldy.pdb, 1mg0.pdb, 1mgo.pdb, 1n8k.pdb, 1n92.pdb, 1p1r.pdb, 1qlh.pdb, 1qlj.pdb, 1qv6.pdb, 1qv7.pdb, 1ye3.pdb, 2jhf.pdb, 2jhg.pdb, 2ohx.pdb, 2oxi.pdb, 3bto.pdb, 4dwv.pdb, 4dxh.pdb, 4nfs.pdb, 4ng5.pdb, 4xd2.pdb, 5adh.pdb, 5vj5.pdb, 5vjg.pdb, 5vkr.pdb, 5vl0.pdb, 5vn1.pdb, 6adh.pdb, 6nbb.pdb, 6owm.pdb, 6owp.pdb, 6xt2.pdb, 7adh.pdb, 7jqa.pdb, 7k35.pdb, 7ude.pdb, 8adh.pdb, 1agn.pdb, 1d1s.pdb, 1d1t.pdb, 1deh.pdb, 1hdx.pdb, 1hdy.pdb, 1hdz.pdb, 1hso.pdb, 1hsz.pdb, 1ht0.pdb, 1htb.pdb, 1m6h.pdb, 1m6w.pdb, 1ma0.pdb, 1mc5.pdb, 1mp0.pdb, 1teh.pdb, 1u3t.pdb, 1u3u.pdb, 1u3v.pdb, 1u3w.pdb, 2fze.pdb, 2fzw.pdb, 3hud.pdb, 1e3e.pdb, 1e3i.pdb, 1e3l.pdb, 1llu.pdb, 1jvb.pdb, 1nto.pdb, 1nvg.pdb, 1r37.pdb, 1jqb.pdb, 1kev.pdb, 1ped.pdb, 2b83.pdb, 1bxz.pdb, 1ykf.pdb, 2nvb.pdb, 1kol.pdb, 1qor.pdb, 1yb5.pdb, 1iyz.pdb, 1iz0.pdb, 1vj1.pdb, 1jvs.pdb, 1k5h.pdb, 1onn.pdb, 1ono.pdb, 1onp.pdb, 1q0h.pdb, 1q0l.pdb, 1q0q.pdb, 1t1r.pdb, 1t1s.pdb, 2egh.pdb, 1r0k.pdb, 1r0l.pdb, 1nvm.pdb, 1qki.pdb, 1dpg.pdb, 1e77.pdb, 1e7m.pdb, 1e7y.pdb, 1h93.pdb, 1h94.pdb, 1h9a.pdb, 1h9b.pdb, 2dpg.pdb, 1obf.pdb, 1gpd.pdb, 4gpd.pdb, 1dbv.pdb, 1gd1.pdb, 1npt.pdb, 1nq5.pdb, 1nqa.pdb, 1nqo.pdb, 2dbv.pdb, 2gd1.pdb, 3cmc.pdb, 3dbv.pdb, 4dbv.pdb, 1dc3.pdb, 1dc4.pdb, 1dc5.pdb, 1dc6.pdb, 1gad.pdb, 1gae.pdb, 1s7c.pdb, 2vyn.pdb, 2vyv.pdb, 6utm.pdb, 6utn.pdb, 6uto.pdb, 5za0.pdb, 5o0v.pdb, 3gpd.pdb, 1u8f.pdb, 1znq.pdb, 1ywg.pdb, 1zya.pdb, 2b4r.pdb, 2b4t.pdb, 1cf2.pdb, 2czc.pdb, 1j0x.pdb, 1crw.pdb, 1jn0.pdb, 1nbo.pdb, 1rm3.pdb, 1rm4.pdb, 1rm5.pdb, 2hki.pdb, 2pkq.pdb, 2pkr.pdb, 1b7g.pdb, 1hdg.pdb, 1cer.pdb, 2g82.pdb, 1vc2.pdb, 2x0n.pdb, 1k3t.pdb, 1ml3.pdb, 1qxs.pdb, 3dmt.pdb, 3ids.pdb, 1a7k.pdb, 1gyp.pdb, 1gyq.pdb, 1i32.pdb, 1i33.pdb, 2b0j.pdb, 3daf.pdb, 3dag.pdb, 3f46.pdb, 3f47.pdb, 3h65.pdb, 1jax.pdb, 1jay.pdb, 1wdk.pdb, 1wdl.pdb, 1wdm.pdb, 2d3t.pdb, 1f0y.pdb, 1f12.pdb, 1f14.pdb, 1f17.pdb, 1il0.pdb, 1lsj.pdb, 1lso.pdb, 1m75.pdb, 1m76.pdb, 2hdh.pdb, 3had.pdb, 3hdh.pdb, 1dli.pdb, 1dlj.pdb, 2csu.pdb, 2yv2.pdb, 1cqi.pdb, 1cqj.pdb, 1jkj.pdb, 1jll.pdb, 1scu.pdb, 2nu6.pdb, 2nu7.pdb, 2nu8.pdb, 2nu9.pdb, 2nua.pdb, 2scu.pdb, 6g4q.pdb, 6wcv.pdb, 2yv1.pdb, 1euc.pdb, 1eud.pdb, 2fp4.pdb, 2fpg.pdb, 2fpi.pdb, 2fpp.pdb, 4xx0.pdb, 5cae.pdb, 6xru.pdb, 7jfp.pdb, 7jj0.pdb, 7jkr.pdb, 7jmk.pdb, 1oi7.pdb, 1qle.pdb, 1m56.pdb, 1m57.pdb, 1fft.pdb, 1occ.pdb, 1oco.pdb, 1ocr.pdb, 1ocz.pdb, 1v54.pdb, 1v55.pdb, 2dyr.pdb, 2dys.pdb, 2eij.pdb, 2eik.pdb, 2eil.pdb, 2eim.pdb, 2ein.pdb, 2occ.pdb, 2y69.pdb, 2zxw.pdb, 3abk.pdb, 3abl.pdb, 3abm.pdb, 3ag1.pdb, 3ag2.pdb, 3ag3.pdb, 3ag4.pdb, 3asn.pdb, 3aso.pdb, 3wg7.pdb, 3x2q.pdb, 5b1a.pdb, 5b1b.pdb, 5b3s.pdb, 5iy5.pdb, 5w97.pdb, 5wau.pdb, 5x19.pdb, 5x1b.pdb, 5x1f.pdb, 5xdq.pdb, 5xdx.pdb, 5z84.pdb, 5z85.pdb, 5z86.pdb, 5zco.pdb, 5zcp.pdb, 5zcq.pdb, 6j8m.pdb, 6juw.pdb, 6jy3.pdb, 6jy4.pdb, 6nkn.pdb, 6nmf.pdb, 6nmp.pdb, 7coh.pdb, 7cp5.pdb, 7d5w.pdb, 7d5x.pdb, 7ev7.pdb, 7vvr.pdb, 7ate.pdb, 7atn.pdb, 7au3.pdb, 7au6.pdb, 1y7u.pdb, 1vpm.pdb, 3sps.pdb, 2gvh.pdb, 1z54.pdb, 2cye.pdb, 1yli.pdb, 1mka.pdb, 1mkb.pdb, 2cf2.pdb, 4keh.pdb, 4b0b.pdb, 4b0c.pdb, 4b0i.pdb, 4b0j.pdb, 4b8u.pdb, 4cl6.pdb, 4fq9.pdb, 6b7j.pdb, 5f6r.pdb, 5hd6.pdb, 3q62.pdb, 1tbu.pdb, 1c8u.pdb, 1iq6.pdb, 1s9c.pdb, 1pn2.pdb, 1pn4.pdb, 2gll.pdb, 2glm.pdb, 2glp.pdb, 2glv.pdb, 3b7j.pdb, 3cf8.pdb, 3cf9.pdb, 3d04.pdb, 3doy.pdb, 3doz.pdb, 3dp0.pdb, 3dp1.pdb, 3dp2.pdb, 3dp3.pdb, 3ed0.pdb, 4zjb.pdb, 6ihc.pdb, 1z6b.pdb, 1zhg.pdb, 2okh.pdb, 2oki.pdb, 3az8.pdb, 3az9.pdb, 3aza.pdb, 3azb.pdb, 1u1z.pdb, 2ess.pdb, 2own.pdb, 1gws.pdb, 1h29.pdb, 2cvc.pdb, 1czj.pdb, 3cao.pdb, 3car.pdb, 1w7o.pdb, 1aqe.pdb, 1gm4.pdb, 1gmb.pdb, 1i77.pdb, 1up9.pdb, 1upd.pdb, 2cy3.pdb, 2kmy.pdb, 2ksu.pdb, 3cyr.pdb, 1qn0.pdb, 1qn1.pdb, 1wad.pdb, 1gyo.pdb, 1a2i.pdb, 1it1.pdb, 1j0o.pdb, 1j0p.pdb, 1mdv.pdb, 1wr5.pdb, 2bpn.pdb, 2cdv.pdb, 2cth.pdb, 2cym.pdb, 2ewi.pdb, 2ewk.pdb, 2ewu.pdb, 2ffn.pdb, 2yxc.pdb, 2yyw.pdb, 2yyx.pdb, 2z47.pdb, 1ehj.pdb, 1f22.pdb, 1hh5.pdb, 1kwj.pdb, 1l3o.pdb, 1lm2.pdb, 1new.pdb, 2new.pdb, 1rwj.pdb, 1os6.pdb, 2ldo.pdb, 2mz9.pdb, 4haj.pdb, 4hb6.pdb, 4hb8.pdb, 4hbf.pdb, 4hc3.pdb, 4hdl.pdb, 19hc.pdb, 1ofw.pdb, 1ofy.pdb, 1duw.pdb, 2e84.pdb, 3bxu.pdb, 2lzz.pdb, 2n91.pdb, 3h33.pdb, 3h34.pdb, 3h4n.pdb, 3oue.pdb, 3sel.pdb, 3sj0.pdb, 3sj1.pdb, 3sj4.pdb, 3wmo.pdb, 1oah.pdb, 2rf7.pdb, 1gu6.pdb, 1qdb.pdb, 1fs7.pdb, 1fs8.pdb, 1fs9.pdb, 1bvb.pdb, 1ft5.pdb, 1ft6.pdb, 1h21.pdb, 1e39.pdb, 1jrx.pdb, 1jry.pdb, 1jrz.pdb, 1kss.pdb, 1ksu.pdb, 1lj1.pdb, 1m64.pdb, 1p2e.pdb, 1p2h.pdb, 1q9i.pdb, 1qjd.pdb, 1qo8.pdb, 1y0p.pdb, 2b7r.pdb, 2b7s.pdb, 1m1p.pdb, 1m1q.pdb, 1m1r.pdb, 6ee7.pdb, 1d4c.pdb, 1d4d.pdb, 1d4e.pdb, 1fgj.pdb, 1jni.pdb, 1ogy.pdb, 1sp3.pdb, 1ddc.pdb, 2j7a.pdb, 2vr0.pdb, 3l1t.pdb, 2rdz.pdb, 3tor.pdb, 4wjy.pdb, 4fas.pdb, 4n4n.pdb, 4n4o.pdb, 6m0p.pdb, 6m0q.pdb, 6hr0.pdb, 2k3v.pdb, 3ubr.pdb, 6p73.pdb, 2e80.pdb, 2e81.pdb, 3bnf.pdb, 3bng.pdb, 3bnh.pdb, 3bnj.pdb, 2bq4.pdb, 2a3m.pdb, 2a3p.pdb, 1z1n.pdb, 3ouq.pdb, 1b13.pdb, 1b2j.pdb, 1b2o.pdb, 1be7.pdb, 1bfy.pdb, 1c09.pdb, 1fhh.pdb, 1fhm.pdb, 1irn.pdb, 1iro.pdb, 1r0f.pdb, 1r0g.pdb, 1r0h.pdb, 1r0i.pdb, 1r0j.pdb, 1smm.pdb, 1smu.pdb, 1smw.pdb, 1t9o.pdb, 1t9p.pdb, 1t9q.pdb, 4rxn.pdb, 5rxn.pdb, 6nw0.pdb, 6nw1.pdb, 6rxn.pdb, 1e8j.pdb, 1rdj.pdb, 2dsx.pdb, 1rb9.pdb, 1rdv.pdb, 2kkd.pdb, 2qkz.pdb, 2ql0.pdb, 2rdv.pdb, 7rxn.pdb, 8rxn.pdb, 1dx8.pdb, 1h7v.pdb, 1yk4.pdb, 1yk5.pdb, 2pya.pdb, 3ss2.pdb, 1bq8.pdb, 1bq9.pdb, 1brf.pdb, 1caa.pdb, 1cad.pdb, 1iu5.pdb, 1iu6.pdb, 1qcv.pdb, 1rwd.pdb, 1vcx.pdb, 1zrp.pdb, 2pvx.pdb, 3kyu.pdb, 3kyv.pdb, 3kyw.pdb, 3kyx.pdb, 3kyy.pdb, 3ryg.pdb, 3rz6.pdb, 3rzt.pdb, 4ar3.pdb, 4ar4.pdb, 4ar5.pdb, 4ar6.pdb, 4k9f.pdb, 5ai2.pdb, 5ai3.pdb, 5nw3.pdb, 5ome.pdb, 6fo4.pdb, 1s24.pdb, 2pve.pdb, 1spw.pdb, 2m4y.pdb, 2v3b.pdb, 1m2y.pdb, 1cfm.pdb, 1e2v.pdb, 1e2w.pdb, 1e2z.pdb, 1ewh.pdb, 1tu2.pdb, 1ci3.pdb, 1ctm.pdb, 1hcz.pdb, 1r94.pdb, 1r95.pdb, 1s98.pdb, 1nwb.pdb, 2p2e.pdb, 1nm8.pdb, 1s5o.pdb, 1ndb.pdb, 1ndf.pdb, 1ndi.pdb, 1t7n.pdb, 1t7o.pdb, 1t7q.pdb, 2h3p.pdb, 2h3u.pdb, 2h3w.pdb, 2fy2.pdb, 2fy3.pdb, 2fy4.pdb, 2fy5.pdb, 1q6x.pdb, 1t1u.pdb, 1xl7.pdb, 1xl8.pdb, 1xmc.pdb, 1xmd.pdb, 1j77.pdb, 1p3t.pdb, 1p3u.pdb, 1p3v.pdb, 1sk7.pdb, 3wmno.pdb, 2pq6.pdb, 2vce.pdb, 2vch.pdb, 2vcu.pdb, 2vg8.pdb, 2acv.pdb, 2acw.pdb, 2c1x.pdb, 2c1z.pdb, 2c9z.pdb, 5nlm.pdb, 6su6.pdb, 6su7.pdb, 1o6c.pdb, 1f6d.pdb, 1vgv.pdb, 1v4v.pdb, 4fkz.pdb, 3ot5.pdb, 3dzc.pdb, 8ahe.pdb

Table S2: Krebs Cycle: Cys Residues from Buried Hydrophilic cluster

| **PDB_ID** | **Chain_ID** | **ResidueNo** | **BuriedFraction** | **rHpy** | **Modifications** | **Species** | **Protein Name** |  |  |
| --- | --- | --- | --- | --- | --- | --- | --- | --- | --- |
| 2b3x | A | 300 | 0.999 | 0.393 | thioether | Homo sapiens | Iron-responsive element binding protein 1 | | |
| 1nis | A | 358 | 0.881 | 0.233 | sulphenylation | Bos taurus | ACONITASE |  |  |
| 1b0k | A | 424 | 0.855 | 0.238 | thioether | Sus scrofa | PROTEIN (ACONITASE) | |  |
| 1b0k | A | 199 | 0.999 | 0.35 | metal-binding | Sus scrofa | PROTEIN (ACONITASE) | |  |
| 1b0j | A | 358 | 0.879 | 0.154 | thioether | Sus scrofa | ACONITATE HYDRATASE | |  |
| 1ami | A | 358 | 0.88 | 0.212 | sulphenylation | Bos taurus | ACONITASE |  |  |
| 1ami | A | 421 | 0.897 | 0.172 | metal-binding | Bos taurus | ACONITASE |  |  |
| 1aco | A | 358 | 0.878 | 0.215 | sulphenylation | Bos taurus | ACONITASE |  |  |

Table S3: Photosynthesis Cycle: Cys Residues from Buried Hydrophilic cluster

| **PDB_ID** | **Chain_ID** | **Residue No** | **Buried Fraction** | **rHpy** | **Modifications** | **Species** | | | **Protein Name** | | |
| --- | --- | --- | --- | --- | --- | --- | --- | --- | --- | --- | --- |
| 7nhq | A | 18 | 0.842 | 0.416 | thioether | Thermosynechococcus elongatus | | | Photosystem II protein D1 1 | | |
| 7nhp | C | 232 | 0.777 | 0.34 | thioether | Thermosynechococcus elongatus | | | Photosystem II CP43 reaction center protein | | |
| 7nhp | D | 71 | 0.96 | 0.329 | thioether | Thermosynechococcus elongatus | | | Photosystem II D2 protein | | |
| 7mh5 | H | 156 | 0.996 | 0.537 | thioether | Rhodobacter sphaeroides | | | Reaction center protein H chain | | |
| 7dxa | A | 125 | 0.89 | 0.189 | sulphenylation | Thermosynechococcus vulcanus | | | Photosystem II protein D1 | | |
| 7d1u | D | 71 | 0.973 | 0.316 | thioether | Thermosynechococcus vulcanus | | | Photosystem II D2 protein | | |
| 7d1t | A | 47 | 0.81 | 0.267 | thioether | Thermosynechococcus vulcanus | | | Photosystem II protein D1 | | |
| 7d1t | A | 47 | 0.809 | 0.268 | thioether | Thermosynechococcus vulcanus | | | Photosystem II protein D1 | | |
| 7d1t | C | 244 | 0.797 | 0.328 | metal-binding | Thermosynechococcus vulcanus | | | Photosystem II protein D1 | | |
| 7cou | B | 150 | 0.733 | 0.372 | thioether | Thermosynechococcus vulcanus | | | Photosystem II CP47 reaction center protein | | |
| 7cjj | D | 40 | 0.838 | 0.314 | thioether | Thermosynechococcus vulcanus | | | Photosystem II protein D1 | | |
| 7cjj | A | 47 | 0.818 | 0.261 | thioether | Thermosynechococcus vulcanus | | | Photosystem II protein D1 | | |
| 7cjj | D | 40 | 0.839 | 0.321 | thioether | Thermosynechococcus vulcanus | | | Photosystem II D2 protein | | |
| 7cjj | A | 18 | 0.993 | 0.385 | thioether | Thermosynechococcus vulcanus | | | Photosystem II protein D1 | | |
| 7cjj | V | 40 | 0.678 | 0.488 | thioether | Thermosynechococcus vulcanus | | | Cytochrome c-550 | | |
| 7cji | D | 71 | 0.98 | 0.344 | thioether | Thermosynechococcus vulcanus | | | Photosystem II D2 protein | | |
| 7cji | O | 44 | 0.982 | 0.355 | metal-binding | Thermosynechococcus vulcanus | | | Photosystem II manganese-stabilizing polypeptide | | |
| 7cji | C | 244 | 0.796 | 0.365 | metal-binding | Thermosynechococcus vulcanus | | | Photosystem II CP43 reaction center protein | | |
| 7cji | A | 18 | 0.991 | 0.434 | thioether | Thermosynechococcus vulcanus | | | Photosystem II protein D1 | | |
| 7c52 | M | 272 | 0.89 | 0.352 | thioether | Thermochromatium tepidum | | | Photosynthetic reaction center M subunit | | |
| 7c52 | C | 110 | 0.749 | 0.495 | thioether | Thermochromatium tepidum | | | Photosynthetic reaction center cytochrome c subunit | | |
| 6zid | C | 305 | 0.746 | 0.301 | thioether | Rhodopseudomonas viridis | | | Photosynthetic reaction center cytochrome c subunit | | |
| 6zid | C | 247 | 0.778 | 0.334 | thioether | Rhodopseudomonas viridis | | | Photosynthetic reaction center cytochrome c subunit | | |
| 6zid | C | 90 | 0.722 | 0.36 | thioether | Rhodopseudomonas viridis | | | Photosynthetic reaction center cytochrome c subunit | | |
| 6zid | C | 132 | 0.788 | 0.155 | thioether | Rhodopseudomonas viridis | | | Photosynthetic reaction center cytochrome c subunit | | |
| 6zi9 | C | 132 | 0.787 | 0.156 | thioether | Rhodopseudomonas viridis | | | Photosynthetic reaction center cytochrome c subunit | | |
| 6zi9 | M | 160 | 0.592 | 0.423 | thioether | Rhodopseudomonas viridis | | | Photosynthetic reaction center cytochrome c subunit | | |
| 6zi9 | L | 122 | 0.6 | 0.485 | thioether | Rhodopseudomonas viridis | | | Photosynthetic reaction center cytochrome c subunit | | |
| 6zi9 | C | 308 | 0.713 | 0.312 | thioether | Rhodopseudomonas viridis | | | Photosynthetic reaction center cytochrome c subunit | | |
| 6z5s | W | 33 | 0.706 | 0.282 | metal-binding | Rhodopseudomonas palustris | | | Light harvesting complex 1 Protein W | | |
| 6z5r | H | 155 | 0.726 | 0.449 | sulphenylation | Rhodopseudomonas palustris | | | H subunit of photosynthetic reaction center complex | | |
| 6jlp | d | 40 | 0.841 | 0.316 | thioether | Thermosynechococcus vulcanus | | | Photosystem II D2 protein | | |
| 6jlp | b | 112 | 0.786 | 0.284 | thioether | Thermosynechococcus vulcanus | | | Photosystem II CP47 reaction center protein | | |
| 6jlp | B | 150 | 0.742 | 0.394 | thioether | Thermosynechococcus vulcanus | | | Photosystem II CP47 reaction center protein | | |
| 6jlp | d | 71 | 0.978 | 0.33 | thioether | Thermosynechococcus vulcanus | | | Photosystem II D2 protein | | |
| 6jlp | D | 40 | 0.836 | 0.318 | thioether | Thermosynechococcus vulcanus | | | Photosystem II D2 protein | | |
| 6jlp | T | 12 | 0.709 | 0.287 | thioether | Thermosynechococcus vulcanus | | | Photosystem II reaction center protein T | | |
| 6jlo | D | 40 | 0.839 | 0.323 | thioether | Thermosynechococcus vulcanus | | | Photosystem II D2 protein | | |
| 6jln | d | 71 | 0.978 | 0.336 | thioether | Thermosynechococcus vulcanus | | | Photosystem II D2 protein | | |
| 6jln | c | 288 | 0.641 | 0.384 | thioether | Thermosynechococcus vulcanus | | | Photosystem II CP43 reaction center protein | | |
| 6jln | V | 40 | 0.701 | 0.519 | thioether | Thermosynechococcus vulcanus | | | Cytochrome c-550 | | |
| 6jlm | C | 288 | 0.637 | 0.412 | thioether | Thermosynechococcus vulcanus | | | Photosystem II CP43 reaction center protein | | |
| 6jlm | B | 150 | 0.722 | 0.406 | thioether | Thermosynechococcus vulcanus | | | Photosystem II CP47 reaction center protein | | |
| 6jll | T | 12 | 0.722 | 0.266 | thioether | Thermosynechococcus vulcanus | | | Photosystem II reaction center protein T | | |
| 6jll | a | 18 | 0.994 | 0.385 | thioether | Thermosynechococcus vulcanus | | | Photosystem II protein D1 | | |
| 6jll | d | 71 | 0.978 | 0.369 | thioether | Thermosynechococcus vulcanus | | | Photosystem II D2 protein | | |
| 6jll | c | 288 | 0.645 | 0.399 | thioether | Thermosynechococcus vulcanus | | | Photosystem II CP43 reaction center protein | | |
| 6jll | v | 40 | 0.672 | 0.449 | thioether | Thermosynechococcus vulcanus | | | Cytochrome c-550 | | |
| 6jlk | B | 150 | 0.728 | 0.405 | thioether | Thermosynechococcus vulcanus | | | Photosystem II CP47 reaction center protein | | |
| 6jlk | T | 12 | 0.712 | 0.275 | thioether | Thermosynechococcus vulcanus | | | Photosystem II reaction center protein T | | |
| 6jlj | c | 288 | 0.65 | 0.394 | thioether | Thermosynechococcus vulcanus | | | Photosystem II CP43 reaction center protein | | |
| 5zzn | b | 112 | 0.764 | 0.304 | thioether | Thermosynechococcus elongatus | | | Photosystem II reaction center protein T | | |
| 5zzn | T | 12 | 0.704 | 0.302 | thioether | Thermosynechococcus elongatus | | | Photosystem II CP47 reaction center protein | | |
| 5y5s | M | 272 | 0.894 | 0.337 | thioether | Thermochromatium tepidum | | | Photosynthetic reaction center M subunit | | |
| 5y5s | C | 247 | 0.85 | 0.135 | thioether | Thermochromatium tepidum | | | Photosynthetic reaction center cytochrome c subunit | | |
| 5xnl | b | 34 | 1 | 0.354 | thioether | Pisum sativum | | | Photosystem II CP47 reaction center protein | | |
| 5ws6 | o | 19 | 0.98 | 0.331 | thioether | Thermosynechococcus vulcanus | | | Photosystem II manganese-stabilizing polypeptide | | |
| 5ws6 | c | 244 | 0.783 | 0.337 | metal-binding | Thermosynechococcus vulcanus | | | Photosystem II CP43 chlorophyll protein | | |
| 5ws6 | b | 150 | 0.73 | 0.374 | thioether | Thermosynechococcus vulcanus | | | Photosystem II CP47 reaction center protein | | |
| 5ws5 | o | 19 | 0.982 | 0.339 | thioether | Thermosynechococcus vulcanus | | | Photosystem II manganese-stabilizing polypeptide | | |
| 5ws5 | O | 19 | 0.988 | 0.335 | thioether | Thermosynechococcus vulcanus | | | Photosystem II manganese-stabilizing polypeptide | | |
| 5v2c | V | 37 | 0.761 | 0.157 | thioether | Thermosynechococcus vulcanus | | | Cytochrome c-550 | | |
| 5v2c | V | 40 | 0.688 | 0.489 | thioether | Thermosynechococcus vulcanus | | | Cytochrome c-550 | | |
| 5v2c | D | 71 | 0.978 | 0.328 | thioether | Thermosynechococcus vulcanus | | | Photosystem II D2 protein | | |
| 5v2c | O | 44 | 0.986 | 0.366 | thioether | Thermosynechococcus vulcanus | | | Photosystem II manganese-stabilizing polypeptide | | |
| 5v2c | a | 18 | 0.995 | 0.383 | thioether | Thermosynechococcus vulcanus | | | Photosystem II protein D1 | | |
| 5v2c | B | 112 | 0.774 | 0.286 | thioether | Thermosynechococcus vulcanus | | | Photosystem II CP47 reaction center protein | | |
| 5tis | C | 256 | 0.648 | 0.392 | thioether | Thermosynechococcus elongatus | | | Photosystem II CP43 reaction center protein | | |
| 5tis | C | 470 | 0.856 | 0.293 | sulphenylation | Thermosynechococcus elongatus | | | Photosystem II CP43 reaction center protein | | |
| 5o4c | L | 129 | 0.841 | 0.173 | thioether | Rhodopseudomonas viridis | | | Reaction center protein L chain | | |
| 5nj4 | L | 122 | 0.592 | 0.493 | thioether | Rhodopseudomonas viridis | | | Reaction center protein L chain | | |
| 5mx2 | v | 40 | 0.687 | 0.523 | thioether | Thermosynechococcus elongatus | | | Cytochrome c-550 | | |
| 5mx2 | a | 18 | 0.995 | 0.387 | metal-binding | Thermosynechococcus elongatus | | | Photosystem II protein D1 1 | | |
| 5kai | A | 18 | 0.995 | 0.379 | thioether | Thermosynechococcus elongatus | | | Photosystem II protein D1 1 | | |
| 5kai | A | 47 | 0.799 | 0.263 | thioether | Thermosynechococcus elongatus | | | Photosystem II protein D1 1 | | |
| 5kaf | d | 71 | 0.978 | 0.349 | thioether | Thermosynechococcus elongatus | | | Photosystem II D2 protein | | |
| 5kaf | D | 40 | 0.849 | 0.355 | thioether | Thermosynechococcus elongatus | | | Photosystem II D2 protein | | |
| 5h2f | c | 288 | 0.675 | 0.354 | thioether | Thermosynechococcus elongatus | | | Photosystem II CP43 reaction center protein | | |
| 5h2f | b | 112 | 0.803 | 0.232 | thioether | Thermosynechococcus elongatus | | | Photosystem II CP47 reaction center protein | | |
| 5gti | V | 37 | 0.751 | 0.168 | thioether | Thermosynechococcus vulcanus | | | Cytochrome c-550 | | |
| 5gti | a | 47 | 0.804 | 0.27 | thioether | Thermosynechococcus vulcanus | | | Photosystem II D1 protein | | |
| 5gti | B | 150 | 0.746 | 0.362 | thioether | Thermosynechococcus vulcanus | | | Photosystem II CP47 reaction center protein | | |
| 5gti | o | 19 | 0.982 | 0.346 | thioether | Thermosynechococcus vulcanus | | | Photosystem II manganese-stabilizing polypeptide | | |
| 5gth | c | 244 | 0.777 | 0.348 | metal-binding | Thermosynechococcus vulcanus | | | Photosystem II CP43 reaction center protein | | |
| 5gth | o | 19 | 0.986 | 0.362 | thioether | Thermosynechococcus vulcanus | | | Photosystem II manganese-stabilizing polypeptide | | |
| 5gth | t | 12 | 0.706 | 0.3 | thioether | Thermosynechococcus vulcanus | | | Photosystem II reaction center protein T | | |
| 5gth | A | 47 | 0.807 | 0.27 | thioether | Thermosynechococcus vulcanus | | | Photosystem II protein D1 | | |
| 5b66 | a | 18 | 0.994 | 0.385 | thioether | Thermosynechococcus vulcanus | | | Photosystem II protein D1 | | |
| 5b66 | d | 71 | 0.982 | 0.341 | thioether | Thermosynechococcus vulcanus | | | Photosystem II D2 protein | | |
| 5b66 | a | 47 | 0.823 | 0.248 | thioether | Thermosynechococcus vulcanus | | | Photosystem II protein D1 | | |
| 5b66 | c | 288 | 0.668 | 0.378 | thioether | Thermosynechococcus vulcanus | | | Photosystem II CP43 reaction center protein | | |
| 5b66 | v | 37 | 0.763 | 0.155 | thioether | Thermosynechococcus vulcanus | | | Cytochrome c-550 | | |
| 5b66 | c | 244 | 0.798 | 0.322 | thioether | Thermosynechococcus vulcanus | | | Photosystem II CP43 reaction center protein | | |
| 5b5e | b | 112 | 0.782 | 0.289 | thioether | Thermosynechococcus vulcanus | | | Photosystem II CP47 reaction center protein | | |
| 5b5e | C | 244 | 0.8 | 0.315 | thioether | Thermosynechococcus vulcanus | | | Photosystem II CP43 reaction center protein | | |
| 5b5e | b | 150 | 0.736 | 0.395 | thioether | Thermosynechococcus vulcanus | | | Photosystem II CP47 reaction center protein | | |
| 5b5e | V | 37 | 0.763 | 0.18 | thioether | Thermosynechococcus vulcanus | | | Cytochrome c-550 | | |
| 4ub8 | b | 150 | 0.732 | 0.402 | thioether | Thermosynechococcus vulcanus | | | Photosystem II CP47 reaction center protein | | |
| 4ub8 | A | 47 | 0.829 | 0.232 | thioether | Thermosynechococcus vulcanus | | | Photosystem II protein D1 1 | | |
| 4ub8 | B | 112 | 0.769 | 0.302 | thioether | Thermosynechococcus vulcanus | | | Photosystem II CP47 reaction center protein | | |
| 4ub6 | b | 112 | 0.787 | 0.288 | thioether | Thermosynechococcus vulcanus | | | Photosystem II CP47 chlorophyll apoprotein | | |
| 4ub6 | c | 288 | 0.658 | 0.393 | thioether | Thermosynechococcus vulcanus | | | Photosystem II 44 kDa reaction center protein | | |
| 4ub6 | a | 125 | 0.873 | 0.184 | thioether | Thermosynechococcus vulcanus | | | Photosystem Q(B) protein | | |
| 4rcr | L | 247 | 0.88 | 0.425 | thioether | Rhodobacter sphaeroides | | | PHOTOSYNTHETIC REACTION CENTER | | |
| 4prc | C | 305 | 0.793 | 0.256 | metal-binding | Rhodopseudomonas viridis | | | REACTION CENTER | | |
| 4prc | L | 129 | 0.819 | 0.148 | thioether | Rhodopseudomonas viridis | | | REACTION CENTER | | |
| 4pj0 | b | 112 | 0.762 | 0.312 | metal-binding | Thermosynechococcus elongatus | | | CP47 protein | | |
| 4pj0 | C | 244 | 0.792 | 0.353 | metal-binding | Thermosynechococcus elongatus | | | Photosystem II CP43 protein | | |
| 4pj0 | b | 150 | 0.739 | 0.397 | thioether | Thermosynechococcus elongatus | | | CP47 protein | | |
| 4in6 | H | 156 | 1 | 0.535 | thioether | Rhodobacter sphaeroides | | | Reaction center protein H chain | | |
| 4il6 | a | 47 | 0.818 | 0.251 | thioether | Thermosynechococcus vulcanus | | | Photosystem Q(B) protein | | |
| 4h99 | H | 156 | 1 | 0.429 | thioether | Rhodobacter sphaeroides | | | Reaction center protein H chain | | |
| 3wu2 | B | 112 | 0.778 | 0.281 | thioether | Thermosynechococcus vulcanus | | | Photosystem II CP47 chlorophyll apoprotein | | |
| 3wu2 | b | 150 | 0.732 | 0.398 | thioether | Thermosynechococcus vulcanus | | | Photosystem II CP47 chlorophyll apoprotein | | |
| 3wu2 | C | 244 | 0.802 | 0.316 | thioether | Thermosynechococcus vulcanus | | | Photosystem Q(B) protein | | |
| 3wu2 | a | 47 | 0.839 | 0.232 | thioether | Thermosynechococcus vulcanus | | | Photosystem Q(B) protein | | |
| 3wu2 | A | 18 | 0.994 | 0.383 | thioether | Thermosynechococcus vulcanus | | | Photosystem II 44 kDa reaction center protein | | |
| 3wu2 | o | 19 | 0.984 | 0.342 | thioether | Thermosynechococcus vulcanus | | | Photosystem II manganese-stabilizing polypeptide | | |
| 3wu2 | V | 37 | 0.763 | 0.154 | thioether | Thermosynechococcus vulcanus | | | Cytochrome c-550 | | |
| 3t6e | C | 132 | 0.799 | 0.14 | thioether | Blastochloris viridis | | | Photosynthetic reaction center cytochrome c subunit | | |
| 3t6e | C | 305 | 0.763 | 0.259 | thioether | Blastochloris viridis | | | Photosynthetic reaction center cytochrome c subunit | | |
| 3t6d | M | 95 | 0.598 | 0.402 | thioether | Blastochloris viridis | | | Photosynthetic reaction center M-subunit | | |
| 3g7f | M | 160 | 0.609 | 0.437 | thioether | Blastochloris viridis | | | Photosynthetic reaction center M subunit | | |
| 3g7f | C | 244 | 0.811 | 0.222 | thioether | Blastochloris viridis | | | Photosynthetic reaction center cytochrome c subunit | | |
| 3g7f | L | 129 | 0.842 | 0.164 | thioether | Blastochloris viridis | | | Reaction center protein L chain | | |
| 3wu2 | C | 244 | 0.801 | 0.318 | thioether | Thermosynechococcus vulcanus | | | | | Oxygen-evolving photosysntem II |
| 3wu2 | A | 47 | 0.848 | 0.223 | thioether | Thermosynechococcus vulcanus | | | | Oxygen-evolving photosysntem II | |
| 3wu2 | V | 37 | 0.766 | 0.163 | thioether | Thermosynechococcus vulcanus | Oxygen-evolving photosysntem II | | | | |
| 3wu2 | t | 12 | 0.716 | 0.279 | thioether | Thermosynechococcus vulcanus | | Oxygen-evolving photosysntem II | | | |
| 2x5u | C | 247 | 0.787 | 0.281 | thioether | Blastochloris viridis | | | PHOTOSYNTHETIC REACTION CENTER CYTOCHROME C SUBUNIT | | |
| 2wx5 | H | 156 | 1 | 0.511 | thioether | Rhodobacter sphaeroides | | | REACTION CENTER PROTEIN H CHAIN | | |
| 2wjn | C | 308 | 0.734 | 0.292 | thioether | Rhodopseudomonas viridis | | | PHOTOSYNTHETIC REACTION CENTER CYTOCHROME C SUBUNIT | | |
| 2prc | C | 132 | 0.795 | 0.171 | metal-binding | Rhodopseudomonas viridis | | | PHOTOSYNTHETIC REACTION CENTER | | |
| 2i5n | C | 90 | 0.721 | 0.378 | thioether | Blastochloris viridis | | | Photosynthetic reaction center cytochrome c subunit | | |
| 2hg9 | H | 156 | 0.985 | 0.497 | metal-binding | Rhodobacter sphaeroides | | | Reaction center protein H chain | | |
| 2hg3 | H | 156 | 0.988 | 0.483 | metal-binding | Rhodobacter sphaeroides | | | Reaction center protein H chain | | |
| 1txw | C | 305 | 0.765 | 0.282 | thioether | Rhodopseudomonas viridis | | | REACTION CENTER | | |
| 1r2c | C | 135 | 0.713 | 0.354 | thioether | Rhodopseudomonas viridis | | | Photosynthetic reaction center cytochrome C subunit precursor | | |
| 1prc | C | 90 | 0.735 | 0.338 | metal-binding | Rhodopseudomonas viridis | | | PHOTOSYNTHETIC REACTION CENTER | | |
| 1mps | H | 156 | 0.999 | 0.445 | metal-binding | Rhodobacter sphaeroides | | | PHOTOSYNTHETIC REACTION CENTER | | |
| 1l9j | H | 156 | 1 | 0.448 | thioether | Rhodobacter sphaeroides | | | REACTION CENTER PROTEIN H CHAIN | | |
| 1l9j | L | 247 | 0.941 | 0.351 | thioether | Rhodobacter sphaeroides | | | REACTION CENTER PROTEIN L CHAIN | | |
| 1k6n | H | 156 | 1 | 0.339 | thioether | Rhodobacter sphaeroides | | | PHOTOSYNTHETIC REACTION CENTER H SUBUNIT | | |
| 1eys | C | 288 | 0.734 | 0.295 | thioether | Thermochromatium tepidum | | | PHOTOSYNTHETIC REACTION CENTER | | |
| 1dxr | M | 160 | 0.581 | 0.42 | thioether | Rhodopseudomonas viridis | | | PHOTOSYNTHETIC REACTION CENTER M SUBUNIT | | |
| 1dv3 | H | 156 | 0.994 | 0.399 | thioether | Rhodobacter sphaeroides | | | PHOTOSYNTHETIC REACTION CENTER REACTION CENTER | | |

Table S4: Pathogen names from parasitic infections

| **Liver Fluke** | **Hookworm** | **Parasitic worm** |
| --- | --- | --- |
| Schistosoma haematobium | Ancylostoma ceylanicum | Onchocerca volvulus |
| Fasciola hepatica | Necator americanus | Haemonchus contortus |
| Schistosoma japonicum |  | Heligmosomoides polygyrus |
| Schistosoma mansoni |  | Ascaris suum |
| Clonorchis sinensis |  | Wuchereria bancrofti |
|  |  | Trichinella spiralis |
|  |  | Brugia malayi |

| Pneumococcus (Streptococcus pneumoniae) | Pneumonia |
| --- | --- |
| Enterococcus faecalis | Human Gastrointenstinal Disease |
| Bacteroides fragilis | Diarrhoeal disease in humans |
| Veillonella parvula | Human Dental Caries and Periodontitis |
| Achromobacter sp | pneumonia, septicemia, peritonitis, urinary tract and other infections. |
| Aerococcus urinae | Human Urinary Tract Infections |
| Anthrax bacillus (Bacillus anthracis) | Anthrax Disease |
| Bacillus anthracis | Anthrax Disease |
| Bacillus cereus | Human GastroIntenstinal Disease |
| Brucella melitensis | Human Brucellosis |
| Campylobacter jejuni | Bacterial Diarrheal Illnesses |
| Clostridium botulinum | Human Botulinum |
| Helicobacter cinaedi | Human GastroIntestinal Track Infection |
| Helicobacter pylori | Human GastroIntestinal Track Infection |
| Mycobacterium smegmatis | Human Genital Secretions and Immunosuppresions |
| Mycobacterium tuberculosis | Human Lung Infection |
| Mycoplasma penetrans | Sexually Transmitted Disease |
| Pseudomonas aeruginosa | malignant external otitis, endophthalmitis, endocarditis, meningitis, pneumonia, and septicemia |
| Staphylococcus aureus | bloodstream infections, pneumonia, or bone and joint infections. |
| Streptococcus pyogenes | Human Strep Throat |
| Treponema pallidum | Syphilis |
| Vibrio cholerae | Cholera |
| Ureaplasma urealyticum | Ureaplasma STD |
| Listeria innocua | listeriosis |
| Lyme disease spirochete (Borrelia burgdorferi) | Lyme disease |
| Streptococcus suis | meningitis |
| Burkholderia pseudomallei | Melioidosis |
| Listeria monocytogenes | Listeriosis |
| Salmonella enterica | typhoid fever |
| Chlamydia trachomatis | chlamydia |
| Salmonella typhimurium | typhoid fever |
| Staphylococcus aureus | pneumonia, endocarditis, and osteomyelitis |
| Streptococcus pneumoniae | pneumoniae |
| Bacillus subtilis | bacteremia/septicemia, endocarditis, meningitis, and infections of wounds, the ears, eyes, respiratory tract, urinary tract, and gastrointestinal tract |
| Bordetella bronchiseptica | kennel cough, upper respiratory infection, or infectious tracheobronchitis |
| Burkholderia cepacia | cystic fibrosis or bronchiectasis |
| Klebsiella pneumoniae | pneumonia |
| Listeria monocytogenes | Listeriosis |
| Neisseria meningitidis | Meningococcal disease |
| Streptomyces scabies | Common Scab Potato |
| Clostridium perfringens | acute gastrointestinal infections |
| Clostridium thermocellum | pneumoniae |
| Pseudomonas aeruginosa | malignant external otitis, endophthalmitis, endocarditis, meningitis, pneumonia, and septicemia |
| Clostridium botulinum, serotype A | Botulism |
| Mycobacterium tuberculosis | Tuberculosis |
| Proteus mirabilis | Urinary tract infections |
| Sphingomonas paucimobilis | BSI, osteomyelitis, pyogenic arthritis, meningitis, urinary tract infection, and wound infection |
| Neisseria gonorrhoeae | Gonorrhoea |
| Neisseria meningitidis serogroup A | Meningococcal Disease |
| Burkholderia cenocepacia | cepacia infection |
| Shigella flexneri | bacillary dysentery |
| Bordetella parapertussis | pertussis |
| Acinetobacter baumannii | lung infections (“pneumonia”) and blood, wound, or urinary tract infections |
| Bartonella henselae | Cat scratch disease (CSD), also called cat scratch fever |
| Edwardsiella tarda | intestinal and extra-intestinal infections |
| Francisella tularensis | Tularemia, also known as “rabbit fever,” |
| Legionella pneumophila | pneumonia (Legionnaires' disease) and mild flu-like illness (Pontiac fever) |
| Xanthomonas campestris | black rot |
| Erwinia chrysanthemi | a soft rot pathogen degrading succulent fleshy plant organs such as roots, tubers, stem cuttings and thick leaves |
| Pseudomonas fluorescens | controlling sheath blight and blast of paddy, wilt diseases of redgram, and banana |
| Xanthomonas oryzae | rice bacterial blight disease |
| Pseudomonas syringae pv. phaseolicola | pathogens that infect the phyllosphere |
| Edwardsiella tarda | intestinal and extra-intestinal infections |
| Elizabethkingia anophelis | Pneumonia and/or bronchitis (lung or airway infections) |
| Francisella tularensis | Tularemia, also known as “rabbit fever,” |
| Legionella pneumophila | pneumonia (Legionnaires' disease) and mild flu-like illness (Pontiac fever) |
| Mannheimia haemolytica | acute pneumonia |
| Mycobacterium abscessus | pulmonary disease |
| Mycobacterium avium | MAC lung disease |
| Porphyromonas gingivalis | periodontitis |
| Rickettsia prowazekii | Epidemic typhus |
| Streptococcus gordonii | acute bacterial endocarditis |
| Streptococcus mutans | tooth decay and cavities |
| Vibrio vulnificus | vibriosis |
| Yersinia pseudotuberculosis | Pseudotuberculosis |
| Campylobacter jejuni | campylobacteriosis |
| Aeromonas hydrophila | gastroenteritis, septicemia and necrotizing fasciitis |
| Coxiella burnetii | Q fever |
| Ehrlichia chaffeensis | Lyme disease |
| Enterococcus faecali | UTIs, bacteremia, and infective endocarditis |
| Streptococcus mutans | tooth decay and cavities |
| Xanthomonas oryzae | rice bacterial blight disease |
| Enterococcus faecium | endocarditis, urinary tract infections, prostatitis, intra-abdominal infection, cellulitis, and wound infection as well as concurrent bacteremia |
| Clostridium tetani | Tetanus |
| Serratia marcescens | urinary and respiratory infections, endocarditis, osteomyelitis, septicemia, wound infections, eye infections, and meningitis |
| Tannerella forsythia | connective tissue destruction and alveolar bone resorption in periodontal disease (PD) |
| Staphylococcus aureus | skin and soft tissue infections such as abscesses (boils), furuncles, and cellulitis |
| Erwinia chrysanthemi | soft rot pathogen degrading succulent fleshy plant organs such as roots, tubers, stem cuttings and thick leaves |
| Clostridium botulinum, serotype A | Botulism |
| Nocardia farcinica | Nocardiosis |
| Pseudomonas syringae pv. phaseolicola | pathogens that infect the phyllosphere |
| Pasteurella multocida | fowl cholera in poultry, atrophic rhinitis in pigs, and hemorrhagic septicemia |
| Fairy-ring mushroom (Marasmius oreades) | foot disease |
| Mycobacterium marinum | tuberculosis-like illness in fish and can infect humans when injured skin is exposed to a contaminated aqueous environment. |
| Aerococcus viridans | urinary tract infections |
| Anthrax bacillus (Bacillus anthracis) | Anthrax |
| Clostridium cellulolyticum | gas gangrene, tetanus, botulism, pseudomembranous colitis and food poisoning |
| Clostridium histolyticum | gas gangrene, tetanus, botulism, pseudomembranous colitis and food poisoning |
| Clostridium josui | gas gangrene, tetanus, botulism, pseudomembranous colitis and food poisoning |
| Rhodococcus sp | subacute, necrotizing infection, mostly cavitary pneumonia and lung abscess |
| Streptococcus agalactiae | neonatal sepsis. |
| Streptococcus mitis | sepsis and septic shock |
| Xanthomonas citri | Citrus canker |
| Rhodococcus sp. mb1 | pneumonia |
| Proteus vulgaris | wound infections,urinary tract infections |

| **Organism** | **Viral Diseases** |
| --- | --- |
| Influenza C virus | Human |
| Haemophilus influenzae | pneumonia, septicaemia, meningitis, epiglottitis, septic arthritis, cellulitis, otitis media, and purulent pericarditis |
| Ectromelia virus | mousepox |
| Human betacoronavirus 2c EMC/2012 | covid |
| Human betacoronavirus 2c jordan-n3/2012 | covid |
| SARS coronavirus | covid |
| Severe acute respiratory syndrome coronavirus 2 | covid |
| Betacoronavirus england 1 | covid |
| Human coronavirus emc | covid |
| Human sars coronavirus | covid |
| Mastadenovirus H2 | gastroenteritis, conjunctivitis, acute respiratory outbreaks |
| Haemophilus influenzae | pneumonia, septicaemia, meningitis, epiglottitis, septic arthritis, cellulitis, otitis media, and purulent pericarditis, endocarditis, osteomyelitis, and peritonitis |

| **Organism** | **Fungal Diseases** |
| --- | --- |
| Fungus (Aspergillus aculeatus) | Fungal Plant Disease |
| Dactylium dendroides | cobweb disease |
| Malassezia sympodialis | Malassezia |
| Neosartorya fumigata | allergic bronchopulmonary aspergillosis (ABPA), aspergilloma, chronic pulmonary aspergillosis and invasive aspergillosis |
| Aspergillus niger | black mould' |
| Fusarium graminearum (Gibberella zeae) | Fusarium head blight |
| Dactylium dendroides | cobweb disease |
| Fairy-ring mushroom (Marasmius oreades) | foot disease |

| **Organisms** | **Protozoan Diseases** |
| --- | --- |
| Trichomonas vaginalis | STD |
| Entamoeba histolytica | intestinal amebiasis |
|  |  |
| Trypanosoma cruzi | Chagas disease |
| Trypanosoma brucei | sleeping sickness |
| African malaria mosquito (Anopheles gambiae) | dengue |
| Anopheles funestus | malaria |
|  |  |

Table S5: Disease, pathogens, anatomy, protein microenvironment clusters and Cys-Post-translational modifications

| **Diseases Type (counts of functional Cysteines)** | **Anatomy of the disease** | **Buried hydrophobic** | **Buried hydrophilic** | **Exposed hydrophilic** |
| --- | --- | --- | --- | --- |
| UTI ;  (n = 11) | Urinary Tract | 4; modification: **All;**  pathways: **ETC, Fe-S-Cluster Biogenesis**  (Aerococcus urinae)  (Ureaplasma urealyticum)  (Proteus vulgaris) | 7**;** modification: **Thioether and Metal-binding;**  pathways: **ETC, Fe-S-Cluster Biogenesis**  (Treponema pallidum)  (Pseudomonas mendocina)  (Enterococcus faecium) | Nil |
| Coronavirus;  (n = 87) | Lungs | 46; modification: **all;**  pathway: **fatty acid synthesis**  ( Severe acute respiratory syndrome 2)  ( Human sars)  ( SARS) | 27; modification: **all;**  pathway: **fatty acid synthesis**  (Severe acute respiratory syndrome 2)  (Human Betacoronavirus 2c Jordan-N3/2012)  (Betacoronavirus England 1)  ( SARS) | 14**;** modification: **thioether (11), sulfenylations (3)**  pathway: **fatty acid synthesis**  (Severe acute respiratory syndrome 2)  (Human sars)  ( Sars) |
| Botulism;  (n = 41) | Digestive | 28 ; modification: **Thioether and Disulphide;**  Pathway: **Fatty Acid Synthesis and Glutathione Metabolism**  (Clostridium botulinum) | 10; modification: **Thioether, Sulphenylation and disulfide;**  Pathway: **Fatty Acid Synthesis and Glutathione Metabolism**  (Clostridium botulinum) | 3; modification: **Sulphenylation and Metal-binding;**  Pathway: **Fatty Acid Synthesis and Glutathione Metabolism**  (Clostridium botulinum) |
| Pneumonia ;  (n = 105) | Lungs | 21; modification:  **Thioether, Disulphide and Metal-binding;** Pathway: **ETC, Glutathione Metabolism , Fe-S-Cluster Biogenesis**  (Pseudomonas aeruginosa)  (Klebsiella pneumoniae)  (Haemophilus influenzae)  (Streptococcus pneumoniae)  (Mannheimia haemolytica) | 77**;** modifications:  **metal-binding, thioether and Disulphide**  Pathway:  **All four**  (Pseudomonas aeruginosa)  (Haemophilus influenzae)  (Streptococcus pneumoniae) | 7; modifications:  **Thioether and Metal-binding**  Pathway: **all four**  (Pseudomonas aeruginosa) |
| Tuberculosis;  (n = 28) | Lungs | 18; modifications: **four;**  pathway **– all four**  (Mycobacterium tuberculosis) | 4; modifications: **thioether;**  pathway **– all four**  (Mycobacterium tuberculosis) | **6;**  modifications: **thioether;**  pathway **– all four**  (Mycobacterium tuberculosis) |
| Plague;  (n=12) | Skin | 6; modifications:  **thioether and disulfide;**  pathway- **ETC, Fe-S cluster biogenesis, fatty acid synthesis and glutathion metabolism**  (Yersinia pestis) | 6; modifications: **metal, disulfide and thioether;**  Pathway -  **ETC, Fe-S cluster biogenesis, fatty acid synthesis and glutathione metabolism**  (Yersinia pestis) | Nil |
| Meningococcal disease;  (n =129) | Brain | 43; modifications:  **all four modifications**  pathway-  **Electron Transport Chain, Fe-S-Cluster Biogenesis, Fatty Acid Synthesis and Glutathione Metabolism;**  (Pseudomonas aeruginosa, Haemophilus influenzae,Neisseria meningitidis | 79; modifications:  **Disulphide**  Pathway - **Electron Transport Chain, Fe-S-Cluster Biogenesis, Fatty Acid Synthesis and Glutathione Metabolism**  (Pseudomonas aeruginosa, Haemophilus influenzae,Neisseria meningitidis) | 7; modifications:  **Metal-binding and Thioether**  Pathway - **Electron Transport Chain, Fe-S-Cluster Biogenesis Glutathione Metabolism ,;**  **Fatty Acid Biosynthesis; (**Pseudomonas aeruginosa) |
| Maligant external Otitis;  (n=165) | Ear | 90; modifications:  **Metal-binding , Thioether and Sulphenylation**  Pathway**- Electron Transport Chain, Fe-S-Cluster Biogenesis, Fatty Acid Biosynthesis, Glutathione Metabolism**  (Pseudomonas aeruginosa) | 68 modifications- **Thioether, metal-binding and disulphide**  Pathway -  **Electron Transport Chain, Fe-S-Cluster Biogenesis, Fatty Acid Biosynthesis, Glutathione Metabolism**  (Pseudomonas aeruginosa) | 7  modifications- **metal-binding and thioether**  Pathway –  **Electron Transport Chain, Fe-S-Cluster Biogenesis, Fatty Acid Biosynthesis, Glutathione Metabolism**  (Pseudomonas aeruginosa) |
| Endophthalmitis  (n = 173) | Eye | 90  modifications - **All four modifications**;  Pathway -  **Electron Transport Chain, Fe-S-Cluster Biogenesis, Fatty Acid Biosynthesis, Glutathione Metabolism**  (Pseudomonas aeruginosa) | 76; modifications - **Thioether, metal-binding and disulphide**  Pathway - **Electron Transport Chain, Fe-S-Cluster Biogenesis, Fatty Acid Biosynthesis, Glutathione Metabolism**  (Pseudomonas aeruginosa) | 7; modifications - **metal-binding and thioether**  Pathway -  **Electron Transport Chain, Fe-S-Cluster Biogenesis, Fatty Acid Biosynthesis, Glutathione Metabolism**  (Pseudomonas aeruginosa) |
| Endocarditis  (n = 202) | Heart | 119; modifications -  **all four modifications**  Pathway -  **Electron Transport Chain, Fe-S-Cluster Biogenesis, Fatty Acid Biosynthesis, Glutathione Metabolism**  (Pseudomonas aeruginosa,Haemophilus influenzae,Streptococcus gordonii, Bacteroides fragilis) | 76; modifications - **Thioether , metal-binding, disulphide**  Pathway –  **Electron Transport Chain, Fe-S-Cluster Biogenesis, Fatty Acid Biosynthesis, Glutathione Metabolism**  (Pseudomonas aeruginosa, Haemophilus influenzae, Enterococcus faecium) | 7; modifications - **metal-binding and thioether**  Pathway –  **Electron Transport Chain, Fe-S-Cluster Biogenesis, Fatty Acid Biosynthesis, Glutathione Metabolism**  (Pseudomonas aeruginosa) |
| Septicemia  (n = 166) | Blood | 90; modifications - **all four modifications**  Pathway - **Electron Transport Chain, Fe-S-Cluster Biogenesis, Fatty Acid Biosynthesis, Glutathione Metabolism**  (Pseudomonas aeruginosa, Haemophilus influenzae) | 69; modifications - **Thioether, metal-binding and disulphide**  Pathway –  **Electron Transport Chain, Fe-S-Cluster Biogenesis, Fatty Acid Biosynthesis, Glutathione Metabolism**  (Pseudomonas aeruginosa, Haemophilus influenzae) | 7; modifications - **metal-binding and thioether**  Pathway –  **Electron Transport Chain, Fe-S-Cluster Biogenesis, Fatty Acid Biosynthesis, Glutathione Metabolism**  (Pseudomonas aeruginosa) |
| Plant diseases  (n=4) | Plant | 2; modifications –  **Thioether and Disulphide ;** Pathway - **Fatty Acid Synthesis, Electron Transport Chain and Glutathione Metabolism; Thioether and Disulphide ;**  (Xanthomonas oryzae, Streptomyces scabies) | 2; modifications -  **Thioether and disulphide** Pathway - **Fatty Acid Synthesis and Glutathione Metabolism; Thioether and disulphide;**  (Xanthomonas citri, Xanthomonas oryzae) | Nil |
| Soft Tissue Infection:  (n=24) | Tissue | Nil | 16**;** modifications - **all thioether modification;** Pathway – **Electron Transport Chain** (Shewanella Frigidimarina) | **8;** modifications - **thioether modification;**  Pathway -  **ElectronTransport**  **Chain**  (Shewanella Frigidimarina) |
| Biliary Tract Disease  (n = 23) | Gastric | Nil | 15; modifications – **Thioether**  Pathway -  **Electron Transport chain;**  (Shewanella frigidimarina) | 8; modifications –  **Thioether**  Pathway -  **Electron Transport chain; Thioether;**  (Shewanella frigidimarina) |
| Septic arthritis  (n=10) | Bone | 7; modifications –  **all four modifications;** Pathway –  **Fatty Acid Synthesis, Electron Transport Chain and Glutathione Metabolism, all four modifications**  ( Haemophilus influenzae) | 3; **modifications -** Thioether  Pathway -  **Electron Transport Chain, Glutathione Metabolism , Fe-S-Cluster Biogenesis and Fatty Acid Synthesis;**  (Haemophilus influenzae) | Nil |
| Cellulitis  (n=16) | Cellular | 10;  **Modifications -** all four modifications;  Pathway –  **Electron Transport Chain, Glutathione Metabolism , Fe-S-Cluster Biogenesis, Fatty Acid Synthesis**  (Haemophilus influenzae,  Staphylococcus aureus) | 6;  **Modifications - Thioether and metal binding**  Pathway –  **Electron Transport Chain, Glutathione Metabolism , Fe-S-Cluster Biogenesis, Fatty Acid Synthesis**  (Haemophilus influenzae, Staphylococcus aureus, Enterococcus faecium) | Nil |
| Peritonitis  (n=10) | Gastric | 7;  Modifications – **all four modifications**  Pathway **- Fatty acid, glutathione metabolism, electron transport chain; all four modifications;**  (Haemophilus influenzae) | 3; modifications – **Thioether**  **(Haemophilus influenzae);**  Pathway -  **Electron Transport Chain, Glutathione Metabolism , Fe-S-Cluster Biogenesis, Fatty Acid Synthesis**  (Haemophilus influenzae) | Nil |
| Osteomyelitis  (n=10) | Bone | 7; modifications -  **all four modifications**  Pathway - **Electron Transport Chain , Glutathione metabolism, Fatty acid biosynthesis;**  (Haemophilus influenzae) | 3; modifications -  **Thioether**  Pathway - **Electron Transport Chain, Glutathione Metabolism , Fe-S-Cluster Biogenesis, Fatty Acid Synthesis**  (Haemophilus influenzae) | Nil |
| Purulent pericarditis  (n=10) | Cardiac | 7; modifications **– all four modifications**  Pathway –  **Electron Transport Chain , Glutathione metabolism, Fatty acid biosynthesis; all four modifications ;**  (Haemophilus influenzae) | 3; modifications  **-Thioether**  **(Haemophilus influenzae);**  Pathway - **Electron Transport Chain, Glutathione Metabolism , Fe-S-Cluster Biogenesis, Fatty Acid Synthesis**  (Haemophilus influenzae) | Nil |
| Skin Infections:  (n=7) | Skin | 7 modifications -  **all 4 modifications**  Pathway -  **ETC, Fe-S cluster biogenesis, fatty acid synthesis and glutathione metabolism**  (sp: mycobacterium smegmatis, protein: bateroferritins; pseudomonas mendocina, protein toluene-4-monooxygenase; staphylococcus aureus, proteins thioredoxin | Nil | Nil |

Table S6: Comparison of the current CysDUF database with databases and prediction servers reported in the literature

| Serial No. | **Database/**  Features | Number of proteins | Number of cysteine Residues |
| --- | --- | --- | --- |
| 1 | CysDuF (current study) | 74 DUF proteins | 70,342 |
| 2 | **Disulphide bond database** | 10,568 | 41,846 |
| 3 | **dbPTM**  S-nitrosylation,S-palmitoylation | 35,947 | 5,272 |
| 4 | **DBDB**  Disulphide bridges | 356 | 2,042 |
| 5 | **dbPTM 3.0**  S-palmitoylation,Prenylation,S-nitrosylation,S-glutathionylation | 35,835 | 50,909 |
| 6 | **Cysteine Motifs Database (CMD)**  Flanking motifs with their secondary structure and propensity values | 1,74,597 | 8,78,000 cysteine motifs |
| 7 | **RedoxDB**  Disulphide,S-nitrosylation,S-glutathionylation,Sulphination,Unknown modifications with the information of Gene name, Description, Organism and Length | 2,315 | 2,308 |
| 8 | **Cys.sqlite**  Cysteine disulphide bonds | 95,000 | 5,00,000 Cysteines as structural conformers, and 2,65,000 disulfide bond conformations. |
| 9 | **iCysMod**  Eukaryotes for S-glutathionylation, Oxidation, S-nitrosylation, Disulphide, S-sulfhydration, S-nitrosylation, S-sulfinylation and S-palmitoylation | 31,843 | 85,747 |
| 10 | **CysModDB**  S-nitrosylation, S-sulfenylation,S-sulfinylation,S-sulfonylation, S-glutathionylation,Disulphide, S-persulfidation, S-palmitoyolation | 21,654 | 70,536 |
| 11 | **DSDBASE2.0**  Disulphide bonds | 1,53,944 | 2,16,096 – Native disulphides and 2,01,53,850 – Modelled disulphides |
| 12 | **dbPTM2022**  Regulatory networks and functional association of post-translational modifications. | 15,289 |  |
| 13 | **qPTMplants**  Quantitative cysteine post-translational modifications in plants | 3,425 | 5,314 |
| 14 | **CysDB**  A human cysteine database based on experimental quantitative chemoproteomics | 11,621 | 62,888 |
| 15 | **S-SulfPred**  Sensitive predictor to capture S-sulfenylation sites based on a resampling one-sided selection undersampling-synthetic minority oversampling technique | 700 | 1000 |
| 16 | **SVM-Sulfosite**  Support vector machine-based predictor for S-sulfenylation sites | 778 Human proteins | 900 positive sites and 6,858 negative sites |
| 17 | **SulCysSite**  Novel predictor has been developed for accurate identification of S-sulfenylation by the integrated of complementary features | 1096 | 1434 positive data  10476 negative data |
| 18 | **PredCSO**  Ensemble method for prediction of S-sulfenylation sites in proteins | 192 PDB Files | 228 CSO residues and 757 cysteine residues |
| 19 | **SulSite-GTB**  Identification of protein S-sulfenylation sites by fusing multiple feature information and gradient tree boosting | Carroll Lab, RedoxDB  and UniProtKB databases | 247  positive samples, and 9446 negative samples |
| 20 | **DeepCSO**  Deep-Learning Network Approach to Predicting Cysteine S-Sulphenylation Sites | H. Sapiens and A. thaliana  1130 proteins | 1537 Arabidopsis CSO sites |
| 21 | **MDD-SOH**  Exploiting maximal dependence decomposition to identify S-sulfenylation sites with substrate motifs | 1096 | 9059 cysteine residues |
| 22 | **iSulfCys**  Prediction of S-sulfenylation Sites in Proteins with Physicochemical Properties of Amino Acids | 778 Homo proteins | 1105 S-sulfenylated sites |
| 23 | **BioSemAF-BiLSTM**  Protein sequence feature extraction framework based on semantic and evolutionary information | 778 | 7,124 non-sulfenylation cysteine sites and 1,045 sulfenylation cysteine sites |
| 24 | **Sulf_FSVM**  Prediction of S-sulfenylation sites using mRMR feature selection and fuzzy support vector machine algorithm | - | 900 experimentally annotated S-sulfenylation cysteine sites and 6856 non-S-sulfenylation cysteine sites |
| 25 | **BiGRUD-SA**  Protein S-sulfenylation sites prediction based on BiGRU and self-attention | - | 1247 positive samples and 9446 negative  samples of S-sulfenylation sites. |
| 26 | **fastSulf-DNN**  Deep neural networks and biological subwords to detect protein S-sulfenylation sites | - | 900 S-sulfenylated peptides and 6856 non-S-sulfenylated peptides |
| 27 | **Fu-SulfPred**  Identification of Protein S-sulfenylation Sites by Fusing Forests via Chou's General PseAAC |  | 1931 positive, and 14884 negative |
| 28 | **SOH-PRED**  A new bioinformatics tool for the  characterization and prediction of human  S-sulfenylation sites | - | 1247 positive  samples, and 9446 negative samples |
| 29 | **PRESS**  Protein S-Sulfenylation server | - | 243 CSO and 777 CYS residues |
| 30 | **CYS_REC**  Program for Predicting SS-bonding States of Cysteines and disulphide briges in Protein Sequences. | - | 3000 positive and 3000 negative |
| 31 | **SOH-Site**  Incorporating evolutionary information and physicochemical properties to identify protein S-sulfenylation sites | - | 9513 cysteine residues |
| 32 | **SIMLIN**  Prediction of S-sulphenylation in the human proteome based on multi-stage ensemble-learning models | - | 10584 cysteine residues |
| 33 | **IonCom : Metal-binding prediction for Zn^2+^ , Cu^2+^ , Fe^2+^ , Fe^3+^ , Ca^2+^ , Mg^2+^ , Mn^2+^ , Na^+^ , K^+^** | 2100 | - |
| 34 | **Cyscon**  Disulfide-bonding prediction based on Support Vector Regression (SVR) | SPX Dataset: 1018  PDBCYS dataset: 1797 | - |
| 35 | **MetalPredator**  Web server to predict iron–sulfur proteins from protein sequences | PDB25-1  PDB25 (*E.coli*)  PDB 70 (*E.coli*) | - |
| 36 | **MIB**  Web server to predict metals Ca^2+^, Cu^2+^, Fe^3+^, Mg^2+^, Mn^2+^, Zn^2+^, Cd^2+^, Fe^2+^, Ni^2+^, Hg^2+^, Co^2+^, or Cu^+^ for the given PDB structure using the Fragment Transformation Method | 1496 polypeptide chains | - |
| 37 | **MIB2**  Web server to predict metals Ca^2+^, Cu^2+^, Fe^3+^, Mg^2+^, Mn^2+^, Zn^2+^, Cd^2+^, Fe^2+^, Ni^2+^, Hg^2+^, Co^2+^, Cu^+^, Au^+^, Ba^2+^, Pb^2+^, Pt^2+^, Sm^3+^ and Sr^2+^ for the given PDB structure using the Fragment Transformation Method | 6807 polypeptide chains | - |
| 38 | **Cy-Pred’s:**  algorithm and a web service for the analysis and prediction of cysteine reactivity | - | - |
| 39 | **MIND-S:**  Deep-learning prediction model for elucidating protein post-translational modifications in human diseases | - | - |
| 40 | **Cpipe:** a comprehensive computational platform for sequence and structure-based analyses of Cysteine residues | - | - |
| 41 | **DiANNA (DiAminoacid Neural Network Application)**  Web server for disulfide connectivity prediction | - | - |
| 42 | **DISULFIND**  Disulfide bonding state and cysteine connectivity prediction server (SVM Binary Classifier) | - | - |
| 43 | **BRIGED**  Disulfide bond prediction | - | - |
| 44 | **DiPro2.0**  Cysteine disulfide bond predictor based on two-dimensional recursive neural networks, and weighted graph matching | - | - |
| 45 | **DBCP**  Disulfide bonding connectivity pattern prediction without the prior knowledge of the bonding state of cysteines | - | - |
| 46 | **MetalDetectorv2.0**  Predicting the geometry of metal binding sites in CYS and HIS from protein sequence | - | - |
| 47 | **CHED and SeqCHED**  Prediction of Metal-binding sites in proteins | - | - |
| 48 | **mFASD**  Structure-based algorithm for discriminating different types of metal-binding sites | - | - |
| 49 | **Metal Explorer**  Machine learning-based method for predicting eight different types of metal-binding sites: Ca, Co, Cu, Fe, Ni, Mg, Mn, and Zn | - | - |
| 50 | **PSI-PRED (MetSite):** Metal-binding prediction tool for the given protein sequence |  |  |
| 51 | **DeepSSPred** |  |  |
